# New Pyridyl and Dihydroisoquinoline Alkaloids Isolated from the Chevron Nemertean *Amphiporus angulatus*

**DOI:** 10.3390/md22040141

**Published:** 2024-03-22

**Authors:** William R. Kem, Ferenc Soti, James R. Rocca, Jodie V. Johnson

**Affiliations:** 1 Department of Pharmacology and Therapeutics, College of Medicine, University of Florida, 1200 Newell Drive, Gainesville, FL 32610, USA; 2 Advanced Magnetic Resonance Imaging and Spectroscopy Facility, McKnight Brain Institute, University of Florida, Gainesville, FL 32610, USA; jrrocca@ufl.edu; 3 Mass Spectrometry Research and Education Center, Department of Chemistry, University of Florida, Gainesville, FL 32611, USA; jvj@chem.ufl.edu

**Keywords:** alkaloid, anabaseine, dihydroisoquinoline, isoanatabine, nemertean, nemertine, nicotinic acetylcholine receptor, toxin, venom

## Abstract

Nemertean worms contain toxins that are used to paralyze their prey and to deter potential predators. Hoplonemerteans often contain pyridyl alkaloids like anabaseine that act through nicotinic acetylcholine receptors and crustacean chemoreceptors. The chemical reactivity of anabaseine, the first nemertean alkaloid to be identified, has been exploited to make drug candidates selective for alpha7 subtype nAChRs. GTS-21, a drug candidate based on the anabaseine scaffold, has pro-cognitive and anti-inflammatory actions in animal models. The circumpolar chevron hoplonemertean *Amphiporus angulatus* contains a multitude of pyridyl compounds with neurotoxic, anti-feeding, and anti-fouling activities. Here, we report the isolation and structural identification of five new compounds, doubling the number of pyridyl alkaloids known to occur in this species. One compound is an isomer of the tobacco alkaloid anatabine, another is a unique dihydroisoquinoline, and three are analogs of the tetrapyridyl nemertelline. The structural characteristics of these ten compounds suggest several possible pathways for their biosynthesis.

## 1. Introduction

Hoplonemerteans (also called hoplonemertines) are a class of mostly marine worms belonging to the phylum Nemertea that capture their prey with a venomous proboscis. Their name is derived from the Greek word Hoplos meaning “armed”. Bacq discovered the presence of toxins in nemerteans [1,2]. Extracts of several hoplonemertean species activate cholinergic synapses, such as the neuromuscular junction and autonomic ganglia. He postulated that these activities were due to the presence of a compound that he named “amphiporine” after *Amphiporus lactifloreus* (*Al*), the most thoroughly investigated species. *Al* extracts were chemically and pharmacologically similar to the alkaloid nicotine. Alkaloid purification at that time was largely based on fractional crystallization with picric or other acids to form sparingly soluble salts. A picrate salt was obtained from *Al* but in an amount insufficient for structural analysis [3]. 

**Figure 1:**
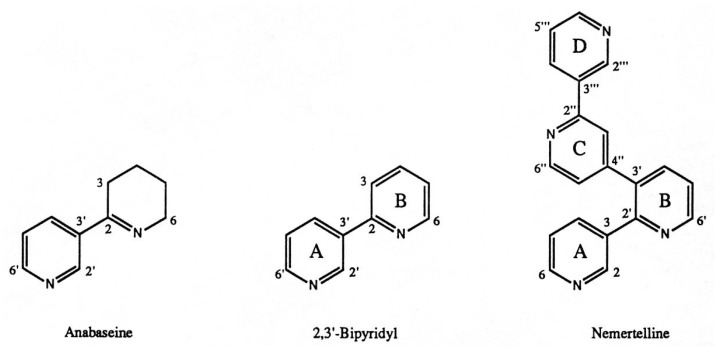
Structures of three previously identified pyridyl alkaloids of *Amphiporus angulatus*. A fourth *Aa* alkaloid, 3-methyl-2,3′-BP, has the same structure as 2,3′-BP, except that a methyl group is attached at the 3 position. Notice nomenclature for positions on rings A to D in nemertelline.

Three decades later, several hoplonemertean species were shown to contain pyridyl alkaloids with chemical and pharmacological properties similar to amphiporine. Anabaseine, 2-(3-pyridyl)-3,4,5,6-tetrahydropyridine (Figure 1) was the first nemertean alkaloid to be chemically identified [4,5]. Large amounts of anabaseine were found in the proboscis and body integument of the peregrine nemertean, *Paranemertes peregrina*, a relatively common hoplonemertean along the Pacific coasts of the USA and Canada [6]. Anabaseine is of considerable pharmacological interest as it is a potent nicotinic acetylcholine receptor agonist, though relatively unselective for a particular nAChR subtype [7,8]. An anabaseine derivative (GTS-21) selectively activates alpha7 nAChRs and has been the subject of several clinical tests as a potential drug for treating Alzheimer’s disease and schizophrenia [9,10,11]. GTS-21 has also become a favorite chemical tool for investigating the involvement of the alpha7 nicotinic acetylcholine receptor (nAChR) in cognition and other processes including inflammation due to its selective activation of this receptor subtype and ability to enter the brain [12,13,14].

**Figure 2:**
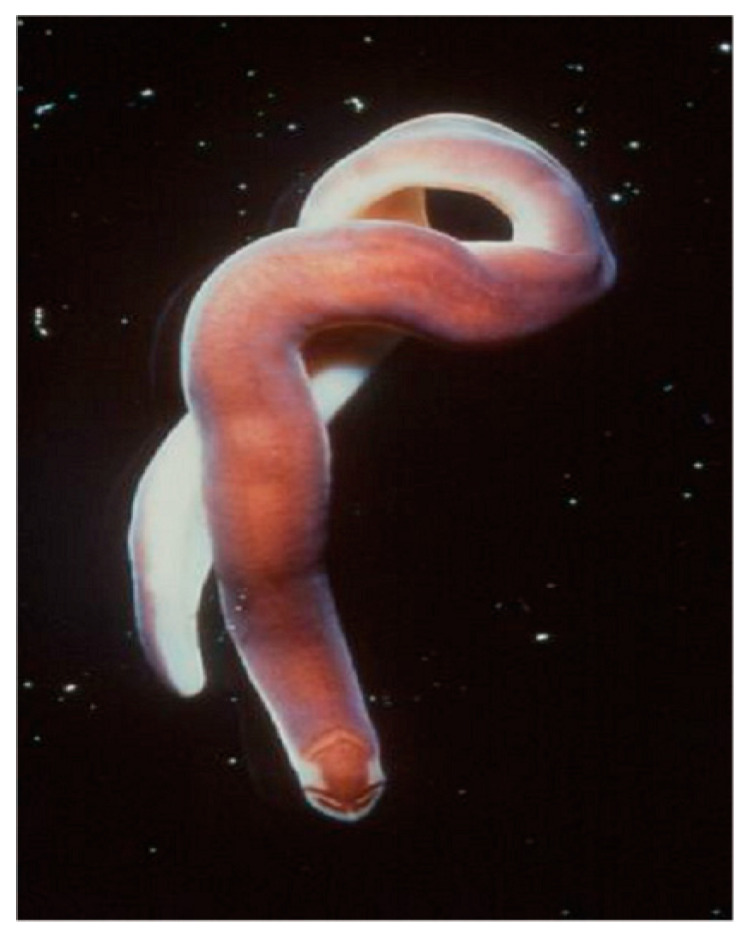
The bottom-dwelling chevron nemertean *Amphiporus angulatus* lives under rocks within the intertidal zone and in shallow water at northern latitudes. It feeds upon small crustaceans, which it paralyzes with a venomous proboscis. It is readily identified by the white chevron pattern on its neck, a purple dorsal surface, and white ventral surface. Adults can attain a length of 10 cm and fresh weight of 2 g. (Photograph courtesy of N. A. Meinkoth.).

The initial studies on nemertean toxins focused on abundant, relatively large, and easily collected species so that sufficient quantities of toxins could be isolated for structural and pharmacological characterization. One of the largest hoplonemertean species available to us was the chevron nemertean *Amphiporus angulatus* (*Aa*, Figure 2), which has a circumpolar distribution from the New England Atlantic coast north to Iceland then west through the NW passage of Canada and south along the northern American Pacific coasts. The two most abundant alkaloids (Figure 1) of this species, 2,3′-bipyridyl (2,3′-BP) and the tetrapyridyl nemertelline, were isolated via preparative layer chromatography and identified via mass spectrometric (MS) and nuclear magnetic resonance (NMR) analyses [15]. Nemertelline was so named because it is structurally similar to the tobacco alkaloid nicotelline, a tripyridyl which lacks the A ring of nemertelline. Only small amounts of anabaseine are present in *Aa*. The fourth *Aa* alkaloid to be identified was the volatile 3-methyl-2,3′-BP [16]. Both BPs potently paralyze crustaceans, which are thought to be the major prey of this hoplonemertean. Nemertelline displays a weak paralytic activity when injected into crayfish, but none when injected into mice. All of these pyridyls activate crustacean chemoreceptors involved in the recognition of potential prey [17]. They also inhibit barnacle larvae settlement (biofouling) and therefore have potential as commercial biofouling inhibitors [18,19].

Other pyridyl alkaloids were detected in the initial *Aa* alkaloid investigations but could not be purified via conventional low pressure liquid chromatographic methods in sufficient amounts for structural identification with the MS and NMR instruments available at that time. However, we were eventually able to collect several hundred members of this nemertean species, enabling the isolation and structural identification of additional alkaloids, which are the subjects of this article. 

## 2. Results

### 2.1. Bipyridyl Compounds

**Figure 3:**
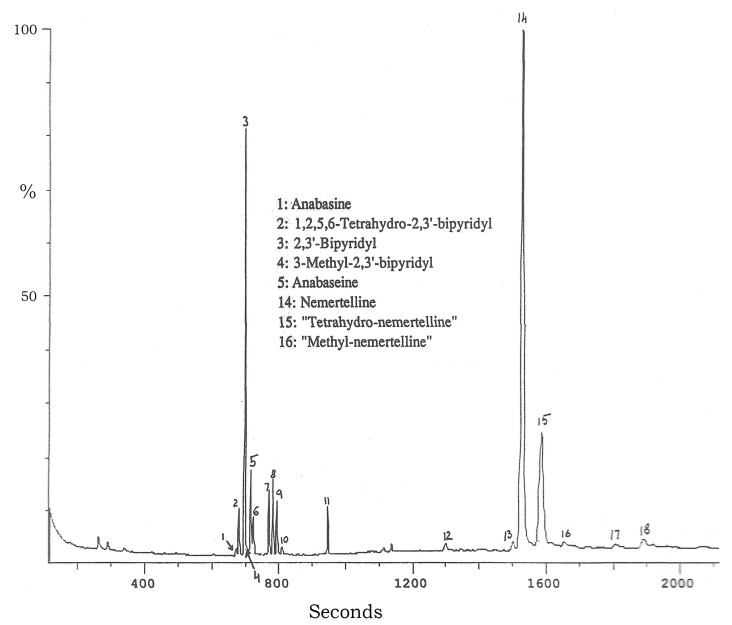
GC-MS profile of the Aa extract basic chloroform phase alkaloids. Compounds in peaks 6–13 were not identified. The peak 15 compound, a tetrahydronemertelline, was the third-most-abundant compound.

A GC-EIMS analysis (Figure 3) of the Aa extract basic chloroform phase revealed numerous (>20) peaks, most of which appeared, from their EI mass spectra, to be pyridyl alkaloids. The initial cluster of peaks was found to be due to bipyridyl-type alkaloids with different degrees of 2-pyridyl ring saturation. The dominant peak 3 was due to 2,3′-BP. The GC peak 1 molecular ion was *m*/*z* 162; its HPLC retention time and 70 eV EI fragmentation spectrum were essentially identical to those of the tobacco alkaloid anabasine, already known to occur in some hoplonemerteans [20] and ants [21].

**Figure 4:**
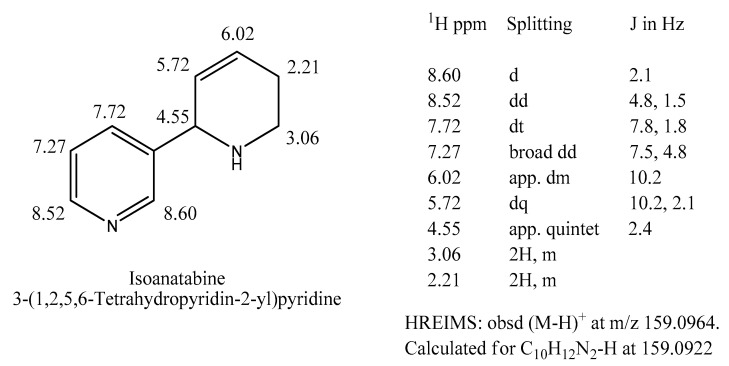
Isoanatabine: structure, proton NMR assignments, and high-resolution-EI-MS-determined elemental composition. See NMR spectrum in Supplementary Materials (page S1).

The GC peak 2 molecular ion was *m*/*z* 160, the same as for anabaseine (peak 5). However, its EIMS fragmentation spectrum (Table 1) was distinctly different from those of anabaseine and the tobacco alkaloid anatabine, but nearly identical to the EIMS data for the anatabine isomer 1,2,5,6-tetrahydro-2,3′-bipyridyl [22,23]. The elemental composition of the peak 2 compound (See bottom of Figure 4) was the same as that of anatabine, 1,2,5,6-tetrahydro-2,3′-bipyridyl and anabaseine.

Conventional silica gel or aluminum oxide chromatography of the Aa extract basic chloroform phase compounds revealed a multitude of putative pyridyl alkaloid peaks, with 2,3′-BP and nemertelline, again, being the most abundant. Most previously identified compounds, including anabaseine, anabasine, 2,3′-BP, 3-Me-2,3′-BP, and nemertelline, could be tentatively recognized through their UV absorbance spectra. Approximately 1.6 mg of the compound corresponding to the GC-MS peak 2 was obtained via conventional normal-phase SG column chromatography; this was sufficient to acquire a proton NMR spectrum (Supplementary Materials, page S1); see Figure 4 for a summary of the data. The aromatic proton peaks revealed the presence of a single 3-pyridyl moiety with signals essentially identical to those of the tobacco alkaloid anabasine. Protons from the other ring included two olefinic multiplet proton signals at 6.02 and 5.72 ppm, both with coupling constants of 10.2 Hz, indicating the presence of a cis double bond. An apparent quintet at 4.55 ppm with a 2.5 Hz coupling constant was assigned as a methine proton with neighboring aromatic ring, NH, and olefin groups. The multiplet lines at 2.05–2.35 and 2.96–3.15 ppm, each with two proton intensities, were assigned as methylenes next to an olefinic bond and a nitrogen atom, respectively. These interpretations are in good agreement with the structure shown in Figure 4. Direct comparisons of the NMR and MS spectra of the *Aa* alkaloid free base and the synthetic 1,2,5,6-tetrahydro-2,3’-bipyridyl free base, generously provided by Prof. L. Overman, demonstrated that they were the same coupound [21,22]. We named it isoantabine [23], as it is isomeric with anatabine and differs only slightly in the position of the piperidinyl ring double bond (between carbons at 3 and 4, instead of 4 and 5 as in anatabine). HPLC-MS examination of the *Aa* extract alkaline chloroform phase mixture also indicated the presence of a very small peak 4 alkaloid with *m*/*z* 170 M^+^. Its amount relative to the 2,3′-BP peak content was estimated to be <1%. Its EI-MS spectrum was in good agreement with that of 3-methyl-2,3′-BP [16]. The NMR spectrum of the isolated 2,3′-BP fraction contained a small singlet at 2.39 ppm. By adding a trace amount of this fraction to samples containing one of the eight possible methyl-2,3′-BP isomers prepared for pharmacological studies, it was found that this signal was only at the same position in the 3-methyl-2,3′-BP isomer. Further analyses led to its identification as 3-methyl-2,3′-BP [17]. Later, it was observed that the amount of this volatile compound in *Aa* crude extracts was much higher (~25% of 2,3′-BP) when the organic solvent evaporations were carried out below 30 °C.

### 2.2. Angulatine, A Novel Dihydroisoquinoline Alkaloid

**Figure 5:**
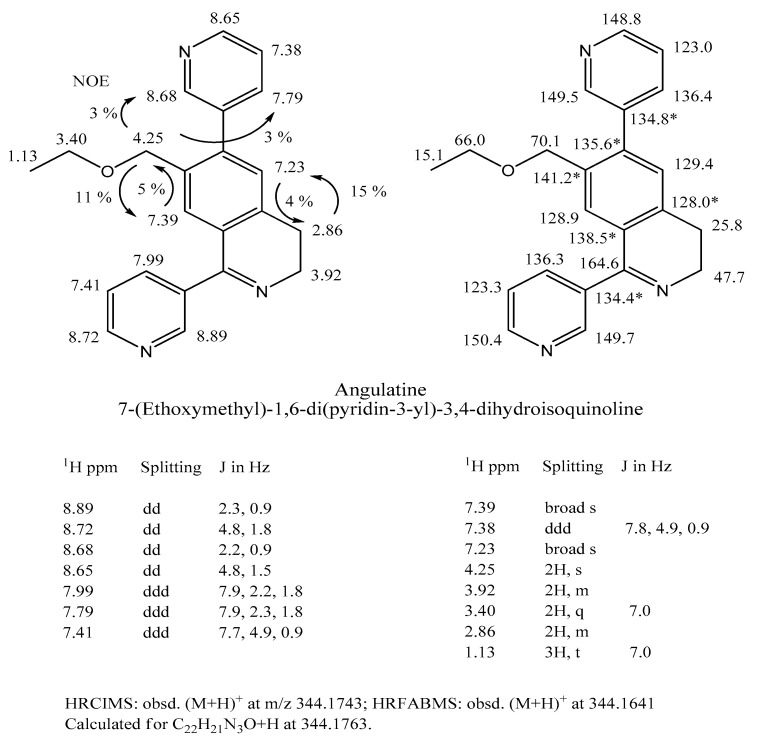
Angulatine structure, with proton and carbon NMR assignments. Its elemental composition was determined via HR-CI-MS and HR-FAB-MS. Additional NMR spectral data can be found in the Supplementary Materials, pages S2–S5.

Although it was unstable under GC-EI-MS conditions, this compound was detected when the LC-ESI-MS analysis of *Aa* alkaloids revealed a compound with an *m*/*z* 344 [M + H]^+^ ion corresponding to a free base MW = 343. It was eventually isolated from the *Aa* extract basic chloroform phase via repeated SG column chromatography under basic conditions. The resulting 2.5 mg sample was exhaustively examined via COSY, NOESY, HMQC, HMBC, and TOCSY NMR protocols. The structure of the free base was revealed via the NMR analyses to be a trisubstituted dihydroisoquinoline (Figure 5). High resolution mass spectrometry indicated that its empirical formula was C_22_H_21_N_3_O. Its proton NMR spectrum clearly indicated the presence of two 3-pyridyl moieties and two aromatic singlets at 7.39 and 7.24 ppm in the aromatic region, while in the aliphatic region there was a methylene singlet at 4.25 ppm, two methylene doublet-doublets at 3.97 and 2.92 ppm and resonances of an ethoxy group. Using decoupling experiments we determined which lines belong to the individual 3-pyridyl rings, and that the two methylene doublet-doublets are coupled with each other. The TOCSY spectrum revealed small and/or long-range couplings among the two aromatic singlets, the methylene singlet and the two methylene doublet-doublets (See Supplementary Materials, pages S2–S5). While the positions of the proton resonances in one of the 3-pyridyl rings were very similar to those in the 3-pyridyl ring of anabaseine, the resonances due to the other 3-pyridyl ring were more similar to the pyridyl resonances of 3-phenyl-pyridine. The position of the methylene singlet indicated that the methylene and ethoxy groups constitute an ethoxymethylene moiety connected to an aromatic ring. The position and the shape of the two methylene doublet-doublets were characteristic of those found in other 3,4-dihydroisoquinolines. Two possible structures were consistent with these assignments. NOE experiments carried out to distinguish between them clearly revealed the proximity of the “doublet-doublet” methylene hydrogens at 2.85 ppm to the pyridyl singlet at 7.24 ppm and the proximity of the “singlet” methylene hydrogens at 4.25 ppm to the aromatic proton at 7.39 ppm. These data identified the *Aa* compound as 1,6-di(pyrid-3-yl)-7-ethoxymethyl-3,4-dihyroisoquinoline. To our knowledge, this is the first time that a 3,4-dihydroisoquinoline alkaloid of this type has been found in nature.

### 2.3. New Nemertellines

**Figure 6:**
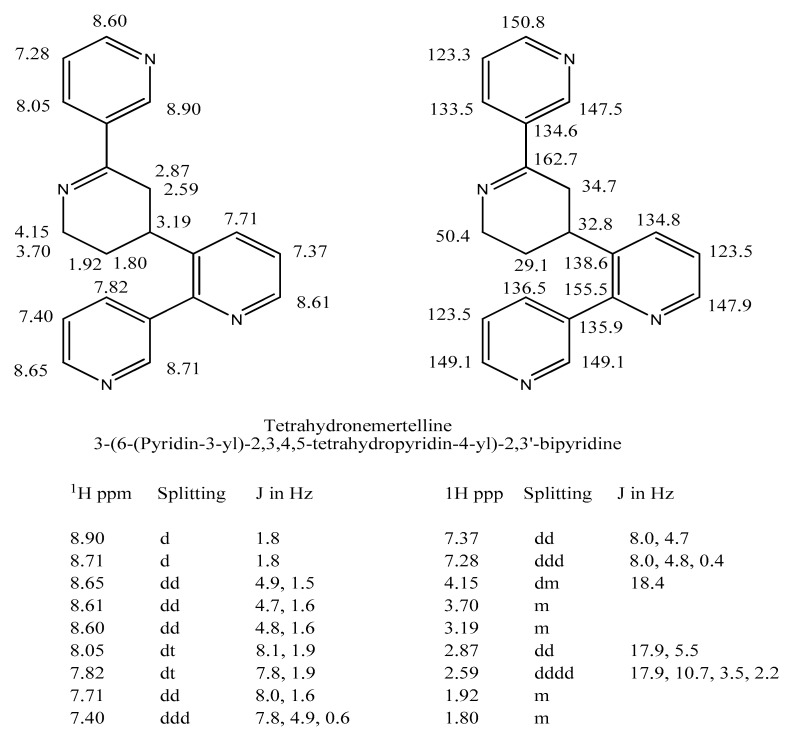
Tetrahydronemertelline structure and the NMR spectroscopic data used for its identification. Additional proton and ^13^C NMR spectral data can be found in the Supplementary Materials (pages S6–S21).

The initial GC-EIMS analysis (Figure 3) of the crude basic chloroform phase fractionrevealed the presence of several compounds with masses similar to nemertelline (free base *m*/*z* 310). These were subsequently isolated via SG chromatography. The most abundant nemertelline analog (*m*/*z* 314 M^+.^) displayed an EIMS fragmentation pattern similar to nemertelline (Table 2). It was shown via extensive NMR analyses to have the same ring connections as nemertelline. The additional four hydrogens were located in the C ring (Figure 6). The structure of this compound, named tetrahydronemertelline, was determined via multi-dimensional NMR methods (see Supplementary Materials pages S6–S21 for the NMR data). 

Insufficient amounts of methylnemertelline and hydroxynemertelline were obtained for the unequivocal identification of their structures, but, since their EIMS fragmentation patterns (Table 2) were similar to nemertelline, we suspect that these methyl and hydroxy substituents are located at the nemertelline C ring 3 position.

### 2.4. Two DehydroNemertellines 

**Figure 7:**
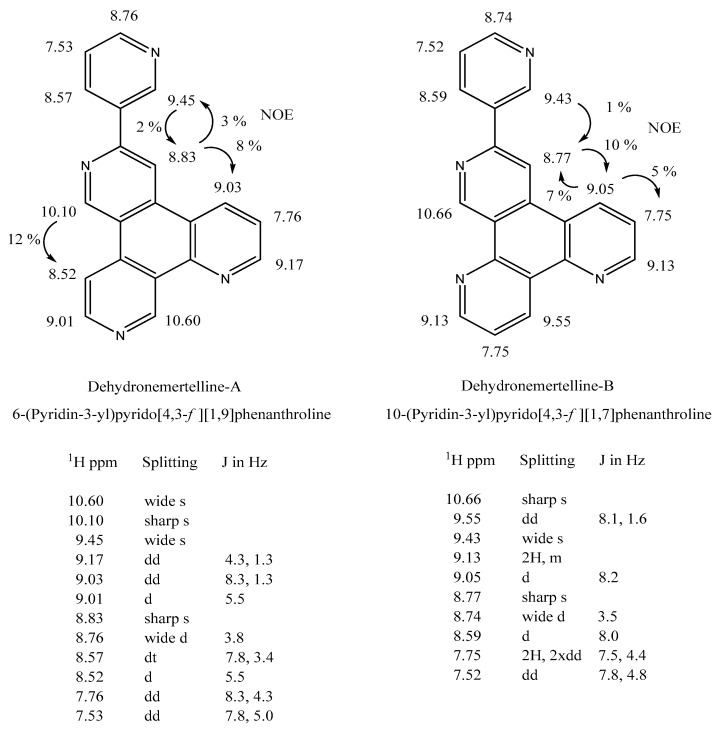
Structures of *Aa* dehydronemertellines A and B and proton NMR data used for the structure determination. See Supplementary Materials pages S22–S31 for additional NMR data, including for ^13^C resonances. S31 has a table of proton-carbon correlations for each dehydronemertelline.

Two isomeric nemertelline derivatives with identical *m*/*z* 308 molecular ions and common (but unique) UV absorbance spectra were observed to elute before nemertelline when isolated as free bases via normal phase SG LC in the presence of diethylamine. NMR analyses (including the HMBC and HSQC spectral determinations of ^13^C peaks; see Supplementary Materials pages S22–S31) showed that they have the same basic inter-ring connectivity as nemertelline, but the 2- or 4-position hydrogen in the A ring has been replaced with a carbon–carbon bond with the 5” carbon in ring C (Figure 7). Unlike nemertelline, these two compounds displayed two additional small (~20% magnitude relative to their peak absorbance at 260 nm) absorbance peaks at 330 and 345 nm. Space-filling molecular models of the two dehydronemertellines compounds allowed the co-planarity of the A ring with the C and D rings, whereas, in nemertelline, the A ring was twisted out of the plane of the B and C rings. 

Although the ^13^C chemical shifts for nemertelline have been reported [24,25], the positions of the carbons in the nemertelline structure were not assigned. Therefore, we carried out such correlations and have assigned ^13^C values to 19 of the 20 carbons present (see Supplementary Materials pages S32–S37 for the spectra and table). This new data should aid in the interpretation of data for related compounds in future studies. Also, they indicate that the addition of the additional C-C bond between rings A and C in the two dehydronemertellines produces considerable changes in the chemical shifts of carbons and protons in the A, B, and C rings of these two compounds relative to nemertelline.

## 3. Discussion

### 3.1. Functional Considerations

The remarkable number of pyridyl alkaloids in this species needs to be discussed. *Aa* is a relatively large benthic (bottom-dwelling) hoplonemertean. It cohabits the crevices under rocks in the lower intertidal zone and below with a variety of other organisms. It is thought to primarily feed on amphipod crustaceans, which it paralyses with its proboscis venom apparatus. It must also deter the predatory crabs, fish (blennies), mollusks, and other animals that occupy the same space. 2,3′-BP, the most abundant bipyridyl alkaloid, potently (and rapidly) paralyses crayfish upon injection into their vascular system and is even more potent (~4×) than anabaseine. However, this alkaloid does not paralyze mice at doses 50× the LD_50_ of anabaseine [15]. 

The ecological function of nemertelline, the most abundant pyridine alkaloid, is still unclear as it is only moderately toxic to crustaceans and its anti-fouling activity does not exceed that of 2,3′-BP. Since the environment of this hoplonemertean undoubtedly contains many potentially infective micro-organisms, it seems possible that nemertelline and its congeners may have antimicrobial activity. Nemertelline is readily synthesized via two different methods [24,25], so testing for antimicrobial activity should be feasible. 

While 2,3′-BP and 3Me-2,3′-BP have only a mild paralytic activity on mice and a weak interaction with nicotinic acetylcholine receptors, the vertebrate-active components of *Aa* (isoanatabine, anabasine, and anabaseine) have complementary properties relative to the BPs. The first two compounds have one highly basic secondary amine nitrogen, which allows a greater fraction of the molecules to be in the active, ionized form at a physiological pH [26,27]. Anabaseine has a wide spectrum of nAChR activity in vertebrates as well as invertebrates. Interestingly, it was found to be concentrated in the *Aa* median proboscis, which also contains the stylet apparatus (Kem, unpublished results). This suggests that its primary function is the paralysis of prey. As might be expected, isoanatabine and anatabine have very similar pharmacological properties and are relatively potent toxins to vertebrates. The two enantiomers of isoanatabine also exhibit similar pharmacolgical profiles on the two most abundant mammalian brain nicotinic receptors [28]. In the present investigation, we measured the circular dichroism spectrum of natural isoanatabine but failed to find optical activity, a measure of the prevalence of one enantiomer over the other.

The occurrence of a dihydroisoquinoline alkaloid like angulatine in an animal is unusual. While plants make a wide variety of pharmacologically active isoquinolines, few of these compounds have been isolated from marine animals. Imbricatine, a sea star tetrahydroisoquinoline, may be the first marine compound of this type that has been reported [29]. There are also isoquinoline compounds in certain sponges, aptamines, that have been found to exert a variety of pharmacological actions on the nervous system, as well as having anti-cancer activities [30,31,32]. Angulatine has unusual substituents on the aromatic portion of its dihydroisoquinoline ring, making it rather unique and difficult to synthesize using traditional methods. Our attempts to synthesize angulatine have not been successful, probably because the 6- and 7-position substituents are not sufficiently electron-donating to facilitate the final ring-closing step (a Pictet–Spengler reaction). We had insufficient amounts of this natural product to assess its toxicity beyond showing that it potently paralyzes crayfish, which are usually quite sensitive to compounds like nicotine and anabaseine with cholinergic system activity. A compound containing three of the four rings in angulatine was synthesized but, apparently, was not pharmacologically tested [33].

### 3.2. Possible Pathways for Hoplonemertean Alkaloid Biosynthesis

**Figure 8:**
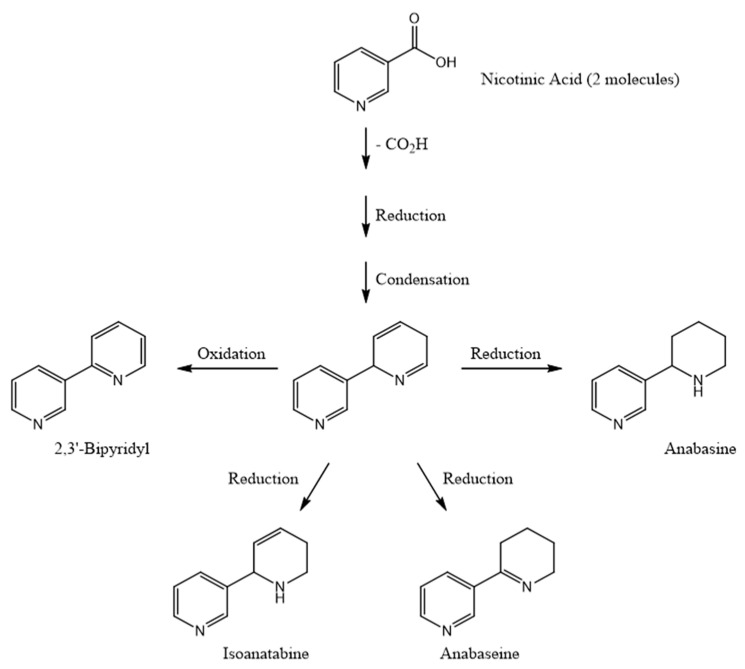
Possible pathway for biosynthesis of nemertean pyridyl alkaloids starting with nicotinic acid.

**Figure 9:**
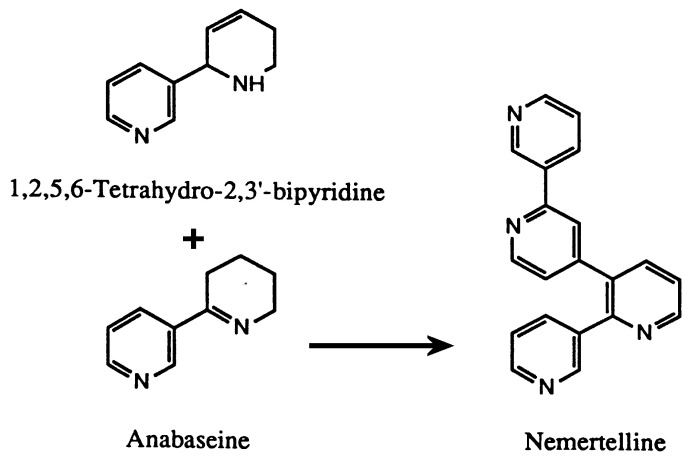
Possible biosynthetic pathway for nemertelline. An activated form of isoanatabine precursor could react with the electronegative carbon at position 3 of the tetrahydropyridyl ring of anabaseine.

Anabasine is the dominant alkaloid in certain tobacco plant species. In fact, the alkaloid’s name comes from the tobacco genus *Anabasis*. As previously mentioned, several species of hoplonemerteans and ants also contain significant amounts of anabasine as well as anabaseine. Leete [34] extensively investigated the biosynthetic pathways for anabasine, anatabine, and nicotine in various tobacco species. The 3′-pyridyl rings in nicotine and anabasine were found to derive from nicotinic acid. The sources of the pyrrolidinyl and tetrahydropyridyl rings in these two compounds were, respectively, N-methyl putrescine and lysine. According to Leete [35], both rings of anatabine may originate from lysine. Our speculation concerning the biosynthesis of the *Aa* pyridyl alkaloids is partly based on these plant studies and the observation that the peripheral 3-substituted pyridyl rings in all known hoplonemertean alkaloids are always free of other substituents. We suggest (Figure 8) that the hoplonemertean 3-pyridyl rings originate from nicotinic acid or nicotinamide and that the 2- substituted rings in the bipyridyls and the internal pyridyl rings in nemertellines are derived either from another nicotinate or lysine or from the sequential attachment of short chain fatty acids such as acetate, malonate, or propionate to an activated nicotinic acid (thioester?) moiety. Experiments with labelled potential precursors may be useful in delineating the biosynthetic pathway for these hoplonemertean alkaloids. The biosynthesis of nemertelline and its congeners seems very likely to result from a hetero-dimerization of anabaseine (providing the A and B rings of nemertelline) with an activated precursor of isoanatabine (providing the C and D rings of the tetrapyridyl, as shown in Figure 9). The postulated 3′’-methyl-nemertelline and 3′’-hydroxy-nemertelline (see Figure 1 for the numbering of ring atoms) could be biosynthesized via the reaction of 2-(3′-pyridyl)-3-methyl-1,2,5,6-tetrahydropyridyl (MW 174) or 2-(3′-pyridyl)-3-hydroxy-1,2,5,6-tetrahydropyridyl (MW 176) precursors with anabaseine, respectively. Preliminary evidence for the presence of these two postulated BP precursors of nemertelline was obtained during GC-MS and LC-MS analyses of the crude extract basic chloroform phase.

Several papers have been published in recent years reporting transcriptomic investigations into various nemerteans [36,37,38,39]. These investigations only searched for peptidic toxins. Several decades ago, three types (MWs of 20,000, 6000, and 3500) of peptidic toxins were isolated from pilidiophoran (formerly called heteronemertean) species [40,41,42,43]. While hoplonemerteans have only been demonstrated to contain alkaloid toxins, the transcriptomic data suggested the presence of other peptidic toxins in this group. These inferences need to be substantiated by isolating and pharmacologically characterizing the putative toxins. Hopefully, future “ohmics” studies on hoplonemerteans will also search for the genes of enzymes that might be involved in the biosynthesis of the various hoplonemertean pyridyl alkaloids. Enzymic candidates include lysine decarboxylases, cytochrome P450s, imine reductases, methyl transferases, and enzymes associated with fatty acid oxidation. 

The delineation of the pyridyl biosynthetic pathway(s) would not only shed light on the evolution of this toxin family but could also ultimately provide a means to biosynthesize artificial, as well as natural, pyridine alkaloids of pharmacological interest that might be difficult to obtain from nature in sufficient quantities for pharmacological investigations. Finally, it will also be interesting to determine whether the alkaloid biosynthesis is performed by worm enzymes or by enzymes produced by bacterial endo-symbionts.

## 4. Concluding Remarks

The presence of a relatively large number (probably >15) of pyridyl compounds in *Aa* is a strong indication that at least some species in the class Hoplonemertea contain a remarkable chemical diversity of pyridine alkaloids, perhaps exceeding the alkaloid diversity previously observed in tobacco plants. Other pyridine alkaloids undoubtedly occur in *Aa*, but their NMR spectra could not be obtained with the limited amounts available. These include an MW 174 compound, possibly a 3-methyltetrahydropyridyl ring precursor of 3-methyl-2,3′-BP. Also, a compound was detected that may be an angulatine analog that lacks one methylene group within the 7-ethoxy-methylene sidechain. We will attempt to identify these and other interesting compounds in the future if more chevron nemerteans can be collected. The identification of additional alkaloids in this and other hoplonemerteans could assist in suggesting possible biosynthetic relationships between the compounds, as well as provide compounds with novel pharmacological activities. 

## 5. Materials and Methods 

### 5.1. Animal Collection

Altogether, two hundred (175 g fresh weight) *A. angulatus* worms were collected at exceptionally low tides along the Maine side of Passamaquoddy Bay, near Eastport, Maine. The live worms were initially placed in polyethylene bottles containing approximately 10 volumes (per fresh weight) of ethanol containing 1% acetic acid. The bottles were transported to Florida at ambient temperatures, then stored at −20 °C prior to alkaloid analyses. 

### 5.2. Alkaloid Extraction and Purification

After removing the acidic ethanol preservative, the preserved worms were extracted with an additional 0.8 L of acidic ethanol, yielding a cumulative volume of 2.6 L acidic ethanol solution. This was filtered and evaporated under vacuum at room temperature, yielding a syrup. To this partially particulate residue (25.2 g), 60 mL water and 120 mL chloroform were added. The pH was then brought to 1.5 with concentrated hydrochloric acid. This was hand-shaken in a separatory funnel. After standing overnight at room temperature, the two phases were separated and the acidic aqueous phase was again extracted with 2 × 120 mL chloroform. The pH of the separated aqueous phase was brought to 12 with ice-cold 40% sodium hydroxide, and then 100 mL dichloromethane was added to extract the basic compounds. After shaking and standing, the resulting interfacial suspension was broken down via centrifugation and the acidic aqueous phase was re-extracted with 5 × 100 mL dichloromethane. The combined basic organic solutions were dried over magnesium sulfate and rotary-evaporated under vacuum at 20 °C, yielding 0.56 g of oily crude alkaloid extract. Column chromatography using 50 g of silica gel with ether-diethylamine (9:1, *v*/*v*) development provided several fractions: 67 mg of crude 2,3′-BP fraction, 12 mg of an intermediate fraction, 206 mg of a crude nemertelline fraction, and 241 mg of the most polar alkaloids. The 2,3′-BP fraction was further purified via column chromatography on 20 g silica gel developed with cyclohexane-diethylamine (8:2, *v*/*v*), giving 32 mg of purified 2,3′-bipyridyl, which was examined for its methyl-2,3′-bipyridine content. 

### 5.3. Gas Chromatographic Analysis of the Crude Alkaloid Fraction 

To resolve and identify the different *Aa* alkaloids, GC-EIMS measurements utilized a 3 m length 0.25 mm i.d. 5% phenylmethylpolysiloxane (DB-5MS, J & W Scientific, Folsom, CA, USA) capillary column with oven temp of 300 °C.

### 5.4. LC-MS Analyses 

A reverse-phase HPLC-(+)ESI-MS analysis of the chloroform extracted compounds was performed on a Thermo Fisher Scientific (Waltham, MA, USA) LTQ classic quadrupole ion trap mass spectrometer operated in the positive electrospray ionization (ESI) mode. ESI-normal MS scans were taken, and data-dependent MS/MS scans were obtained on the most abundant ion of the preceding MS scan with a 4u-isolation window, 0.3 qCID, 42.5% normalized CID, and 30ms CID duration. Chromatography was performed with an Agilent (Santa Clara, CA, USA) 1100 series binary pump and a Waters (Milford, MA, USA) Xterra MS C18 (2.1 mm × 150 mm; 3.5 μm) with Phenomenex (Torrance, CA, USA) C18 Security Guard Column (2 mm × 4 mm). Mobile phase A was water plus 0.2% acetic acid, and mobile phase B was methanol with 0.2% acetic acid. With a flow rate of 0.15 mL/min, the gradient was 0%B (0 min) to 30%B at 15 min then to 95%B at 60 min and held for 20 min. An Agilent (Santa Clara, CA, USA) 1100 G1314A UV/Vis detector was positioned between the HPLC column and MS and monitored the HPLC effluent at 254 nm.

The time-of-flight high resolution mass spectrometry (TOF-HRMS) was performed on an Agilent (Santa Clara, CA, USA) 6220A time-of-flight HRMS interfaced to an Agilent 1100 binary HPLC. Flow injection analysis was used to introduce the sample. The TOF-HRMS was operated in the (+)ESI mode. Elemental composition of the DMAB-natural product was calculated with the Xcalibur Qual Browser Elemental Composition tool, Qual Browser, Thermo Fisher Scientific (Waltham, MA, USA) Xcalibur 2.2 SP1.48. CI-HRMS and FAB-HRMS analyses were performed by Cris Dancel.

### 5.5. NMR Spectroscopy

NMR spectra were acquired in CDCl_3_ on Varian Unity 300 MHz and Bruker Avance 500, 600, and 800 MHz spectrometers. The 600 MHz instrument was equipped with a unique 1-mm CryoProbe. Chemical shift axes in both proton and carbon spectra were referenced to internal tetramethylsilane at 0.0 ppm. The COSY (Correlation SpectroscopY), HSQC (Heteronuclear Single Quantum Coherence, with ^1^J_CH_ set to 145 Hz), and HMBC (Heteronuclear Multiple Bond Correlation, with ^n^J_CH_ set to 8 Hz) spectra were acquired using conventional methods. NMR data for nemertelline was obtained on a sub-milligram quantity of the natural substance in a 3 mm tube with 180 µl of CDCl_3_ solution using the 800 MHz instrument’s 5 mm TCI CryoProbe. Spectra and spectral data of the five new compounds are to be found in the Supplementary Materials section.

## Figures and Tables

**Table 1:** Comparison of the GC-EI-MS peak 2 compound with synthetic 1,2,5,6-tetrahydro-2,3′-bipyridyl (isoanatabine) and anatabine. Fragment amplitude is expressed as percent of base peak. M^+.^ is abbreviated to M.

*m*/*z*	*Aa* Natural Product	Isoanatabine	Anatabine	Anabaseine
160 M	17 (C_10_H_12_N_2_) ^1^	15	100	100
159 M-1	41 C_10_H_11_N_2_)	39		73
145 M-15	44	52	19	23
132 M-28	---	---	45	---
131 M-29	19 (C_9_H_9_N)	33	46	38
130 M-30	78 (C_9_H_8_N)	98	15	---
118 M-42	10	---	19	---
117 M-43	13	---	---	---
105 M-55	17 (C_7_H_7_N)	26	17	25
104 M-56	17 (C_7_H_6_N)	23	22	38
103 M-57	18	15	---	---
89 M-71	14	---	---	---
82 M-78	100 (C_5_H_8_N)	100	34	---
80 M-80	83 (C_5_H_6_N)	69	28	---
79 M-81	11	11	31	---
78 M-82	41 (C_5_H_4_N)	50	25	11
77 M-93	43	23	13	13
76 M-94	15	---	13	---

^1^ Elemental compositions of the molecular ions and selected fragments were determined by HR-EIMS.

**Table 2:** Comparison of the GC-EI-MS spectra of the four *Aa* nemertellines. The molecular ion M^+.^ is abbreviated as M. Fragment amplitude is expressed as percent of base peak. Dashes indicate a fragment abundance relative to base peak of 0–10%. The elemental compositions of all four compounds were consistent with their names. Ions generated via a common mass loss are highlighted in red.

*m*/*z*	Nemertelline	Tetrahydro- Nemertelline	Methyl- Nemertelline	Hydroxy- Nemertelline
326				56 ^1^ M
325				100 M-1
324			67 M	---
323			100 M-1	---
314		74 M	---	---
313		47 M-1	---	---
310	37 M	---	---	15 M-16
309	100 M-1	---	---	48 M-17
298	---	---		10 ^1^ M-28
296	---	---	10 M-28	---
294	---	---	16 M-30	---
282	13 M-28	---	---	---
281	10 M-29	---	---	---
271	---	18 M-43	---	---
270	---	22 M-44	---	---
248	---	---	---	13 ^1^ M-78
246	---	---	52 M-78	---
236	---	7 M-78	---	---
232	7 M-78	---	---	---
221	---	---	---	12 M-105
219	---	---	22 M-105	---
210	---	15 M-104	---	---
209	---	43 M-105	---	16 ^1^ M-116
206	9 M-104	---	---	---
205	36 M-105	---	---	---
197	---	---	---	42 ^1^ M-129
195	---	22 M-119	---	---
194	---	20 M-120	---	14 M-132
183	---	28 M-131	---	22 ^1^ M-143
182	---	22 M-132	---	---
181	---	100 M-133	---	---
169	---	---	18 M-155	---
168	---	20 M-146	---	16 M-158
167	---	11 M-147	---	---
166	---	12 M-148	---	---
159	---	23 M-155	---	---
156	5 M-155	10 M-158	---	---
155	---	10 M-159	---	---

^1^ These fragments were shown to contain one atom of oxygen byHR-EI-MS.

## Data Availability

Additional NMR and MS data for identified compounds will be made available upon request.

## Supplementary Materials

The following supporting information can be downloaded at: https://www.mdpi.com/article/10.3390/md22040141/s1, See pages S1–S37 for NMR data on the five new compounds and nemertelline.
