# Supplementary material for: New Pyridyl and Dihydroisoquinoline Alkaloids Isolated from the Chevron Nemertean Amphiporus angulatus
Source: Mar Drugs. 2024 Mar 22;22(4):141. doi: 10.3390/md22040141 (PMC11050936; doi:10.3390/md22040141)
Supplement: Supplementary file 1 [file marinedrugs-22-00141-s001.zip › marinedrugs-2876590-supplementary.pdf]

Isoanatabine (Nat. Product) PMR

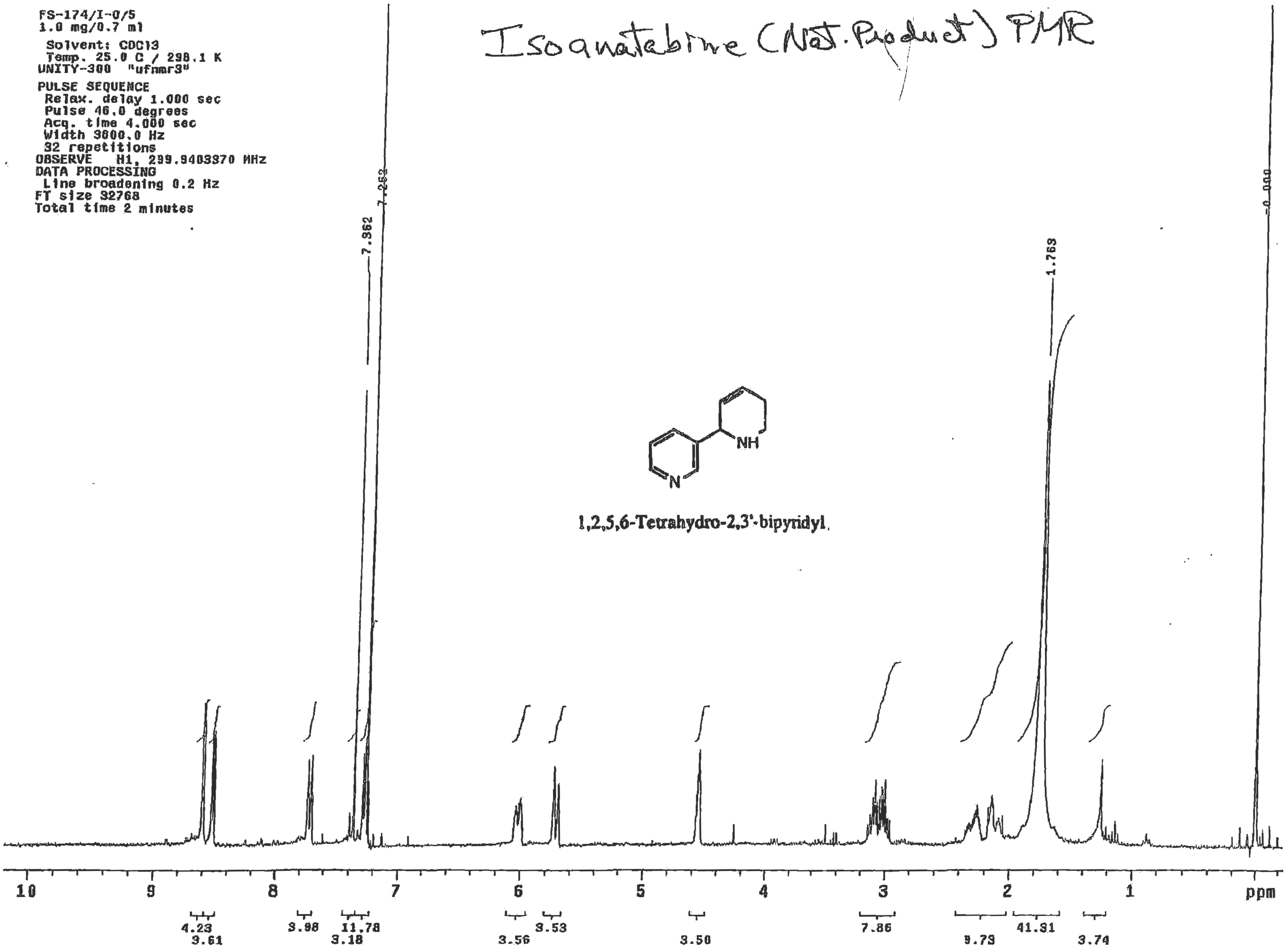

S1

Angulatine proton NMR spectrum

Ferenc Soti/William Kem, FS-174-NEW-A
2.4 mg in about 0.165 ml CDCl3 (99.8%)
40 mm Solution in a Wilmad 307-PP Tube
5 mm BB Probe, Tuned at 25C, Spin=20Hz
Varian Unity-300 NMR Spectrometer
The Center for Structural Biology
University of Florida, Gainesville
CSB #187, Operator: Jim Rocca

Alkaloid of *Amphiporus angulatus*

1,6-Bis(pyrid-3-yl)-7-ethoxymethyl-3,4-dihydroisoquinoline

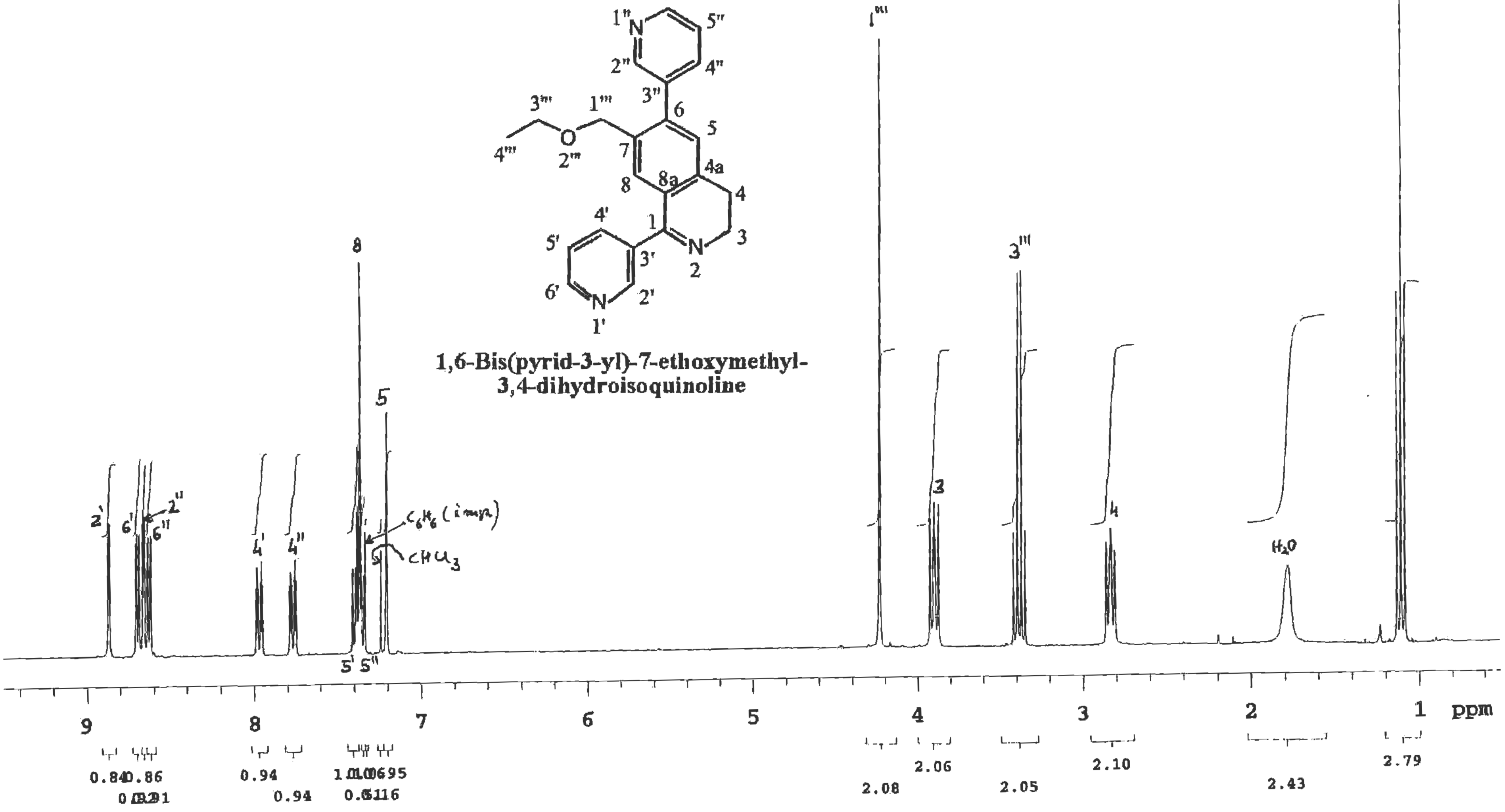

S2

Angulatine

Table S1. Proton NMR Data for Angulatine in $CDCl_3$

[1]H, δ (ppm) [a]	Multiplicity; J's (Hz)	Affects of Decoupling	NOE's (% Enhancement) [b]	Assignment
8.87	dd; 0.8, 2.3			HC(2'); 3'-pyridyl
8.70	dd; 1.8, 4.8			HC(6'); 3'-pyridyl
8.66	dd; 0.9, 2.4			HC(2"); 3"-pyridyl
8.63	dd; 1.8, 4.8			HC(6"); 3"-pyridyl
7.97	ddd; 1.8, 2.2, 7.9	8.87, 8.70, 7.39		HC(4'); 3'-pyridyl
7.77	ddd; 1.8, 2.2, 7.9	8.66, 8.63, 7.36		HC(4"); 3"-pyridyl
7.39	ddd; 0.9, 4.8, 7.8			HC(5'); 3'-pyridyl
7.37	s; broadened		4.22 (5%)	HC; aryl
7.36	ddd; 0.9, 4.8, 7.8			HC(5"); 3"-pyridyl
7.21	s; broadened		2.84 (4%)	HC; aryl
4.22	s; sharp		8.66 (3%), 7.77 (3%), 7.37 (11%)	-O-CH_2-Ar
3.90	m; non-first-order (approximate t; 7.3)	2.84 → s		=N-CH_2-CH_2-R
3.38	q; 7.0	1.11 → s		-O-CH_2-CH_3
2.84	m; non-first-order (approximate t; 7.3)	3.90 → s	7.21 (15%)	=N-CH_2-CH_2-R
1.11	t; 7.0	3.38 → s		-O-CH_2-CH_3

[a] At 300 MHz. Chemical shifts are reported relative to residual [1]$HCCl_3$, which was assigned a shift of 7.26 ppm.

[b] NOE's were measured from difference experiments and are reported as percent enhancement of the affected resonance relative to the saturated resonance, which was assigned a value of –100% per proton. E.g., saturating δ= 7.21 (-100%) enhanced δ= 2.84 (+4%), and saturating δ= 2.84 (-200%) enhanced δ= 7.21 (+15%)

Angulatine ^{13}C proton decoupled Spectrum

Ferenc Soti/William Kem, FS-174-NEW-A
2.4 mg in about 0.145 ml CDC13 (99.8%)
35 mm Solution in a Wilmad 307-PP Tube
5 mm BB Probe, Tuned at RT, Spin=20Hz
Varian Unity-300 NMR Spectrometer
The Center for Structural Biology
Advanced Magnetic Resonance Imaging
& Spectroscopy, U F Brain Institute
University of Florida, Gainesville
CSB # 213, Op: Jim Rocca, AMRIS

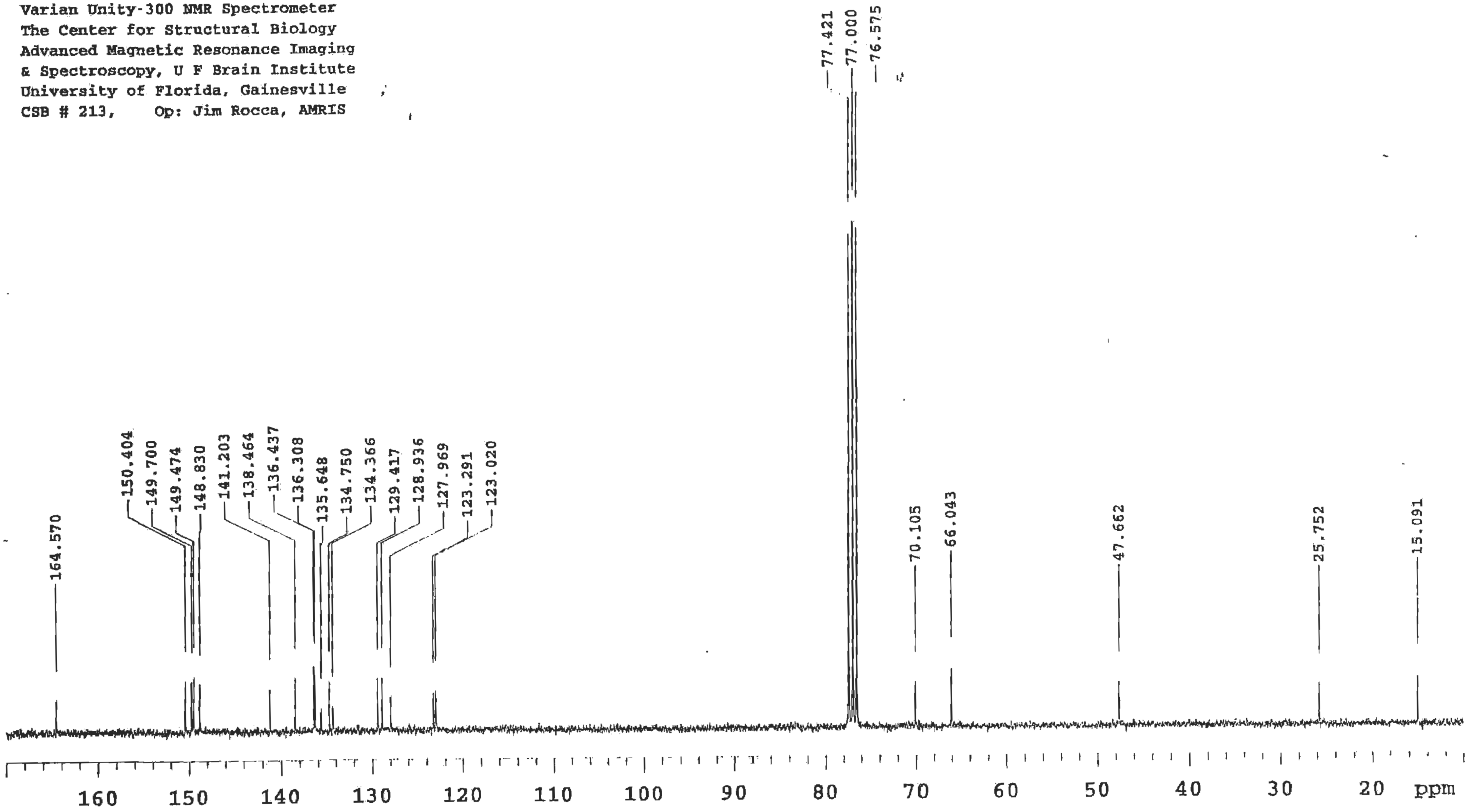

Angulatine

Table S2. Carbon NMR Data and Carbon-Proton Correlations for Angulatin in $CDCl_3$.

^{13}C, δ (ppm) [a]	Multiplicity, $^1J_{CH}$ (Hz)	^1H, δ (ppm) [a, b]	Multiple Bond ^1H's, δ (ppm) [c]	Assignment
164.57	s		7.97, 7.37 ?, 3.90	N=C(1)
150.40	d, ???	8.70	7.97	HC(6'); 3'-pyridyl
149.70	d, 181	8.87		HC(2'); 3'-pyridyl
149.47	d, 178	8.66		HC(2"); 3"-pyridyl
148.83	d, ???	8.63	7.77	HC(6"); 3"-pyridyl
141.20	s		7.77, 7.37 ?, 4.22	
138.46	s		7.37 ?, 3.90, 2.84	
136.44	d, 162	7.77	8.63	HC(4"); 3"-pyridyl
136. 31	d, 163	7.97	8.87, 8.70	HC(4'); 3'-pyridyl
135.65	s		8.66 ?, 7.37 ?, 7.36 ?	
134.75	s		7.21, 4.22 ?	
134.37	s		8.87, 4.22 ?, 3.90	
129.42	d, 159	7.21	2.84	HC; aryl
128.94	d, 159	7.37	4.22	HC; aryl
127.97	s		7.21 ? (vs. benzene), 3.90, 2.84,	
123.29	d, 166	7.39		HC(5'); 3'-pyridyl
123.02	d, 164	7.36		HC(5"); 3"-pyridyl
70.10	t, 141	4.22	7.37, 3.38	-O-H_2C(1''')-Ar
66.04	t, 140.5	3.38	4.22, 1.11	-O-H_2C(3''')-CH_3
47.66	t, 139.5	3.90	2.84	=N-H_2C(3) -CH_2-R
25.75	t, 130	2.84	7.37, 7.21, 3.90	=N-CH_2-H_2C(4)-R
15.09	q, 126	1.11	3.38	-O-CH_2-H_3C(4''')

[a] ^{13}C at 75 MHz and ^1H at 300 or 500 MHz. Chemical shifts are reported relative to $^{13}CDCl_3$ and to residual $^1HCCl_3$,which were assigned shifts of δC= 77.0 ppm and δH= 7.26 ppm.

[b] Direct $^1H/^{13}C$ (500/126 MHz) correlations from a 2D-HMQC experiment.

[c] Indirect $^1H/^{13}C$ (500/126 MHz) correlations from four 2D-HMBC experiments, optimized for couplings of 3, 7, 11 and 20 Hz.

Tetrahydro-Nemertelline Spectral Data

TH Nem-1

HPLC Peak #9, Concentrated from 0.6 ml, About 0.7 mg, 0.18 ml WK's CDCl3 (99.96% D)
William Kem and Ferenc Soti, Department of Pharmacology and Therapeutics, 392-0669, Enc: Kem-002
2.5 mm TXI Probe, Temp= 27 C, Non Spun, Bruker Avance 500 Console, Magnex 11.75 T/54 mm Magnet
Advanced Magnetic Resonance Imaging and Spectroscopy, McKnight Brain Institute, University of Florida
Jim Rocca, AMRIS

PMR
Spectrum p.1

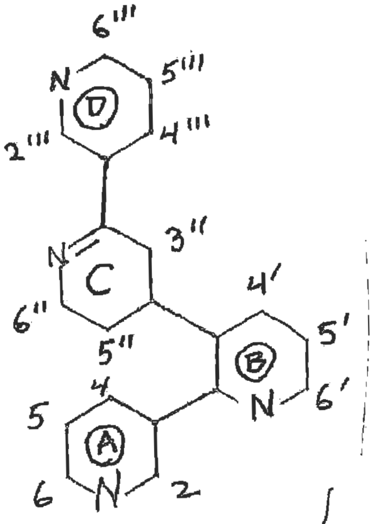

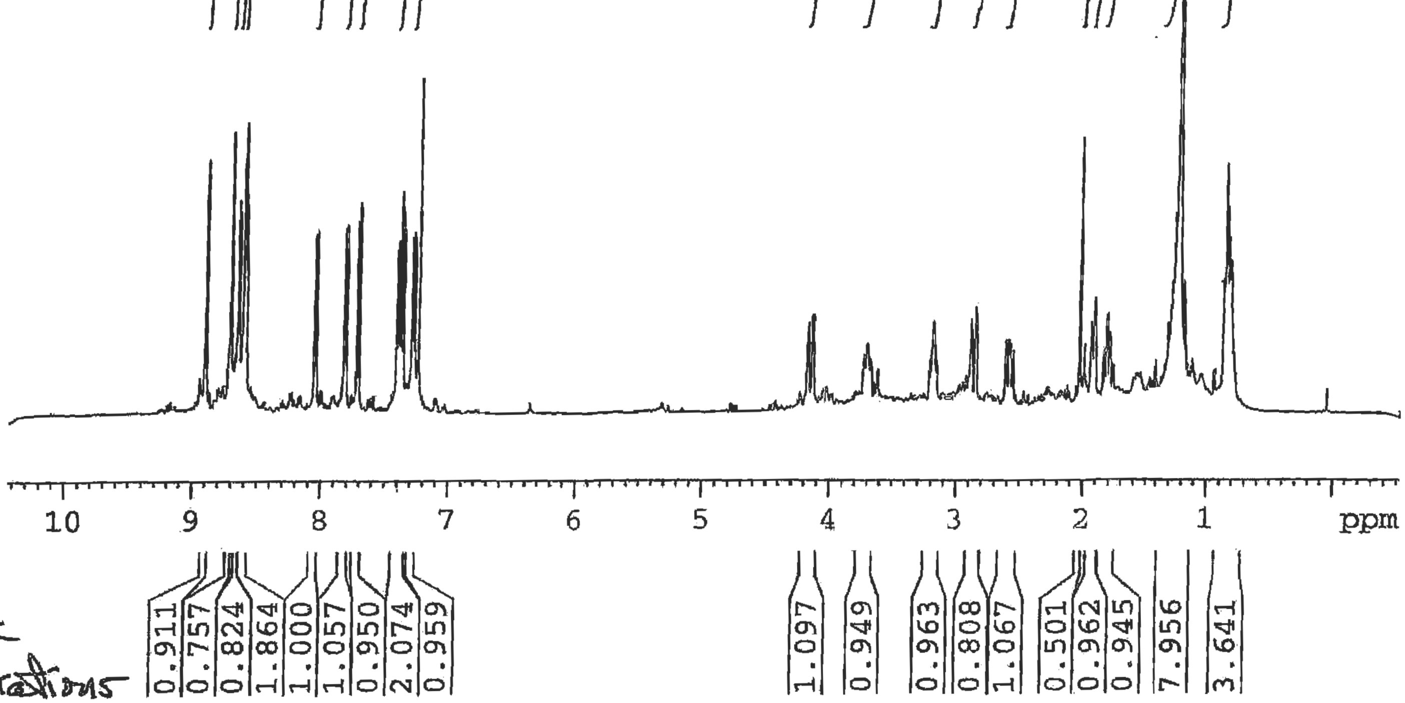

Peak Integrations

Current Data Parameters

NAME	WK_27Jun07_#9_CDCl3
EXPNO	7
PROCNO	1

F2 - Acquisition Parameters

Date_	20070702
Time	13.34
INSTRUM	spect
PROBHD	2.5 mm TXI 1H/
PULPROG	zg
TD	32768
SOLVENT	CDCl3
NS	32
DS	1
SWH	5482.456 Hz
FIDRES	0.167311 Hz
AQ	2.9885828 sec
RG	1024
DW	91.200 usec
DE	6.00 usec
TE	300.0 K
D1	2.00000000 sec
MCREST	0.00000000 sec
MCWRK	0.01500000 sec

======== CHANNEL f1 ========

NUC1	1H
P1	4.00 usec
PL1	6.00 dB
SFO1	500.4025020 MHz

F2 - Processing parameters

SI	32768
SF	500.4000257 MHz
WDW	EM
SSB	0
LB	0.40 Hz
GB	0
PC	0.20

HPLC Peak #9, Concentrated from 0.6 ml, About 0.7 mg, 0.18 ml WK's CDCl3 (99.96% D)
William Kem and Ferenc Soti, Department of Pharmacology and Therapeutics, 392-0669, Enc: Kem-002
2.5 mm TXI Probe, Temp= 27 C, Non Spun, Bruker Avance 500 Console, Magnex 11.75 T/54 mm Magnet
Advanced Magnetic Resonance Imaging and Spectroscopy, McKnight Brain Institute, University of Florida
Jim Rocca, AMRIS

PMR Spectrum p. 2

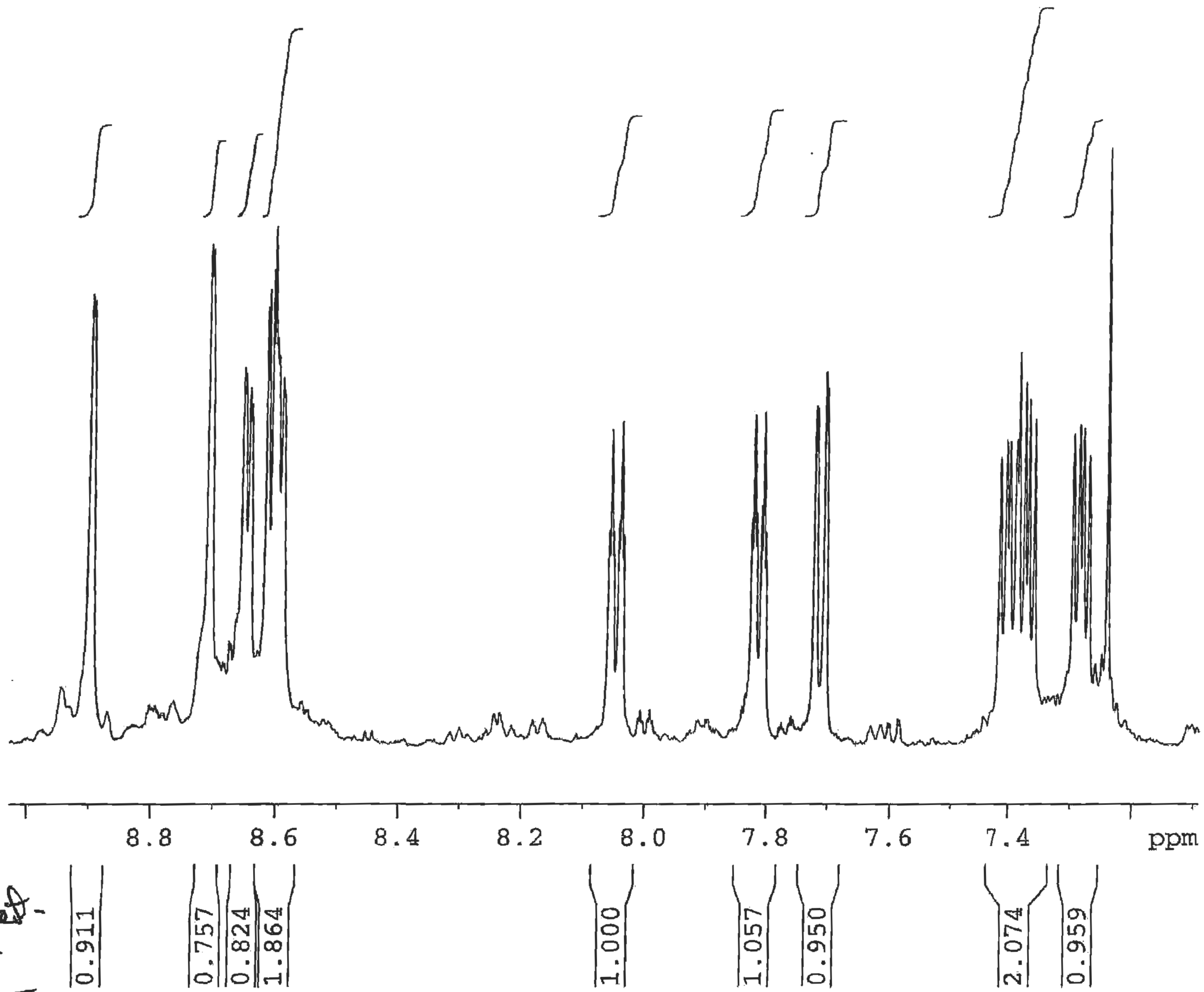

Integration of Peaks

Current Data Parameters

NAME	WK_27Jun07_#9_CDCl3
EXPNO	7
PROCNO	1

F2 - Acquisition Parameters

Date_	20070702
Time	13.34
INSTRUM	spect
PROBHD	2.5 mm TXI 1H/
PULPROG	zg
TD	32768
SOLVENT	CDCl3
NS	32
DS	1
SWH	5482.456 Hz
FIDRES	0.167311 Hz
AQ	2.9885828 sec
RG	1024
DW	91.200 usec
DE	6.00 usec
TE	300.0 K
D1	2.00000000 sec
MCREST	0.00000000 sec
MCWRK	0.01500000 sec

======== CHANNEL f1 ========

NUC1	1H
P1	4.00 usec
PL1	6.00 dB
SFO1	500.4025020 MHz

F2 - Processing parameters

SI	32768
SF	500.4000257 MHz
WDW	EM
SSB	0
LB	0.40 Hz
GB	0
PC	0.20

TH Nem - 3

HPLC Peak #9, Concentrated from 0.6 ml, About 0.7 mg, 0.18 ml WK's CDCl3 (99.96% D)
William Kem and Ferenc Soti, Department of Pharmacology and Therapeutics, 392-0669, Enc: Kem-002
2.5 mm TXI Probe, Temp= 27 C, Non Spun, Bruker Avance 500 Console, Magnex 11.75 T/54 mm Magnet
Advanced Magnetic Resonance Imaging and Spectroscopy, McKnight Brain Institute, University of Florida
Jim Rocca, AMRIS

PMR Spectrum, p. 3

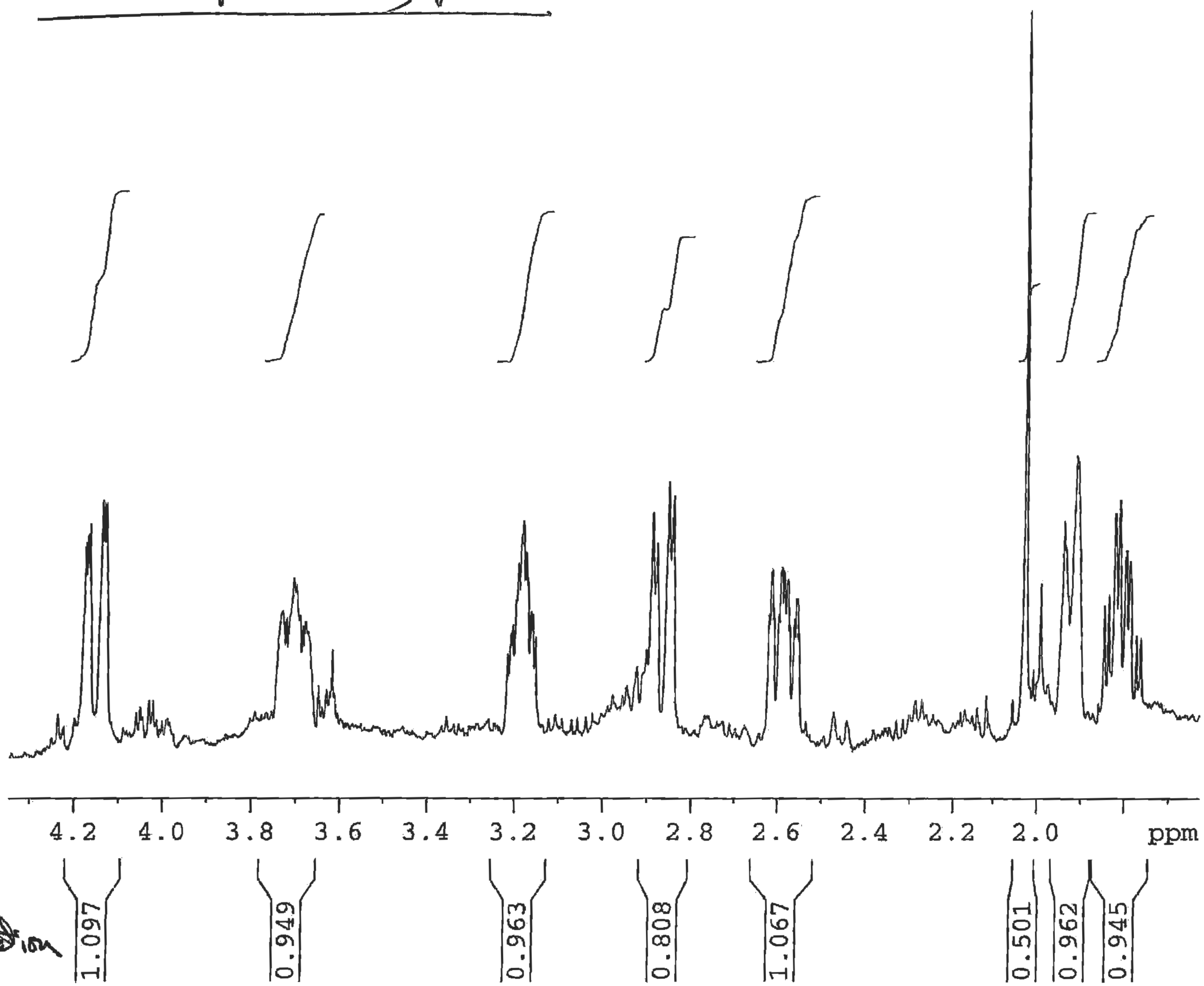

Current Data Parameters

NAME	WK_27Jun07_#9_CDCl3
EXPNO	7
PROCNO	1

F2 - Acquisition Parameters

Date_	20070702
Time	13.34
INSTRUM	spect
PROBHD	2.5 mm TXI 1H/
PULPROG	zg
TD	32768
SOLVENT	CDCl3
NS	32
DS	1
SWH	5482.456 Hz
FIDRES	0.167311 Hz
AQ	2.9885828 sec
RG	1024
DW	91.200 usec
DE	6.00 usec
TE	300.0 K
D1	2.00000000 sec
MCREST	0.00000000 sec
MCWRK	0.01500000 sec

======== CHANNEL f1 ========

NUC1	1H
P1	4.00 usec
PL1	6.00 dB
SFO1	500.4025020 MHz

F2 - Processing parameters

SI	32768
SF	500.4000257 MHz
WDW	EM
SSB	0
LB	0.40 Hz
GB	0
PC	0.20

HPLC Peak #9, Concentrated from 0.6 ml, About 0.7 mg, 0.18 ml WK's CDCl3 (99.96% D)
William Kem and Ferenc Soti, Department of Pharmacology and Therapeutics, 392-0669, Enc: Kem-002
2.5 mm TXI Probe, Temp= 27 C, Non Spun, Bruker Avance 500 Console, Magnex 11.75 T/54 mm Magnet
Advanced Magnetic Resonance Imaging and Spectroscopy, McKnight Brain Institute, University of Florida
Jim Rocca, AMRIS

PMR Spectrum
p. 4

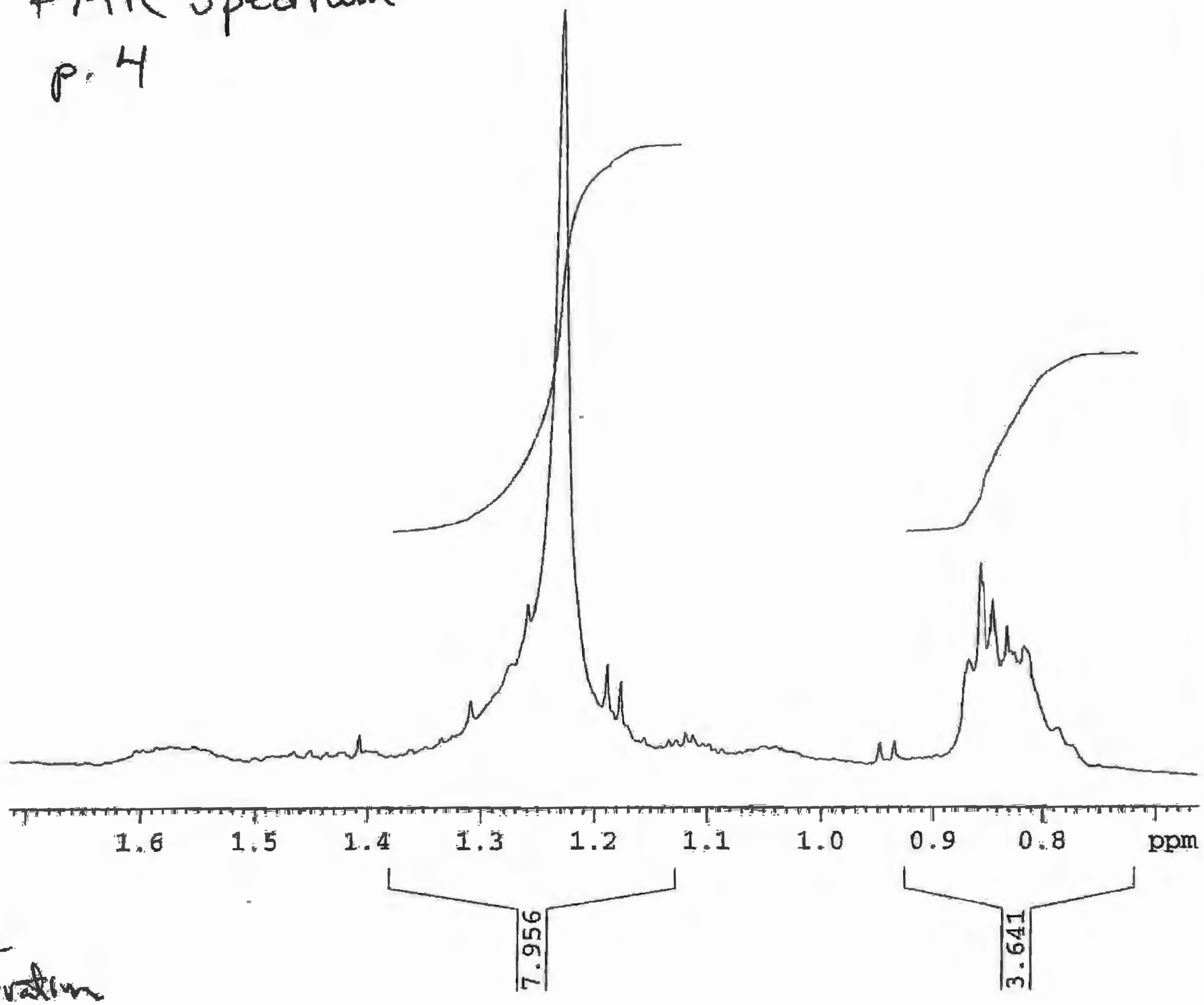

Peak Integration

Current Data Parameters

NAME	WK_27Jun07_#9_CDCl3
EXPNO	7
PROCNO	1

F2 - Acquisition Parameters

Date_	20070702
Time	13.34
INSTRUM	spect
PROBHD	2.5 mm TXI 1H/
PULPROG	zg
TD	32768
SOLVENT	CDCl3
NS	32
DS	1
SWH	5482.456 Hz
FIDRES	0.167311 Hz
AQ	2.9885828 sec
RG	1024
DW	91.200 usec
DE	6.00 usec
TE	300.0 K
D1	2.00000000 sec
MCREST	0.00000000 sec
MCWRK	0.01500000 sec

======== CHANNEL f1 ========

NUC1	1H
P1	4.00 usec
PL1	6.00 dB
SFO1	500.4025020 MHz

F2 - Processing parameters

SI	32768
SF	500.4000257 MHz
WDW	EM
SSB	0
LB	0.40 Hz
GB	0
PC	0.20

COSY

TH Nem-5
A

HPLC Peak #9, Concentrated from 0.6 ml, About 0.7 mg, 0.18 ml WK's CDCl3 (99.96% D)
William Kem and Ferenc Soti, Department of Pharmacology and Therapeutics, 392-0669, Enc: Kem-002
2.5 mm TXI Probe, Temp= 27 C, Non Spun, Bruker Avance 500 Console, Magnex 11.75 T/54 mm Magnet
Advanced Magnetic Resonance Imaging and Spectroscopy, McKnight Brain Institute, University of Florida
Jim Rocca, AMRIS

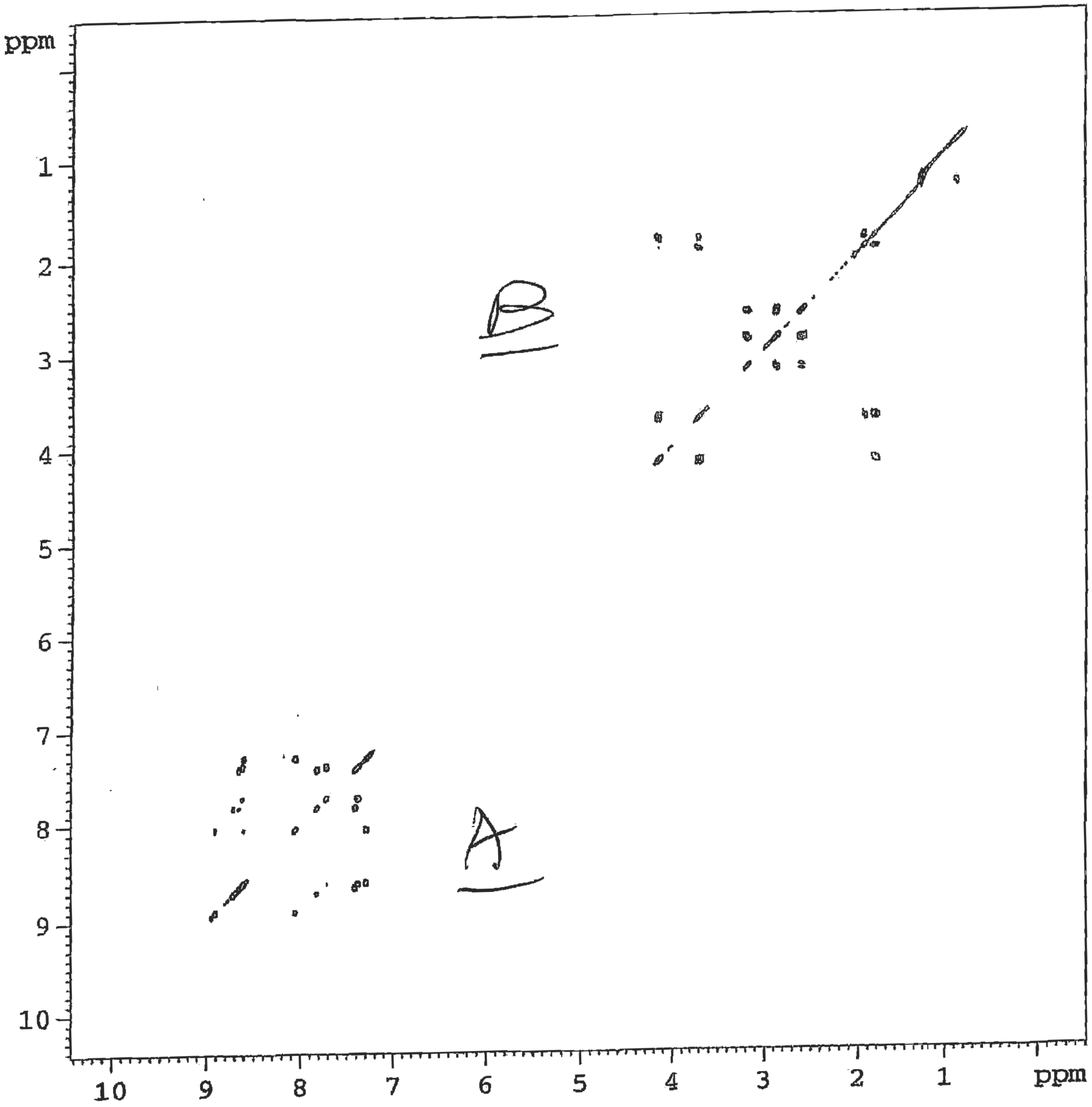

Current Data Parameters

NAME	WK_27Jun07_#9_CDCl3
EXPNO	4
PROCNO	1

F2 - Acquisition Parameters

Date_	20070630
Time	9.55
INSTRUM	spect
PROBHD	2.5 mm TXI 1H/
PULPROG	cosygpqf
TD	2048
SOLVENT	CDCl3
NS	8
DS	4
SWH	5482.456 Hz
FIDRES	2.676980 Hz
AQ	0.1869188 sec
RG	1024
DW	91.200 usec
DE	6.00 usec
TE	300.0 K
d0	0.00000300 sec
D1	2.00000000 sec
d13	0.00000400 sec
D16	0.00020000 sec
IN0	0.00018240 sec
MCREST	0.00000000 sec
MCWRK	2.00000000 sec

======== CHANNEL f1 ========

NUC1	1H
P0	4.00 usec
P1	8.00 usec
PL1	6.00 dB
SFO1	500.4025020 MHz

====== GRADIENT CHANNEL =====

GPNAM1	SINE.100
GPNAM2	SINE.100
GPX1	10.00 %
GPX2	10.00 %
GPY1	10.00 %
GPY2	10.00 %
GPZ1	10.00 %
GPZ2	10.00 %
P16	1000.00 usec

F1 - Acquisition parameters

ND0	1
TD	512
SFO1	500.4025 MHz
FIDRES	10.707922 Hz
SW	10.956 ppm
FnMODE	QF

F2 - Processing parameters

SI	1024
SF	500.4000235 MHz
WDW	SINE
SSB	0
LB	0.00 Hz
GB	0
PC	1.00

F1 - Processing parameters

SI	1024
MC2	QF
SF	500.4000235 MHz
WDW	SINE
SSB	0
LB	0.00 Hz
GB	0

COSY A THNam -5A

HPLC Peak #9, Concentrated from 0.6 ml, About 0.7 mg, 0.18 ml WK's CDCl3 (99.96% D)
William Kem and Ferenc Soti, Department of Pharmacology and Therapeutics, 392-0669, Enc: Kem-002
2.5 mm TXI Probe, Temp= 27 C, Non Spun, Bruker Avance 500 Console, Magnex 11.75 T/54 mm Magnet
Advanced Magnetic Resonance Imaging and Spectroscopy, McKnight Brain Institute, University of Florida
Jim Rocca, AMRIS

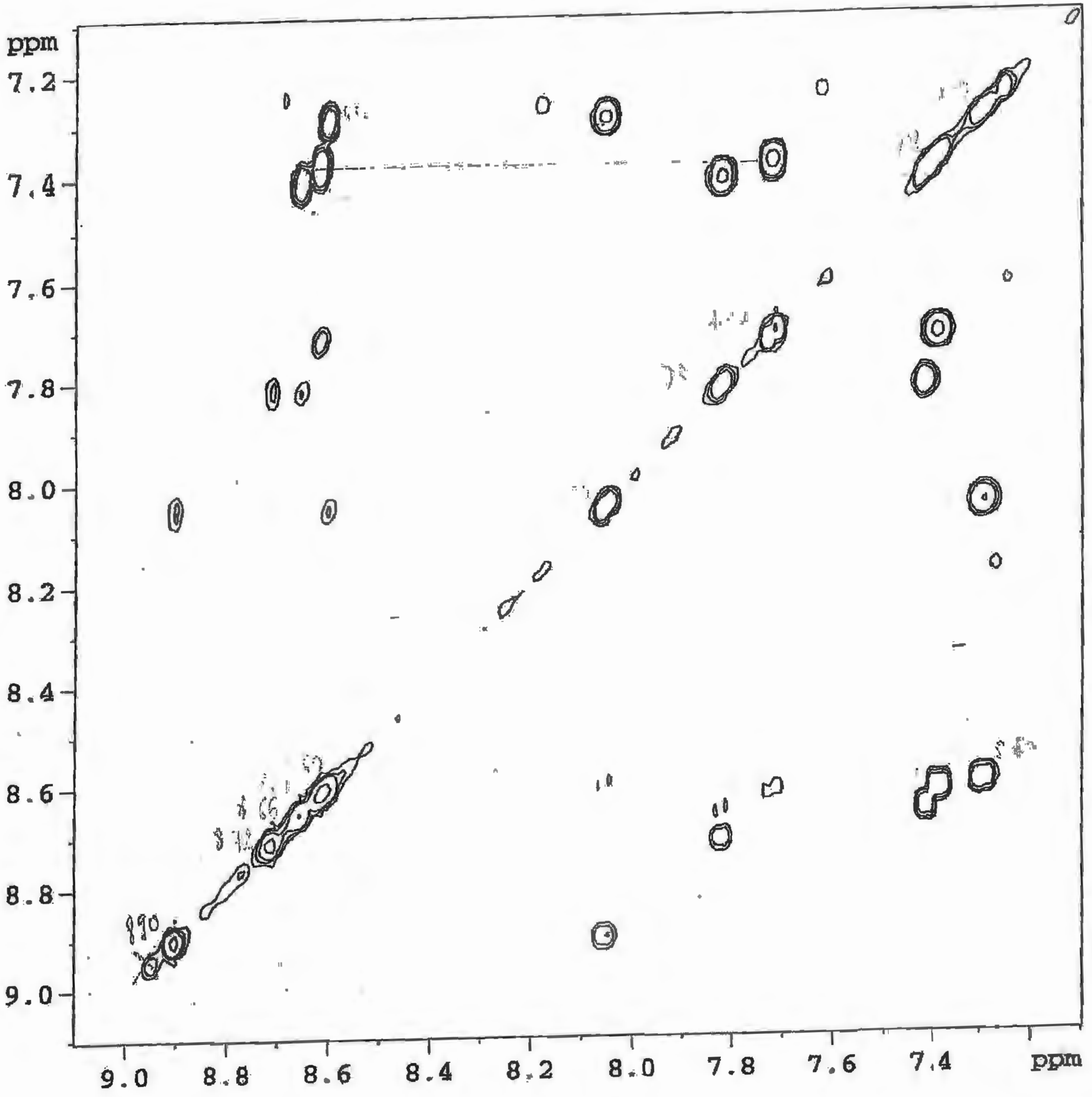

Current Data Parameters

NAME	WK_27Jun07_#9_CDCl3
EXPNO	4
PROCNO	1

F2 - Acquisition Parameters

Date_	20070630
Time	9.55
INSTRUM	spect
PROBHD	2.5 mm TXI 1H/
PULPROG	cosygpqf
TD	2048
SOLVENT	CDCl3
NS	8
DS	4
SWH	5482.456 Hz
FIDRES	2.676980 Hz
AQ	0.1869188 sec
RG	1024
DW	91.200 usec
DE	6.00 usec
TE	300.0 K
d0	0.00000300 sec
D1	2.00000000 sec
d13	0.00000400 sec
D16	0.00020000 sec
IN0	0.00018240 sec
MCREST	0.00000000 sec
MCWRK	2.00000000 sec

======== CHANNEL f1 ========

NUC1	1H
P0	4.00 usec
P1	8.00 usec
PL1	6.00 dB
SFO1	500.4025020 MHz

====== GRADIENT CHANNEL =====

GPNAM1	SINE.100
GPNAM2	SINE.100
GPX1	10.00 %
GPX2	10.00 %
GPY1	10.00 %
GPY2	10.00 %
GPZ1	10.00 %
GPZ2	10.00 %
P16	1000.00 usec

F1 - Acquisition parameters

ND0	1
TD	512
SFO1	500.4025 MHz
FIDRES	10.707922 Hz
SW	10.956 ppm
FnMODE	QF

F2 - Processing parameters

SI	1024
SF	500.4000235 MHz
WDW	SINE
SSB	0
LB	0.00 Hz
GB	0
PC	1.00

F1 - Processing parameters

SI	1024
MC2	QF
SF	500.4000235 MHz
WDW	SINE
SSB	0
LB	0.00 Hz
GB	0

COSY B
TH New -5B

HPLC Peak #9, Concentrated from 0.6 ml, About 0.7 mg, 0.18 ml WK's CDCl3 (99.96% D)
William Kem and Ferenc Soti, Department of Pharmacology and Therapeutics, 392-0669, Enc: Kem-002
2.5 mm TXI Probe, Temp= 27 C, Non Spun, Bruker Avance 500 Console, Magnex 11.75 T/54 mm Magnet
Advanced Magnetic Resonance Imaging and Spectroscopy, McKnight Brain Institute, University of Florida
Jim Rocca, AMRIS

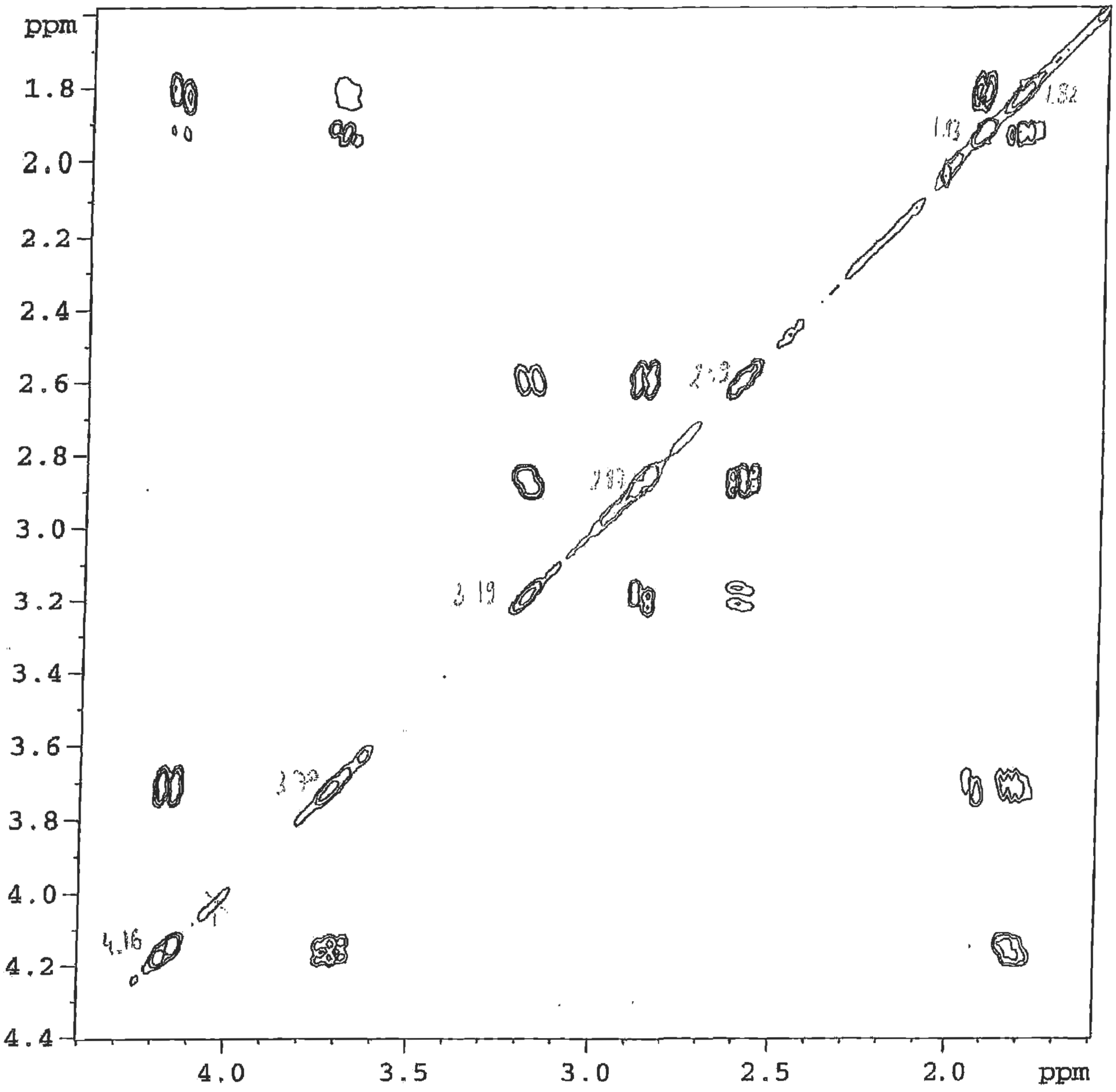

Current Data Parameters
NAME WK_27Jun07_#9_CDCl3
EXPNO 4
PROCNO 1

F2 - Acquisition Parameters
Date_ 20070630
Time 9.55
INSTRUM spect
PROBHD 2.5 mm TXI 1H/
PULPROG cosygpqf
TD 2048
SOLVENT CDCl3
NS 8
DS 4
SWH 5482.456 Hz
FIDRES 2.676980 Hz
AQ 0.1869188 sec
RG 1024
DW 91.200 usec
DE 6.00 usec
TE 300.0 K
d0 0.00000300 sec
D1 2.00000000 sec
d13 0.00000400 sec
D16 0.00020000 sec
IN0 0.00018240 sec
MCREST 0.00000000 sec
MCWRK 2.00000000 sec

======== CHANNEL f1 ========
NUC1 1H
P0 4.00 usec
P1 8.00 usec
PL1 6.00 dB
SFO1 500.4025020 MHz

====== GRADIENT CHANNEL =====
GPNAM1 SINE.100
GPNAM2 SINE.100
GPX1 10.00 %
GPX2 10.00 %
GPY1 10.00 %
GPY2 10.00 %
GPZ1 10.00 %
GPZ2 10.00 %
P16 1000.00 usec

F1 - Acquisition parameters
ND0 1
TD 512
SFO1 500.4025 MHz
FIDRES 10.707922 Hz
SW 10.956 ppm
FnMODE QF

F2 - Processing parameters
SI 1024
SF 500.4000235 MHz
WDW SINE
SSB 0
LB 0.00 Hz
GB 0
PC 1.00

F1 - Processing parameters
SI 1024
MC2 QF
SF 500.4000235 MHz
WDW SINE
SSB 0
LB 0.00 Hz
GB 0

THNemertelline HMQC

HMQC

HPLC Peak #9, Concentrated from 0.6 ml, About 0.7 mg, 0.18 ml WK's CDCl3 (99.96% D)
William Kem and Ferenc Soti, Department of Pharmacology and Therapeutics, 392-0669, Enc: Kem-002
2.5 mm TXI Probe, Temp= 27 C, Non Spun, Bruker Avance 500 Console, Magnex 11.75 T/54 mm Magnet
Advanced Magnetic Resonance Imaging and Spectroscopy, McKnight Brain Institute, University of Florida
Jim Rocca, AMRIS

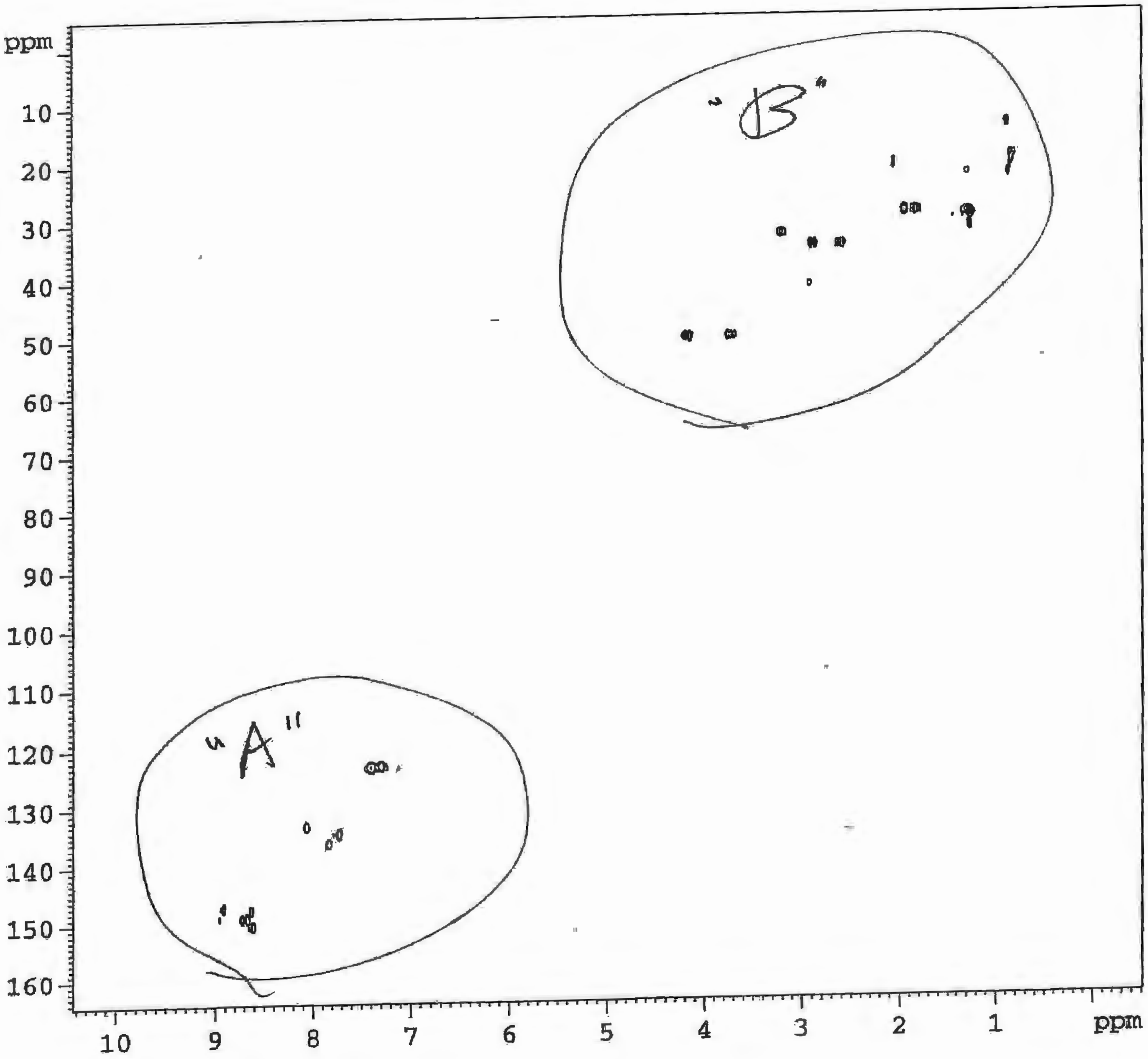

Current Data Parameters
NAME WK_27Jun07(#9)CDCl3
EXPNO 5
PROCNO 1

F2 - Acquisition Parameters
Date_ 20070702
Time 3.08
INSTRUM spect
PROBHD 2.5 mm TXI 1H/
PULPROG hmqcbiph
TD 2048
SOLVENT CDCl3
NS 32
DS 4
SWH 5482.456 Hz
FIDRES 2.676980 Hz
AQ 0.1869188 sec
RG 7168
DW 91.200 usec
DE 10.00 usec
TE 300.0 K
CNST2 145.0000000
d0 0.00000300 sec
D1 1.50000000 sec
d2 0.00344828 sec
D7 0.50000000 sec
IN0 0.00002337 sec
MCREST 0.00000000 sec
MCWRK 1.50000000 sec

======== CHANNEL f1 ========
NUC1 1H
P1 8.00 usec
p2 16.00 usec
PL1 6.00 dB
SFO1 500.4025020 MHz

======== CHANNEL f2 ========
CPDPRG2 garp4
NUC2 13C
P3 14.00 usec
p4 28.00 usec
PCPD2 70.00 usec
PL2 0.00 dB
PL12 14.00 dB
SFO2 125.8356961 MHz

F1 - Acquisition parameters
ND0 2
TD 320
SFO1 125.8357 MHz
FIDRES 66.844917 Hz
SW 169.987 ppm
FnMODE TPPI

F2 - Processing parameters
SI 1024
SF 500.4000221 MHz
WDW QSINE
SSB 2
LB 0.00 Hz
GB 0
PC 1.00

F1 - Processing parameters
SI 512
MC2 TPPI
SF 125.8256883 MHz
WDW QSINE
SSB 2
LB 0.00 Hz
GB 0

Tetrahydronemertelline "A" Region HMQC

HPLC Peak #9, Concentrated from 0.6 ml, About 0.7 mg, 0.18 ml WK's CDCl3 (99.96% D)
William Kem and Ferenc Soti, Department of Pharmacology and Therapeutics, 392-0669, Enc: Kem-002
2.5 mm TXI Probe, Temp= 27 C, Non Spun, Bruker Avance 500 Console, Magnex 11.75 T/54 mm Magnet
Advanced Magnetic Resonance Imaging and Spectroscopy, McKnight Brain Institute, University of Florida
Jim Rocca, AMRIS

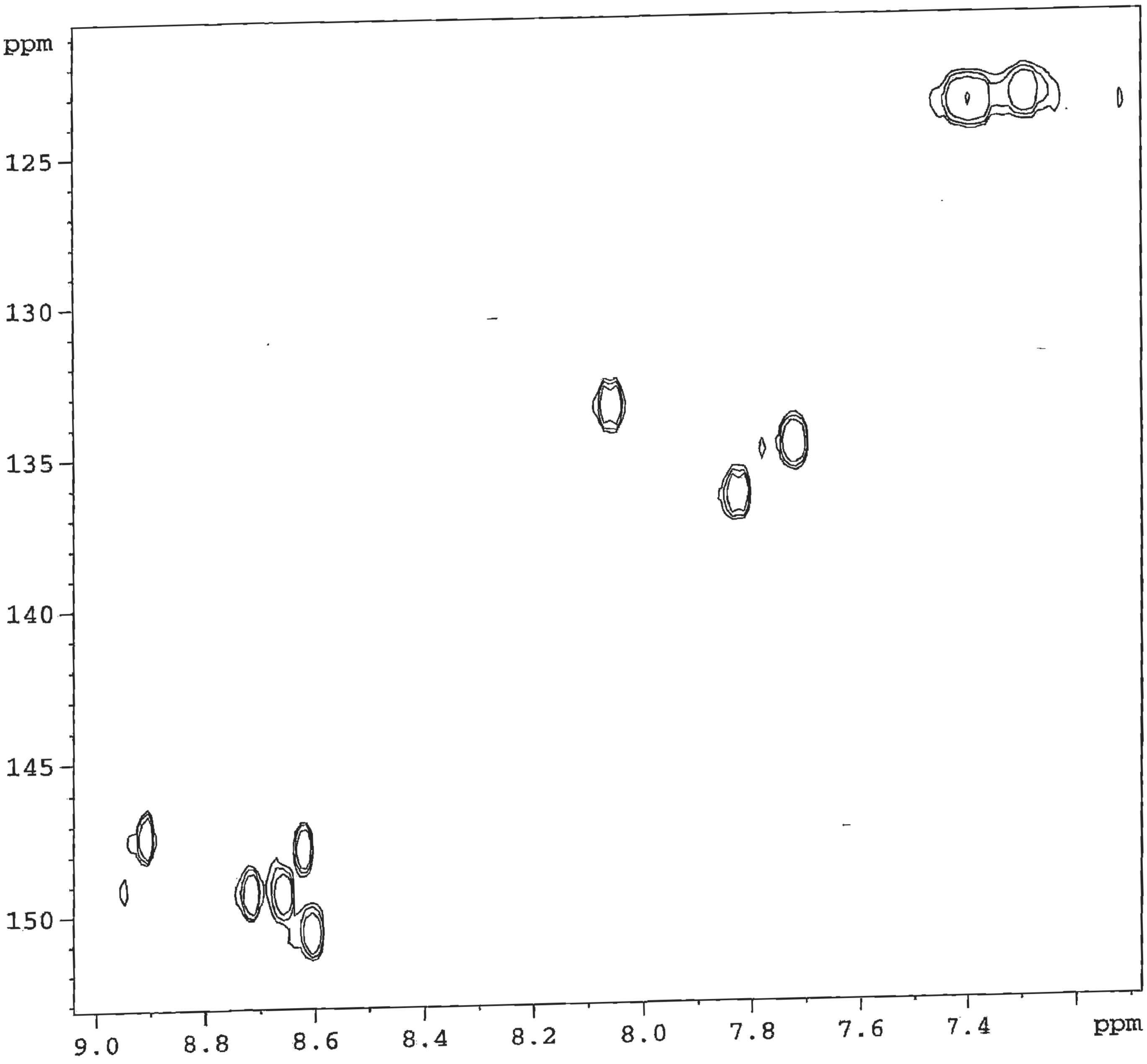

Current Data Parameters

NAME	WK_27Jun07_#9_CDCl3
EXPNO	6
PROCNO	1

F2 - Acquisition Parameters

Date_	20070702	
Time	3.08	
INSTRUM	spect	
PROBHD	2.5 mm TXI 1H/	
PULPROG	hmqcbiph	
TD	2048	
SOLVENT	CDCl3	
NS	32	
DS	4	
SWH	5482.456	Hz
FIDRES	2.676980	Hz
AQ	0.1869188	sec
RG	7168	
DW	91.200	usec
DE	10.00	usec
TE	300.0	K
CNST2	145.0000000	
d0	0.00000300	sec
D1	1.50000000	sec
d2	0.00344828	sec
D7	0.50000000	sec
IN0	0.00002337	sec
MCREST	0.00000000	sec
MCWRK	1.50000000	sec

======== CHANNEL f1 ========

NUC1	1H	
P1	8.00	usec
p2	16.00	usec
PL1	6.00	dB
SFO1	500.4025020	MHz

======== CHANNEL f2 ========

CPDPRG2	garp4	
NUC2	13C	
P3	14.00	usec
p4	28.00	usec
PCPD2	70.00	usec
PL2	0.00	dB
PL12	14.00	dB
SFO2	125.8356961	MHz

F1 - Acquisition parameters

ND0	2	
TD	320	
SFO1	125.8357	MHz
FIDRES	66.844917	Hz
SW	169.987	ppm
FnMODE	TPPI	

F2 - Processing parameters

SI	1024	
SF	500.4000221	MHz
WDW	QSINE	
SSB	2	
LB	0.00	Hz
GB	0	
PC	1.00	

F1 - Processing parameters

SI	512	
MC2	TPPI	
SF	125.8256883	MHz
WDW	QSINE	
SSB	2	
LB	0.00	Hz
GB	0	

Tetrahydronemertelline "B" Region HMQC

HPLC Peak #9, Concentrated from 0.6 ml, About 0.7 mg, 0.18 ml WK's CDCl3 (99.96% D)
William Kem and Ferenc Soti, Department of Pharmacology and Therapeutics, 392-0669, Enc: Kem-002
2.5 mm TXI Probe, Temp= 27 C, Non Spun, Bruker Avance 500 Console, Magnex 11.75 T/54 mm Magnet
Advanced Magnetic Resonance Imaging and Spectroscopy, McKnight Brain Institute, University of Florida
Jim Rocca, AMRIS

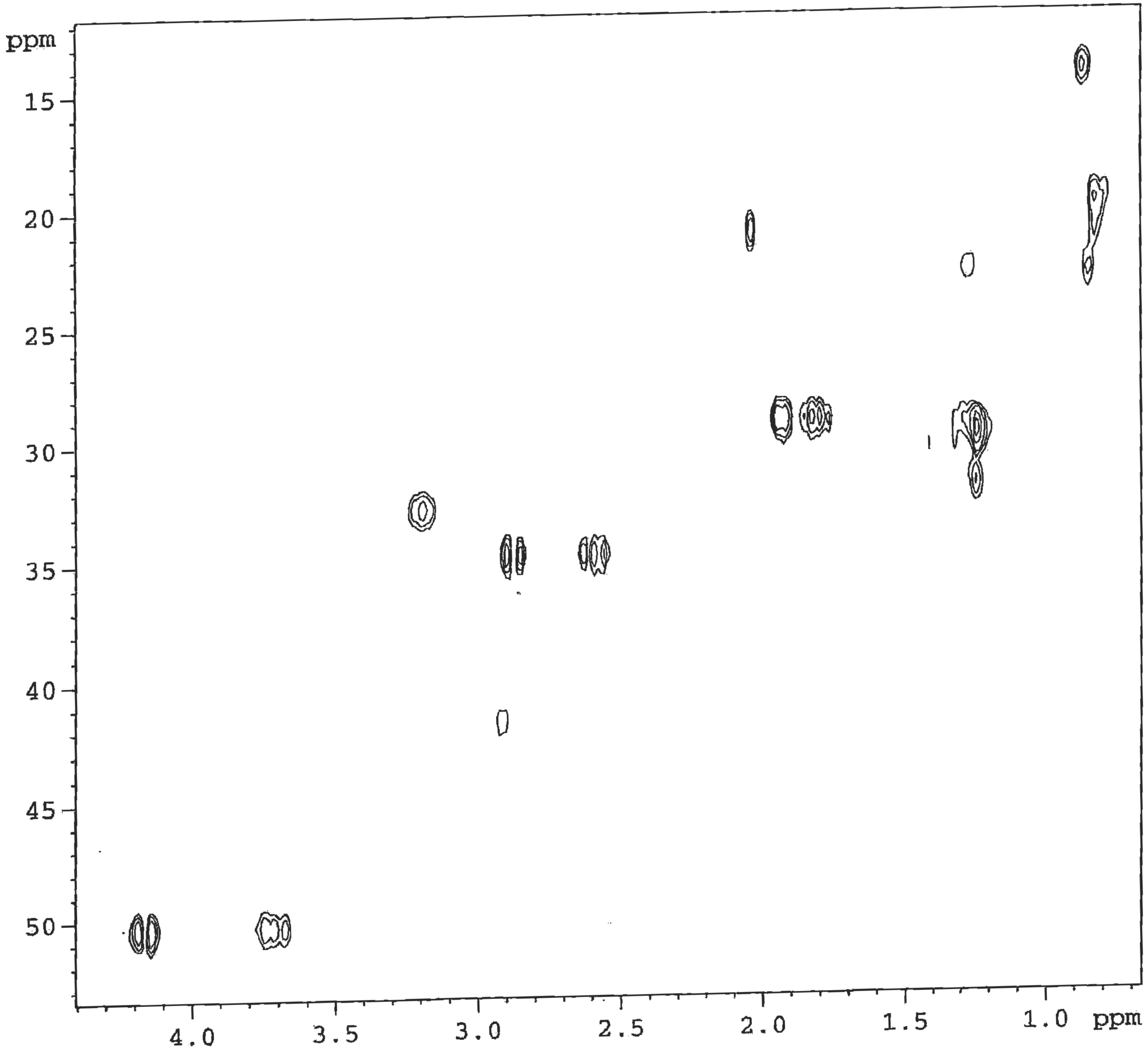

Current Data Parameters
NAME WK_27Jun07_#9_CDCl3
EXPNO 6
PROCNO 1

F2 - Acquisition Parameters
Date_ 20070702
Time 3.08
INSTRUM spect
PROBHD 2.5 mm TXI 1H/
PULPROG hmqcbiph
TD 2048
SOLVENT CDCl3
NS 32
DS 4
SWH 5482.456 Hz
FIDRES 2.676980 Hz
AQ 0.1869188 sec
RG 7168
DW 91.200 usec
DE 10.00 usec
TE 300.0 K
CNST2 145.0000000
d0 0.00000300 sec
D1 1.50000000 sec
d2 0.00344828 sec
D7 0.50000000 sec
IN0 0.00002337 sec
MCREST 0.00000000 sec
MCWRK 1.50000000 sec

======== CHANNEL f1 ========
NUC1 1H
P1 8.00 usec
p2 16.00 usec
PL1 6.00 dB
SFO1 500.4025020 MHz

======== CHANNEL f2 ========
CPDPRG2 garp4
NUC2 13C
P3 14.00 usec
p4 28.00 usec
PCPD2 70.00 usec
PL2 0.00 dB
PL12 14.00 dB
SFO2 125.8356961 MHz

F1 - Acquisition parameters
ND0 2
TD 320
SFO1 125.8357 MHz
FIDRES 66.844917 Hz
SW 169.987 ppm
FnMODE TPPI

F2 - Processing parameters
SI 1024
SF 500.4000221 MHz
WDW QSINE
SSB 2
LB 0.00 Hz
GB 0
PC 1.00

F1 - Processing parameters
SI 512
MC2 TPPI
SF 125.8256883 MHz
WDW QSINE
SSB 2
LB 0.00 Hz
GB 0

Tetrahydronemertelline HMBC

HIIBC

HPLC Peak #9, Concentrated from 0.6 ml, About 0.7 mg, 0.18 ml WK's CDCl3 (99.96% D)
William Kem and Ferenc Soti, Department of Pharmacology and Therapeutics, 392-0669, Enc: Kem-002
2.5 mm TXI Probe, Temp= 27 C, Non Spun, Bruker Avance 500 Console, Magnex 11.75 T/54 mm Magnet
Advanced Magnetic Resonance Imaging and Spectroscopy, McKnight Brain Institute, University of Florida
Jim Rocca, AMRIS

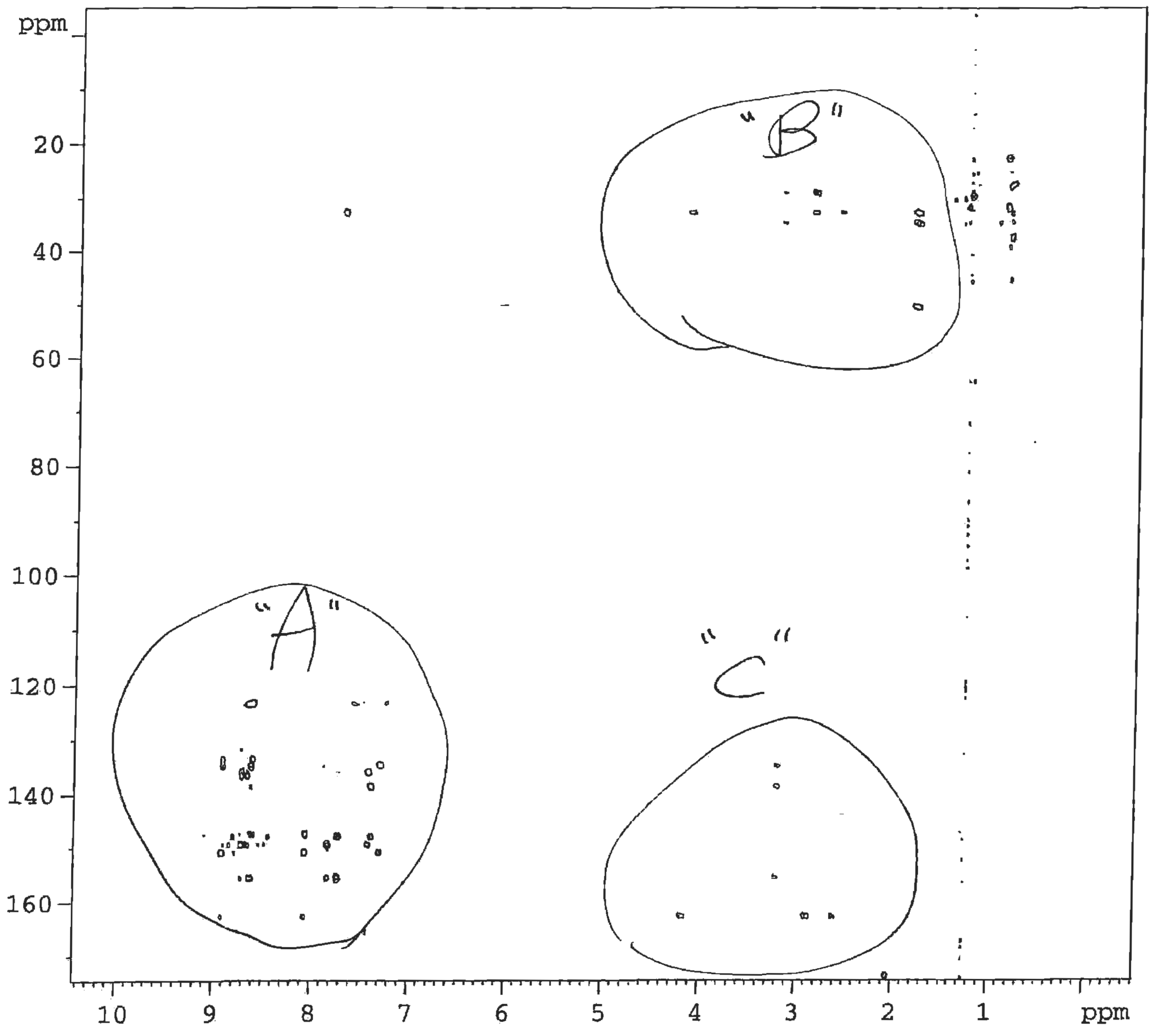

Current Data Parameters
NAME WK_27Jun07_#9_CDCl3
EXPNO 5
PROCNO 1

F2 - Acquisition Parameters
Date_ 20070630
Time 12.34
INSTRUM spect
PROBHD 2.5 mm TXI 1H/
PULPROG hmbclpndqf
TD 2048
SOLVENT CDCl3
NS 160
DS 4
SWH 5482.456 Hz
FIDRES 2.676980 Hz
AQ 0.1869188 sec
RG 7168
DW 91.200 usec
DE 10.00 usec
TE 300.0 K
CNST2 145.0000000
CNST13 8.0000000
d0 0.00000300 sec
D1 2.00000000 sec
d2 0.00344828 sec
d6 0.06250000 sec
IN0 0.00002208 sec
MCREST 0.00000000 sec
MCWRK 2.00000000 sec

======== CHANNEL f1 ========
NUC1 1H
P1 8.00 usec
p2 16.00 usec
PL1 6.00 dB
SFO1 500.4025020 MHz

======== CHANNEL f2 ========
NUC2 13C
P3 14.00 usec
PL2 0.00 dB
SFO2 125.8363252 MHz

F1 - Acquisition parameters
ND0 2
TD 384
SFO1 125.8363 MHz
FIDRES 58.984524 Hz
SW 179.996 ppm
FnMODE QF

F2 - Processing parameters
SI 1024
SF 500.4000221 MHz
WDW SINE
SSB 1
LB 0.00 Hz
GB 0
PC 1.00

F1 - Processing parameters
SI 512
MC2 QF
SF 125.8256883 MHz
WDW SINE
SSB 1
LB 0.00 Hz
GB 0

Tetrahydronemertelline HMBC "A" Region

HPLC Peak #9, Concentrated from 0.6 ml, About 0.7 mg, 0.18 ml WK's CDCl3 (99.96% D)

William Kem and Ferenc Soti, Department of Pharmacology and Therapeutics, 392-0669, Enc: Kem-002

2.5 mm TXI Probe, Temp= 27 C, Non Spun, Bruker Avance 500 Console, Magnex 11.75 T/54 mm Magnet

Advanced Magnetic Resonance Imaging and Spectroscopy, McKnight Brain Institute, University of Florida
Jim Rocca, AMRIS

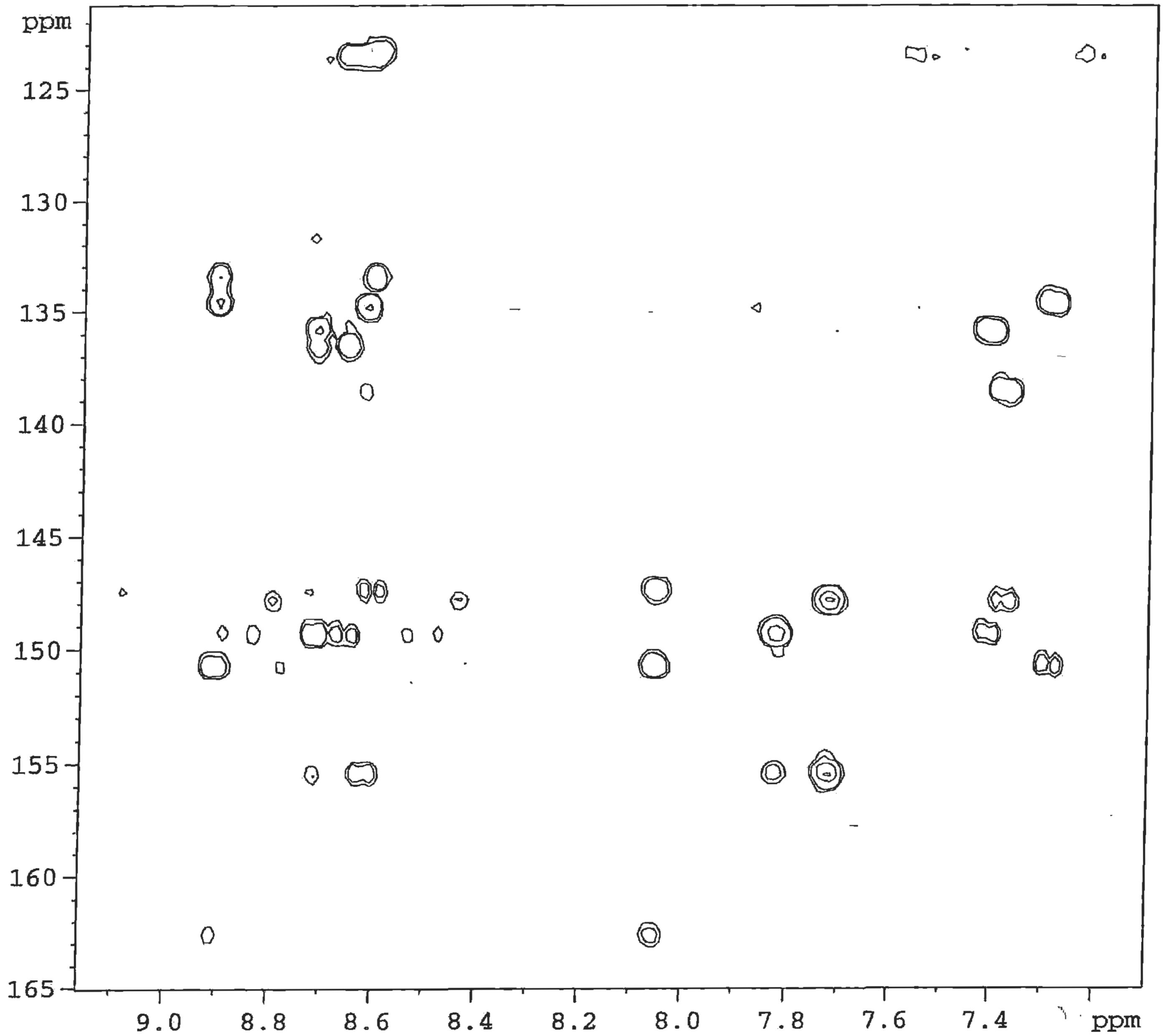

Current Data Parameters

NAME	WK_27Jun07_#9_CDCl3
EXPNO	5
PROCNO	1

F2 - Acquisition Parameters

Date_	20070630
Time	12.34
INSTRUM	spect
PROBHD	2.5 mm TXI 1H/
PULPROG	hmbclpndqf
TD	2048
SOLVENT	CDCl3
NS	160
DS	4
SWH	5482.456 Hz
FIDRES	2.676980 Hz
AQ	0.1869188 sec
RG	7168
DW	91.200 usec
DE	10.00 usec
TE	300.0 K
CNST2	145.0000000
CNST13	8.0000000
d0	0.00000300 sec
D1	2.00000000 sec
d2	0.00344828 sec
d6	0.06250000 sec
IN0	0.00002208 sec
MCREST	0.00000000 sec
MCWRK	2.00000000 sec

======== CHANNEL f1 ========

NUC1	1H
P1	8.00 usec
p2	16.00 usec
PL1	6.00 dB
SFO1	500.4025020 MHz

======== CHANNEL f2 ========

NUC2	13C
P3	14.00 usec
PL2	0.00 dB
SFO2	125.8363252 MHz

F1 - Acquisition parameters

ND0	2
TD	384
SFO1	125.8363 MHz
FIDRES	58.984524 Hz
SW	179.996 ppm
FnMODE	QF

F2 - Processing parameters

SI	1024
SF	500.4000221 MHz
WDW	SINE
SSB	1
LB	0.00 Hz
GB	0
PC	1.00

F1 - Processing parameters

SI	512
MC2	QF
SF	125.8256883 MHz
WDW	SINE
SSB	1
LB	0.00 Hz
GB	0

Tetrahydro nemertelline HMBC "B" Region

HPLC Peak #9, Concentrated from 0.6 ml, About 0.7 mg, 0.18 ml WK's CDCl3 (99.96% D)
William Kem and Ferenc Soti, Department of Pharmacology and Therapeutics, 392-0669, Enc: Kem-002
2.5 mm TXI Probe, Temp= 27 C, Non Spun, Bruker Avance 500 Console, Magnex 11.75 T/54 mm Magnet
Advanced Magnetic Resonance Imaging and Spectroscopy, McKnight Brain Institute, University of Florida
Jim Rocca, AMRIS

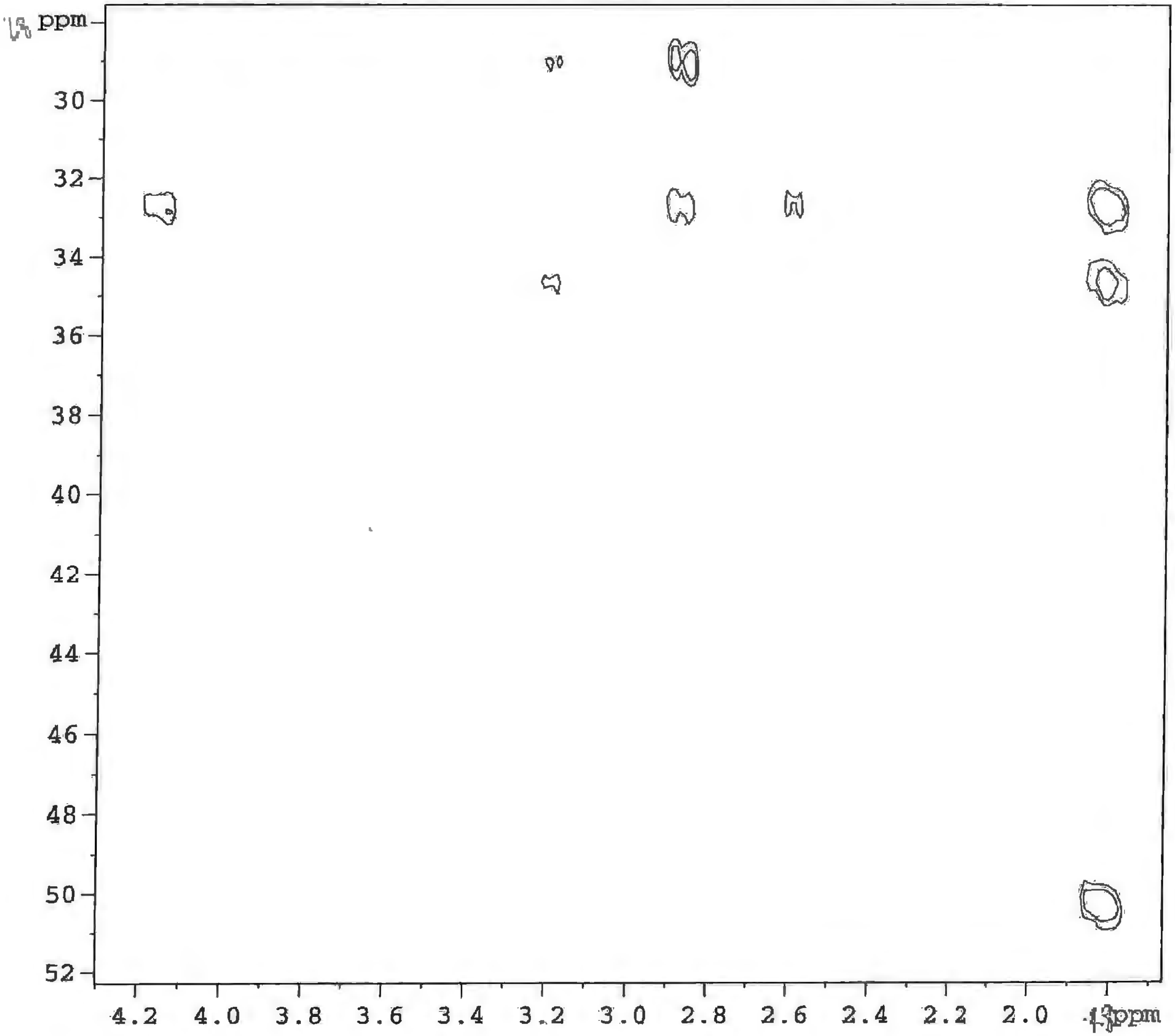

Current Data Parameters

NAME	WK_27Jun07_#9_CDCl3
EXPNO	5
PROCNO	1

F2 - Acquisition Parameters

Date_	20070630	
Time	12.34	
INSTRUM	spect	
PROBHD	2.5 mm TXI 1H/	
PULPROG	hmbclpndqf	
TD	2048	
SOLVENT	CDCl3	
NS	160	
DS	4	
SWH	5482.456	Hz
FIDRES	2.676980	Hz
AQ	0.1869188	sec
RG	7168	
DW	91.200	usec
DE	10.00	usec
TE	300.0	K
CNST2	145.0000000	
CNST13	8.0000000	
d0	0.00000300	sec
D1	2.00000000	sec
d2	0.00344828	sec
d6	0.06250000	sec
IN0	0.00002208	sec
MCREST	0.00000000	sec
MCWRK	2.00000000	sec

======== CHANNEL f1 ========

NUC1	1H	
P1	8.00	usec
p2	16.00	usec
PL1	6.00	dB
SFO1	500.4025020	MHz

======== CHANNEL f2 ========

NUC2	13C	
P3	14.00	usec
PL2	0.00	dB
SFO2	125.8363252	MHz

F1 - Acquisition parameters

ND0	2	
TD	384	
SFO1	125.8363	MHz
FIDRES	58.984524	Hz
SW	179.996	ppm
FnMODE	QF	

F2 - Processing parameters

SI	1024	
SF	500.4000221	MHz
WDW	SINE	
SSB	1	
LB	0.00	Hz
GB	0	
PC	1.00	

F1 - Processing parameters

SI	512	
MC2	QF	
SF	125.8256883	MHz
WDW	SINE	
SSB	1	
LB	0.00	Hz
GB	0	

Tetrahydronemertelline "C" Region HMBC

HPLC Peak #9, Concentrated from 0.6 ml, About 0.7 mg, 0.18 ml WK's CDCl3 (99.96% D)
William Kem and Ferenc Soti, Department of Pharmacology and Therapeutics, 392-0669, Enc: Kem-002
2.5 mm TXI Probe, Temp= 27 C, Non Spun, Bruker Avance 500 Console, Magnex 11.75 T/54 mm Magnet
Advanced Magnetic Resonance Imaging and Spectroscopy, McKnight Brain Institute, University of Florida
Jim Rocca, AMRIS

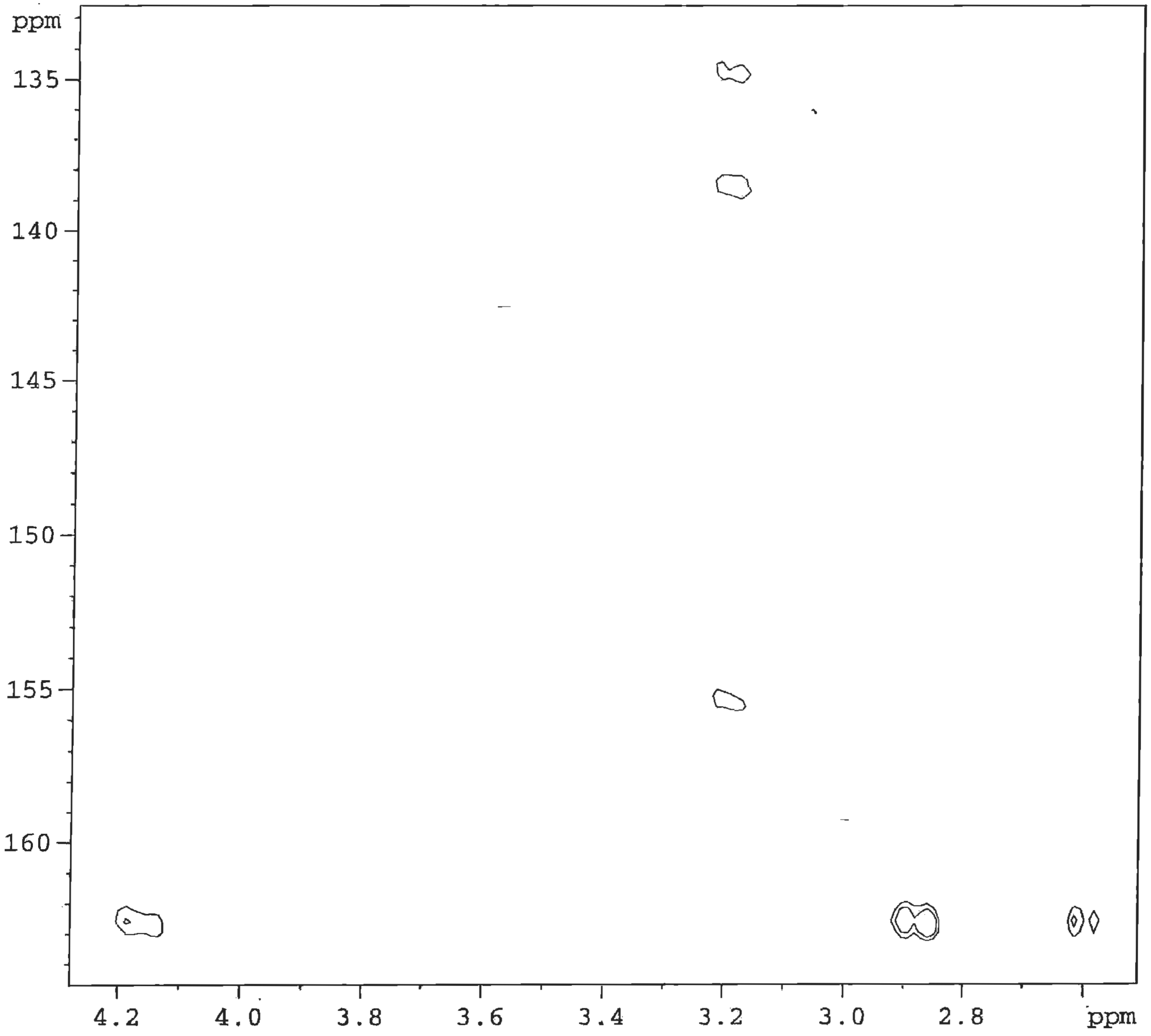

Current Data Parameters
NAME WK_27Jun07_#9_CDCl3
EXPNO 5
PROCNO 1

F2 - Acquisition Parameters
Date_ 20070630
Time 12.34
INSTRUM spect
PROBHD 2.5 mm TXI 1H/
PULPROG hmbclpndqf
TD 2048
SOLVENT CDCl3
NS 160
DS 4
SWH 5482.456 Hz
FIDRES 2.676980 Hz
AQ 0.1869188 sec
RG 7168
DW 91.200 usec
DE 10.00 usec
TE 300.0 K
CNST2 145.0000000
CNST13 8.0000000
d0 0.00000300 sec
D1 2.00000000 sec
d2 0.00344828 sec
d6 0.06250000 sec
IN0 0.00002208 sec
MCREST 0.00000000 sec
MCWRK 2.00000000 sec

======== CHANNEL f1 ========
NUC1 1H
P1 8.00 usec
p2 16.00 usec
PL1 6.00 dB
SFO1 500.4025020 MHz

======== CHANNEL f2 ========
NUC2 13C
P3 14.00 usec
PL2 0.00 dB
SFO2 125.8363252 MHz

F1 - Acquisition parameters
ND0 2
TD 384
SFO1 125.8363 MHz
FIDRES 58.984524 Hz
SW 179.996 ppm
FnMODE QF

F2 - Processing parameters
SI 1024
SF 500.4000221 MHz
WDW SINE
SSB 1
LB 0.00 Hz
GB 0
PC 1.00

F1 - Processing parameters
SI 512
MC2 QF
SF 125.8256883 MHz
WDW SINE
SSB 1
LB 0.00 Hz
GB 0

TH Nem - 6

HPLC Peak #9, Evaporated to Dryness, Estimated About 7 mg (?), Then into 0.6 ml WK's CDCl3 (99.96% D)
William Kem and Ferenc Soti, Department of Pharmacology and Therapeutics, 392-0669, Enc: Kem-002
5 mm BBO Probe, Temp= 27 C, Non Spun, Bruker Avance 500 Console, Magnex 11.75 T/54 mm Magnet
Advanced Magnetic Resonance Imaging and Spectroscopy, McKnight Brain Institute, University of Florida
Jim Rocca, AMRIS

Proton Decoupled 13C NMR spectrum

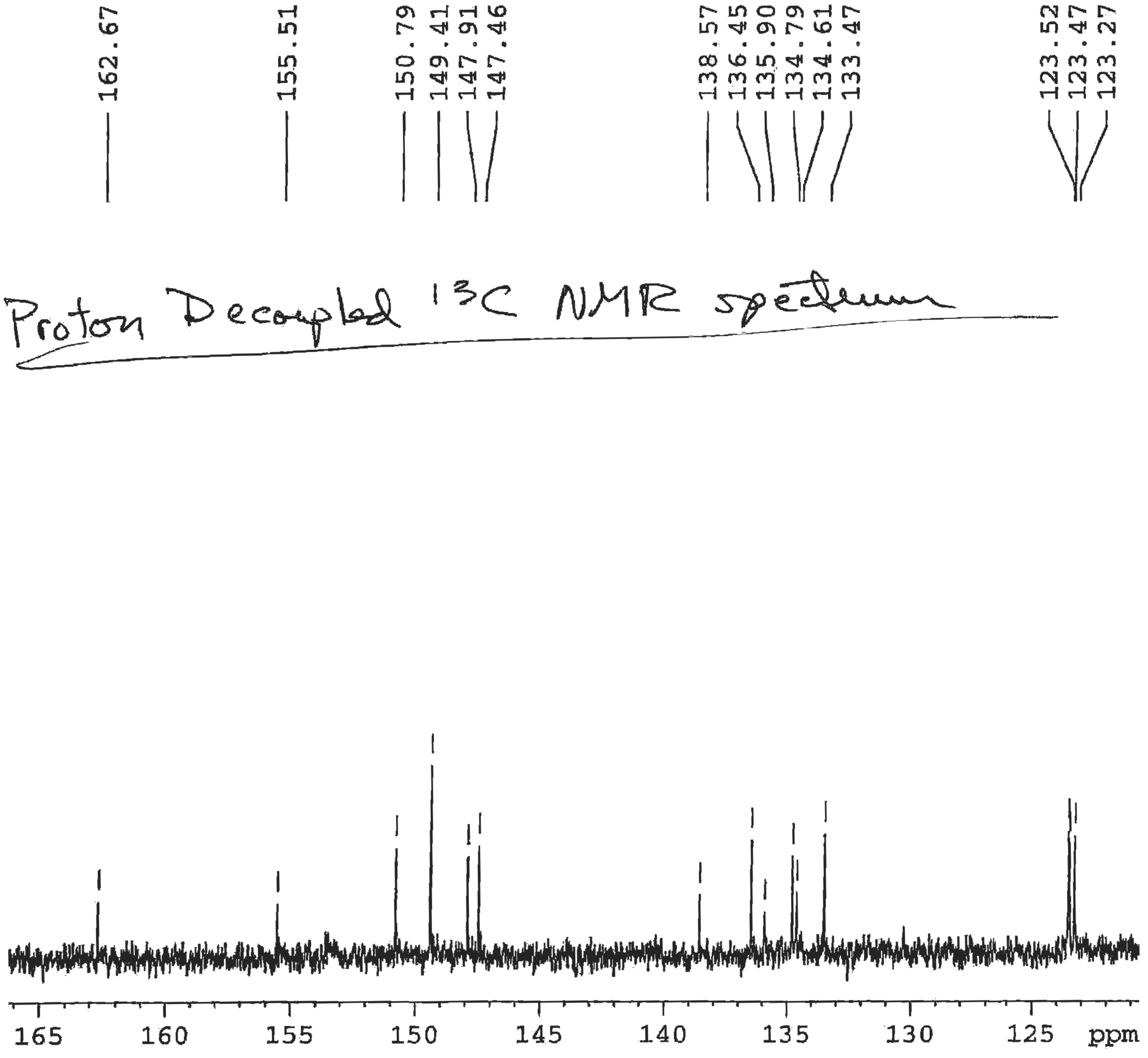

Current Data Parameters
NAME WK_27Jun07_#9_CDC13
EXPNO 5
PROCNO 1

F2 - Acquisition Parameters

Date_	20070628	
Time	6.22	
INSTRUM	spect	
PROBHD	5 mm Multinucl	
PULPROG	zgdc	
TD	65536	
SOLVENT	CDC13	
NS	4096	
DS	1	
SWH	30030.029	Hz
FIDRES	0.458222	Hz
AQ	1.0912410	sec
RG	8192	
DW	16.650	usec
DE	6.00	usec
TE	300.0	K
D1	3.00000000	sec
d11	0.03000000	sec
MCREST	0.00000000	sec
MCWRK	0.01500000	sec

======== CHANNEL f1 ========

NUC1	13C	
P1	3.75	usec
PL1	3.00	dB
SFO1	125.8388380	MHz

======== CHANNEL f2 ========

CPDPRG2	waltz16	
NUC2	1H	
PCPD2	80.00	usec
PL2	120.00	dB
PL12	16.00	dB
SFO2	500.4023010	MHz

F2 - Processing parameters

SI	65536	
SF	125.8256820	MHz
WDW	EM	
SSB	0	
LB	1.50	Hz
GB	0	
PC	1.40	

TH Nem-7

HPLC Peak #9, Evaporated to Dryness, Estimated About 7 mg (?), Then into 0.6 ml WK's CDCl3 (99.96% D)
William Kem and Ferenc Soti, Department of Pharmacology and Therapeutics, 392-0669, Enc: Kem-002
5 mm BBO Probe, Temp= 27 C, Non Spun, Bruker Avance 500 Console, Magnex 11.75 T/54 mm Magnet
Advanced Magnetic Resonance Imaging and Spectroscopy, McKnight Brain Institute, University of Florida
Jim Rocca, AMRIS

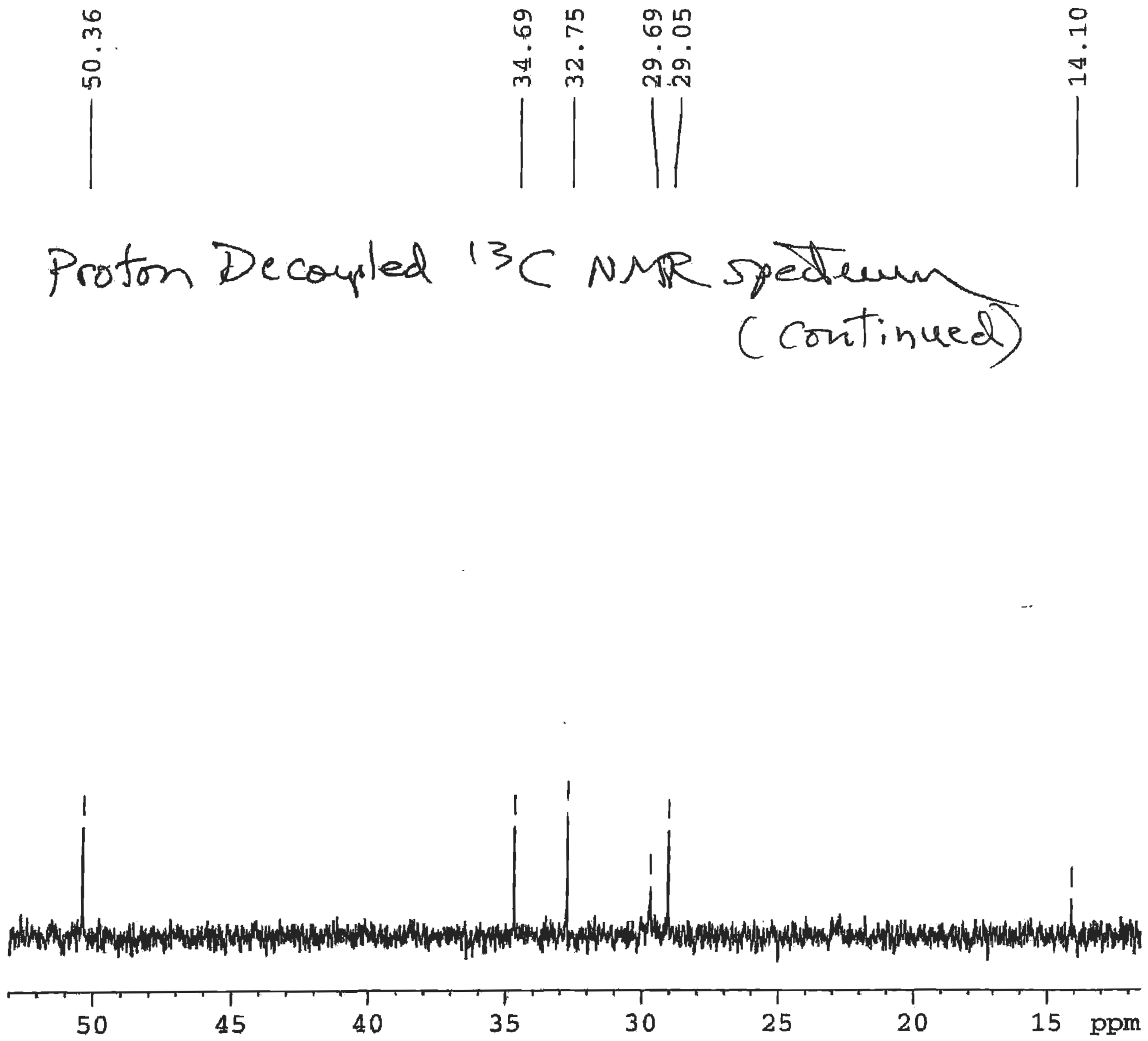

Current Data Parameters

NAME	WK_27Jun07_#9_CDCl3
EXPNO	5
PROCNO	1

F2 - Acquisition Parameters

Date_	20070628
Time	6.22
INSTRUM	spect
PROBHD	5 mm Multinucl
PULPROG	zgdc
TD	65536
SOLVENT	CDCl3
NS	4096
DS	1
SWH	30030.029 Hz
FIDRES	0.458222 Hz
AQ	1.0912410 sec
RG	8192
DW	16.650 usec
DE	6.00 usec
TE	300.0 K
D1	3.00000000 sec
d11	0.03000000 sec
MCREST	0.00000000 sec
MCWRK	0.01500000 sec

======== CHANNEL f1 ========

NUC1	13C
P1	3.75 usec
PL1	3.00 dB
SFO1	125.8388380 MHz

======== CHANNEL f2 ========

CPDPRG2	waltz16
NUC2	1H
PCPD2	80.00 usec
PL2	120.00 dB
PL12	16.00 dB
SFO2	500.4023010 MHz

F2 - Processing parameters

SI	65536
SF	125.8256820 MHz
WDW	EM
SSB	0
LB	1.50 Hz
GB	0
PC	1.40

Dehydro Nemertelline - A

— 1

"Aa m/z=308"/CDCl3, 7.1 ul, 27mm in 0.58 mm ID Tube, William Kem, Pharmacology and Therapeutics
1 mm TXI CryoProbe, Bruker Avance II 600 Console, Magnex 14.1 T/54 mm AS Magnet
Advanced Magnetic Resonance Imaging and Spectroscopy, McKnight Brain Institute, University of Florida
Jim Rocca, AMRIS

PMR Spectrum

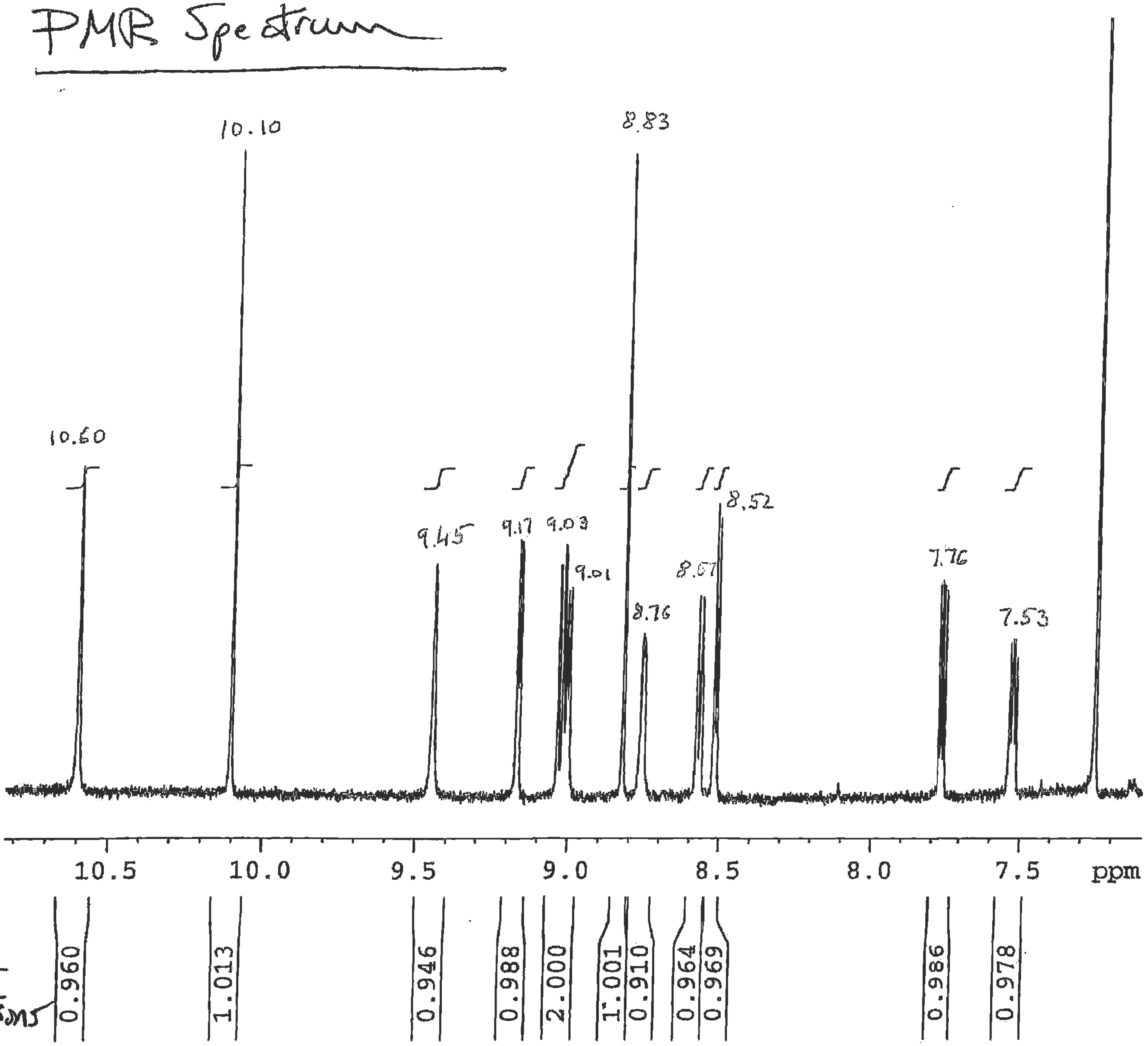

Peak Integrations

NAME	WK_28May08_Aa-mz308
EXPNO	2
PROCNO	1
Date_	20080529
Time	15.53
INSTRUM	spect
PROBHD	1 mm CPTXI 1H/
PULPROG	zg
TD	49152
SOLVENT	CDCl3
NS	64
DS	1
SWH	7812.500 Hz
FIDRES	0.158946 Hz
AQ	3.1457779 sec
RG	128
DW	64.000 usec
DE	6.00 usec
TE	305.2 K
D1	3.00000000 sec
TD0	1

======== CHANNEL f1 ========

NUC1	1H
P1	6.00 usec
PL1	15.00 dB
PL1W	0.46388769 W
SFO1	600.2336014 MHz
SI	65536
SF	600.2300200 MHz
WDW	EM
SSB	0
LB	0.33 Hz
GB	0
PC	1.00

NOESY

Dehydro Nemertelline-A

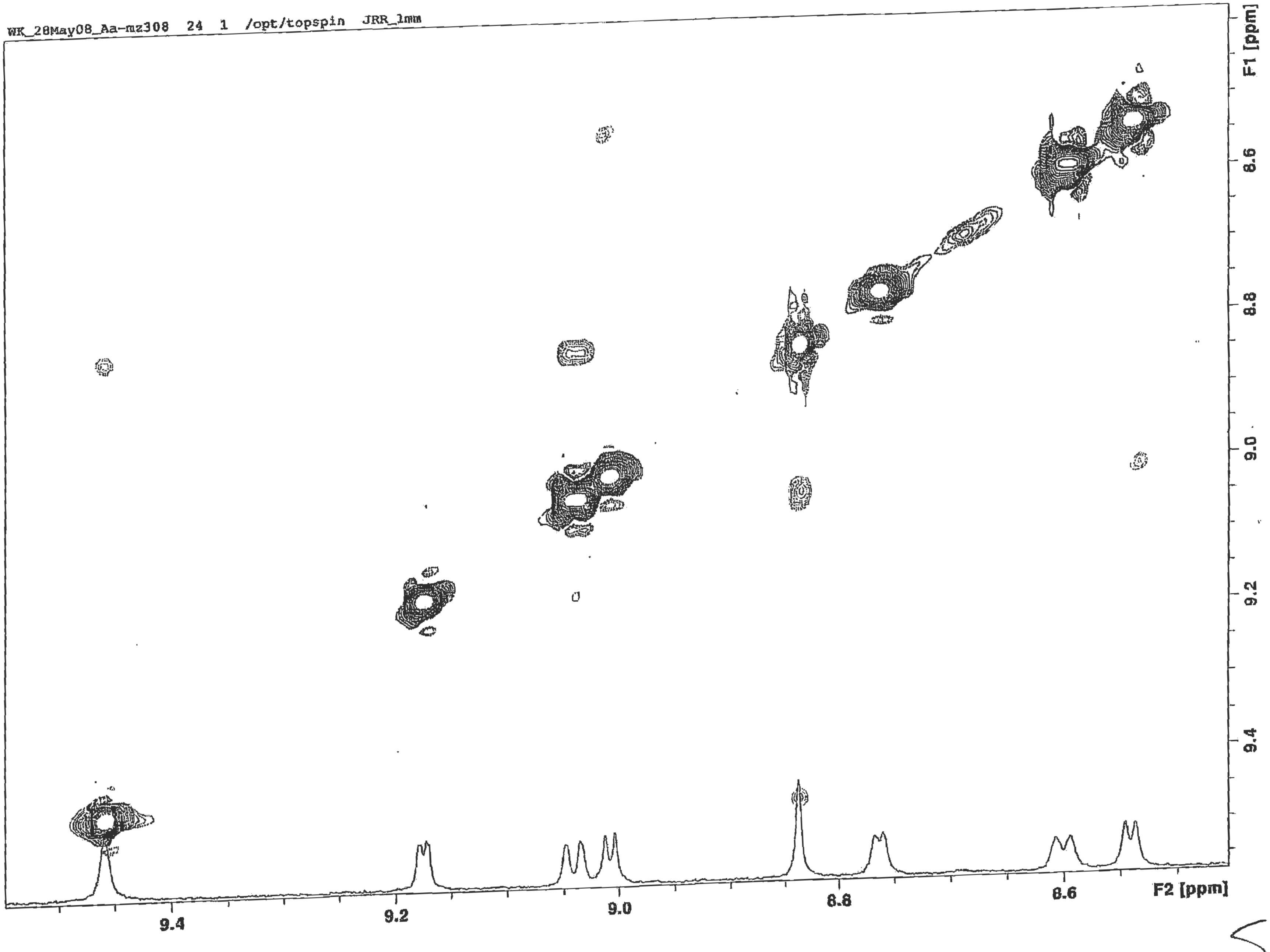

DHN-A-3 HMBC

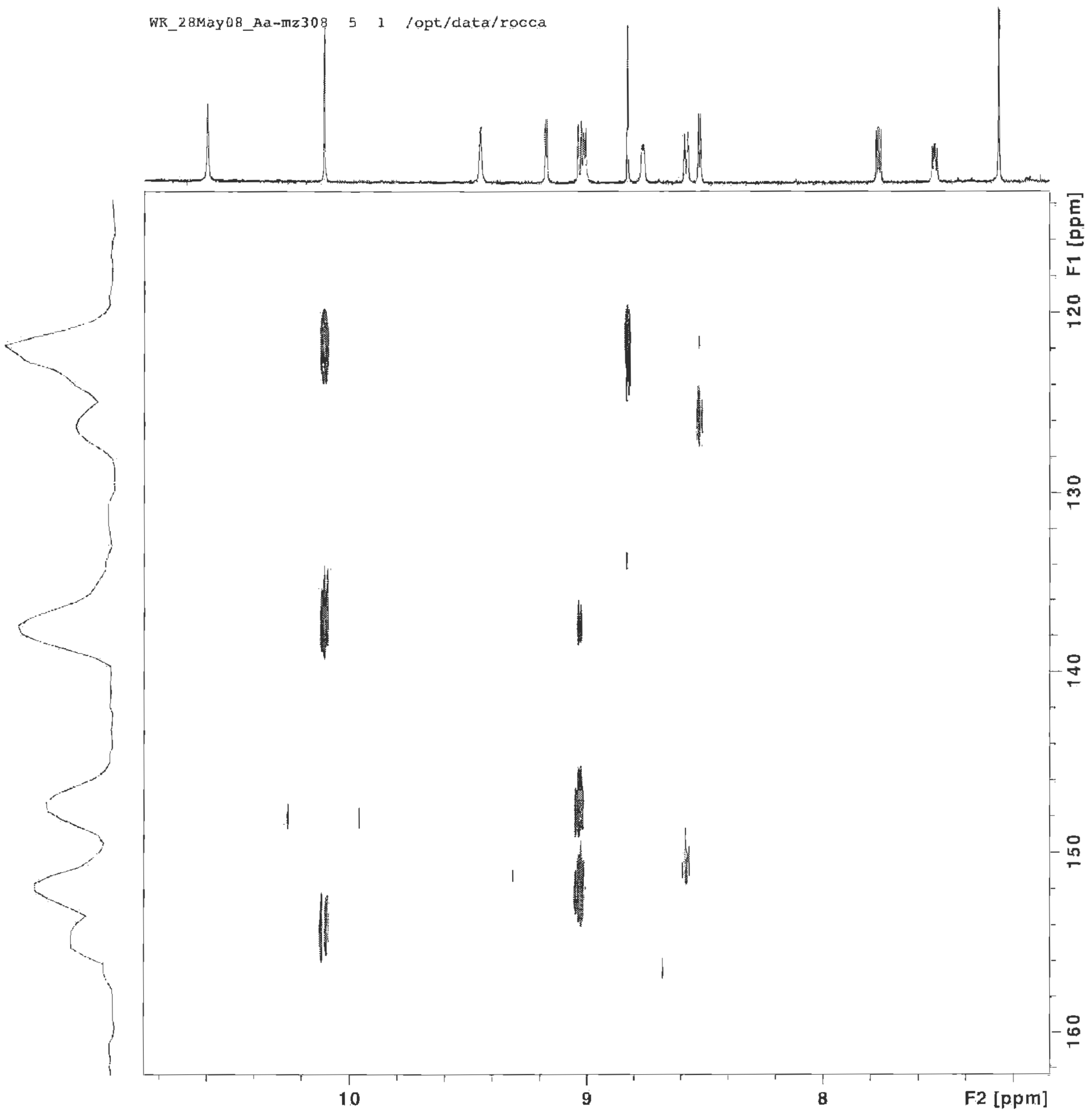

WR_28May08_Aa-mz308 5 1 /opt/data/rocca
F1 [ppm]
120
130
140
150
160
10
9
8
F2 [ppm]

DHN-A-4 HSQC

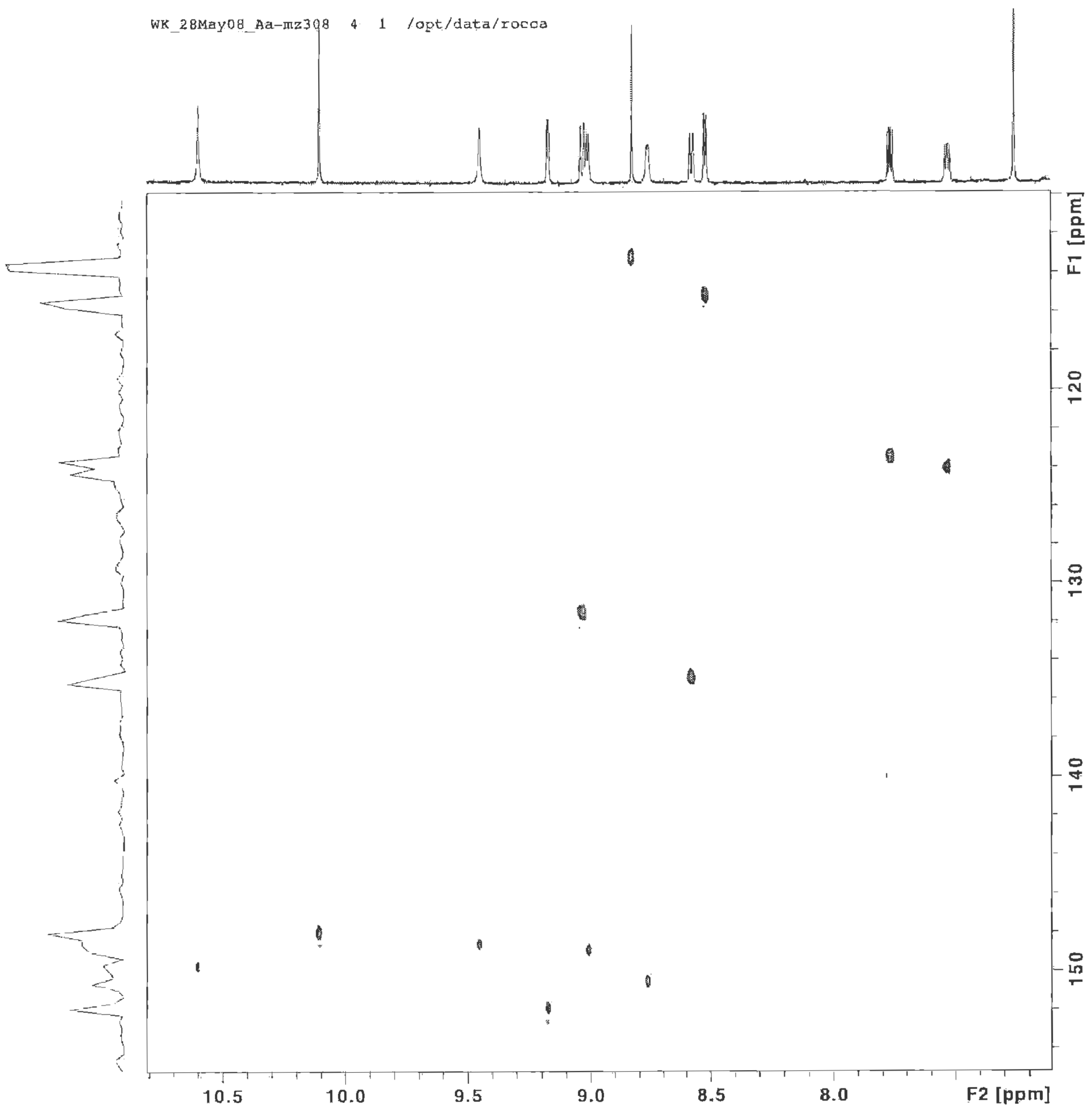
WK_28May08_Aa-mz308 4 1 /opt/data/rocca
F1 [ppm]
120
130
140
150
10.5
10.0
9.5
9.0
8.5
8.0
F2 [ppm]

DH Nem B-1

"Old Aa Pk 3"/CDCl3, 13.4 ul, 32mm in 0.73 mm ID Tube, William Kem, Pharmacology and Therapeutics
1 mm TXI CryoProbe, Bruker Avance II 600 Console, Magnex 14.1 T/54 mm AS Magnet
Advanced Magnetic Resonance Imaging and Spectroscopy, McKnight Brain Institute, University of Florida
Jim Rocca, AMRIS

Dehydro-Nemertelline B PMR Spectrum

NAME	WK_02Jun08_OldAaPk3
EXPNO	1
PROCNO	1
Date_	20080602
Time	13.49
INSTRUM	spect
PROBHD	1 mm CPTXI 1H/
PULPROG	zg
TD	49152
SOLVENT	CDCl3
NS	64
DS	1
SWH	7812.500 Hz
FIDRES	0.158946 Hz
AQ	3.1457779 sec
RG	128
DW	64.000 usec
DE	6.00 usec
TE	305.2 K
D1	3.00000000 sec
TD0	1

======== CHANNEL f1 ========

NUC1	1H
P1	6.00 usec
PL1	15.00 dB
PL1W	0.46388769 W
SFO1	600.2336014 MHz
SI	65536
SF	600.2300191 MHz
WDW	EM
SSB	0
LB	0.33 Hz
GB	0
PC	0.30

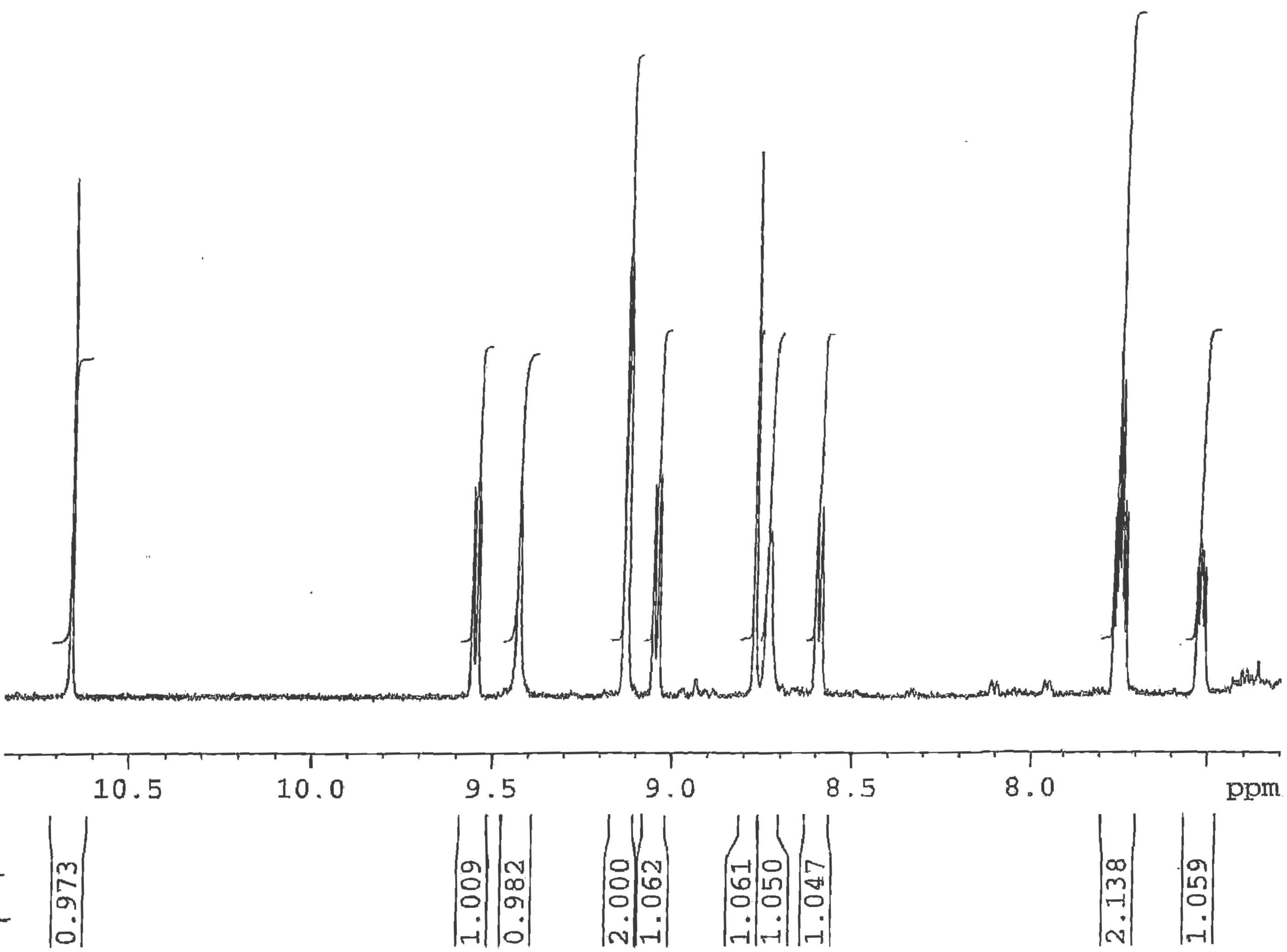

Peak Integrations

S26

COSY DH-NemB-2

"Old Aa Pk 3"/CDCl3, 13.4 ul, 32mm in 0.73 mm ID Tube, William Kem, Pharmacology and Therapeutics
1 mm TXI CryoProbe, Bruker Avance II 600 Console, Magnex 14.1 T/54 mm AS Magnet
Advanced Magnetic Resonance Imaging and Spectroscopy, McKnight Brain Institute, University of Florida
Jim Rocca, AMRIS

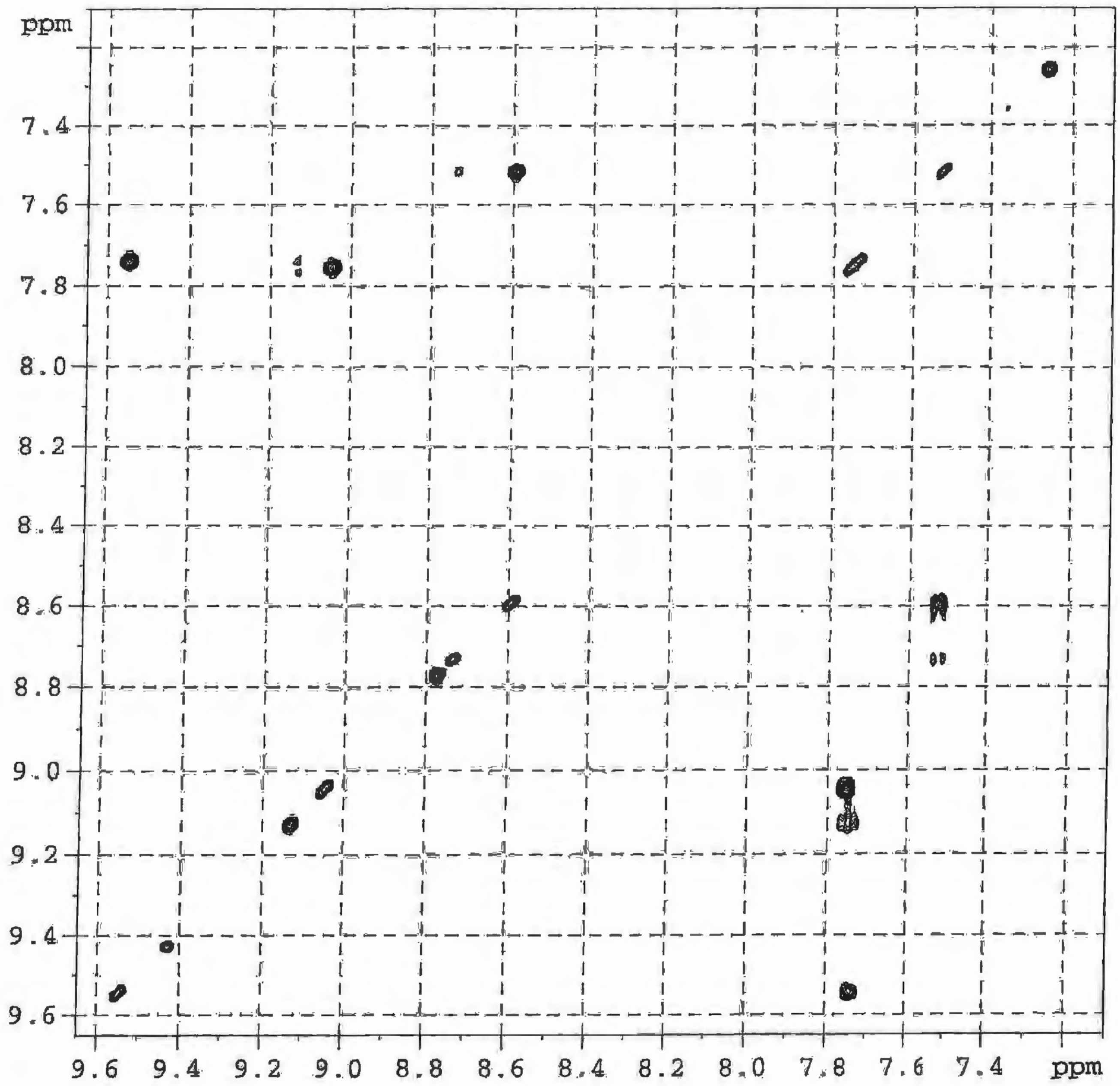

NAME	WK_02Jun08_OldAaPk3
EXPNO	3
PROCNO	1
Date_	20080602
Time	14.11
INSTRUM	spect
PROBHD	1 mm CPTXI 1H/
PULPROG	cosygpqf
TD	2048
SOLVENT	CDCl3
NS	4
DS	4
SWH	7211.539 Hz
FIDRES	3.521259 Hz
AQ	0.1420447 sec
RG	128
DW	69.333 usec
DE	6.00 usec
TE	305.2 K
D0	0.00000300 sec
D1	2.00000000 sec
D13	0.00000400 sec
D16	0.00030000 sec
IN0	0.00013865 sec
======== CHANNEL f1 ========	
NUC1	1H
P0	6.00 usec
P1	12.00 usec
PL1	15.00 dB
PL1W	0.46368769 W
SFO1	600.2333013 MHz
====== GRADIENT CHANNEL =====	
GPNAM1	SINE.100
GPZ1	10.00 %
P16	1000.00 usec
ND0	1
TD	512
SFO1	600.2333 MHz
FIDRES	14.085037 Hz
SW	12.015 ppm
FnMODE	QF
SI	2048
SF	600.2300191 MHz
WDW	SINE
SSB	0
LB	0.00 Hz
GB	0
PC	1.00
SI	2048
MC2	QF
SF	600.2300191 MHz
WDW	SINE
SSB	0
LB	0.00 Hz
GB	0

S27

DH Neum B - 3

NOESY

"Old Aa Pk 3"/CDCl3, 13.4 ul, 32mm in 0.73 mm ID [illegible] William Kem, Pharmacology and Therapeutics
1 mm TXI CryoProbe, Bruker Avance II 600 [illegible]ole, Magnex 14.1 T/54 mm AS Magnet
Advanced Magnetic Resonance Imaging and Spectroscopy, McKnight Brain Institute, University of Florida
Jim Rocca, AMRIS

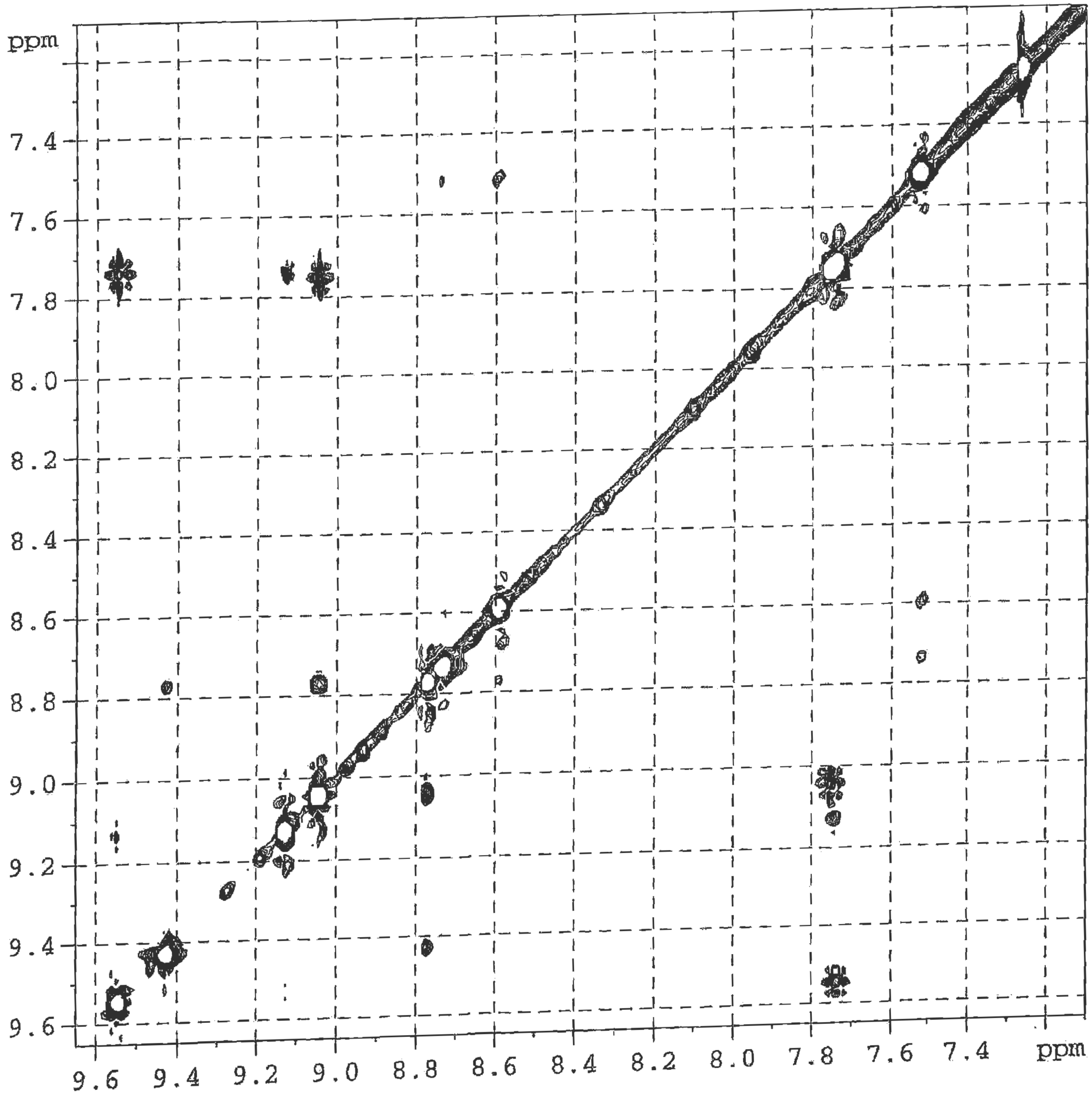

NAME	WK_02Jun08_OldAaPk3
EXPNO	6
PROCNO	1
Date_	20080602
Time	16.57
INSTRUM	spect
PROBHD	1 mm CPTXI 1H/
PULPROG	noesygpph
TD	2048
SOLVENT	CDCl3
NS	48
DS	4
SWH	7211.539 Hz
FIDRES	3.521259 Hz
AQ	0.1420447 sec
RG	64
DW	69.333 usec
DE	6.00 usec
TE	305.2 K
D0	0.00005405 sec
D1	2.00000000 sec
D8	0.40000001 sec
D16	0.00030000 sec
IN0	0.00013865 sec

======== CHANNEL f1 ========

NUC1	1H
P1	12.00 usec
P2	24.00 usec
PL1	15.00 dB
PL1W	0.46388769 W
SFO1	600.2333013 MHz

====== GRADIENT CHANNEL =====

GPNAM1	SINE.100
GPNAM2	SINE.100
GPZ1	40.00 %
GPZ2	-40.00 %
P16	1000.00 usec
ND0	1
TD	512
SFO1	600.2333 MHz
FIDRES	14.085039 Hz
SW	12.015 ppm
FnMODE	States-TPPI
SI	1024
SF	600.2300176 MHz
WDW	QSINE
SSB	2
LB	0.00 Hz
GB	0
PC	1.00
SI	1024
MC2	States-TPPI
SF	600.2300170 MHz
WDW	QSINE
SSB	2
LB	0.00 Hz
GB	0

S28

DHN-B-4 HMBC

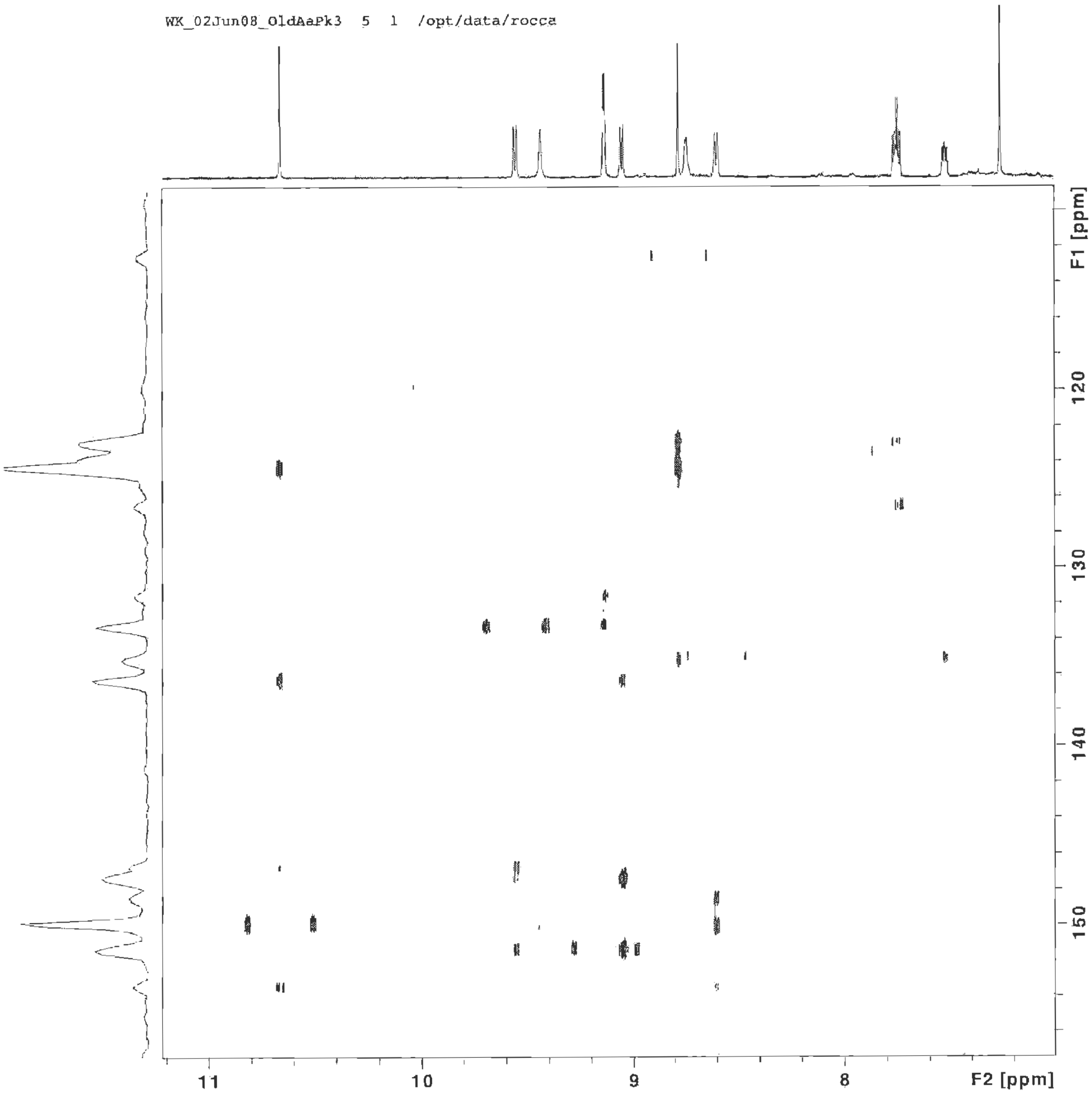

WK_02Jun08_OldAaPk3 5 1 /opt/data/rocca
F1 [ppm]
120
130
140
150
11
10
9
8
F2 [ppm]

DHN-B-5

HSQC

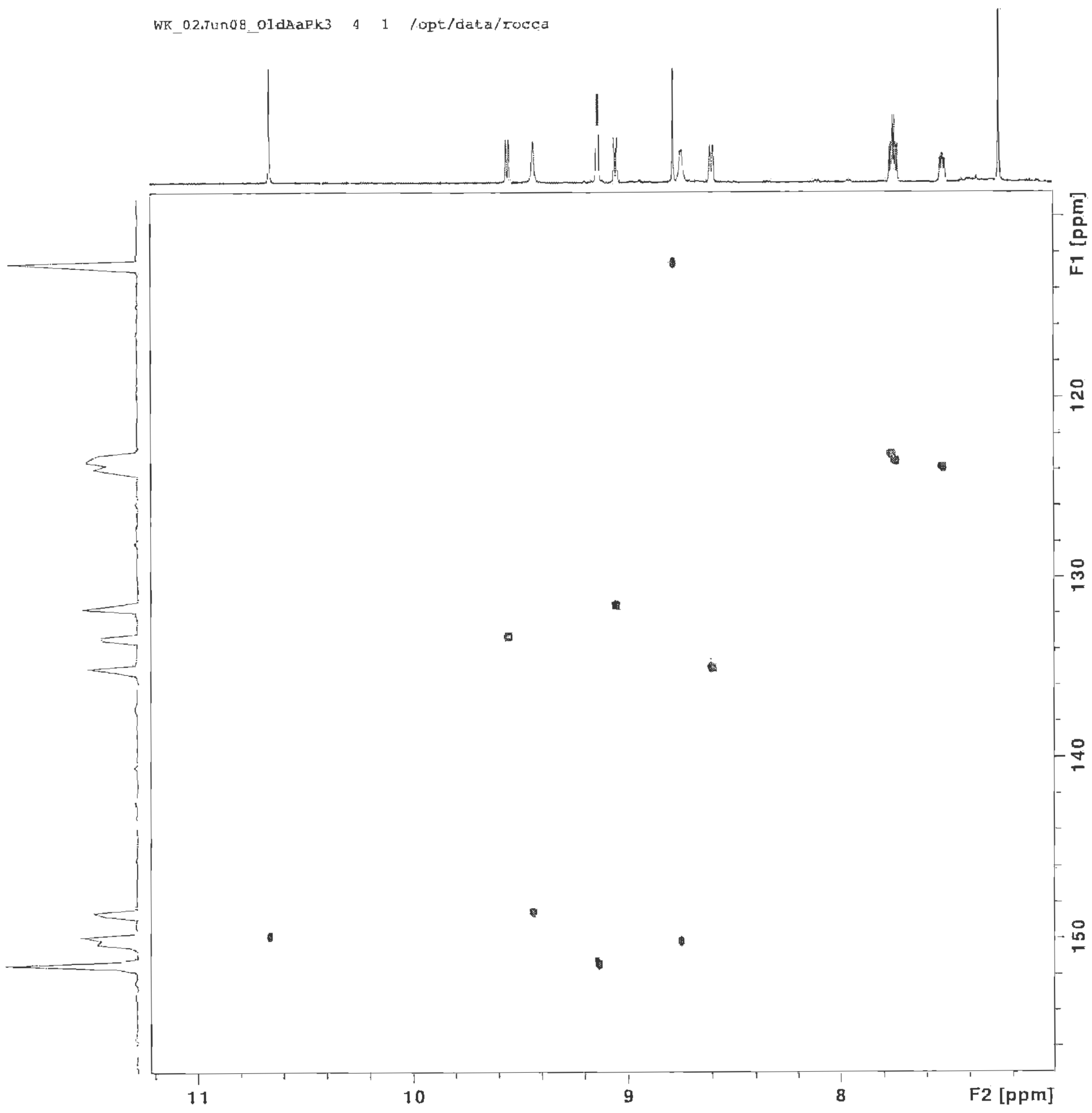

S30

Proton and carbon correlations for the two dehydronemertellines based on HSQC and HMBC NMR analyses. B ring atoms are indicated by ’, C ring atoms by ” and D ring atoms by ‴.

Dehydronemertelline A

Proton Chemical Shifts (ppm)	HSQC Carbon Chemical Shifts (ppm)	HMBC Quaternary Carbons (ppm)
10.60 (2)	149.7	–
10.10 (6”)	148.0	121.7, 137.2*, 154.0
9.45 (2‴)	148.6	–
9.17 (6’)	151.9	–
9.03 (4’)	131.5	137.3*, 147.0
9.01 (6)	148.9	–
8.83 (3‴)	113.2	121.7
8.76 (6‴)	150.6	–
8.57(4‴)	134.9	150.5
8.52 (5)	115.1	125.8
7.76 (5’)	123.4	–
7.53 (5‴)	124.0	–

*Indicates possibility of same quaternary carbon.

Dehydronemertelline B

Proton Chemical Shifts (ppm)	HSQC Carbon Chemical Shifts (ppm)	HMBC Quaternary Carbons (ppm)
10.66 (6”)	149.8	124.3, 136.3, 153.4
9.55 (4)	133.2	146.8
9.43 (2‴)	148.5	–
9.14 (6)	151.2	133.2
9.13 (6’)	152.3	–
9.05 (4’)	131.6	136.3, 147.3
8.77 (3”)	112.5	122.9, 124.2
8.74 (6‴)	150.1	–
8.59 (4‴)	134.9	–
7.76 (5’)	123.1	–
7.75 (3)	123.4	126.5
7.52 (5‴)	123.8	–

DH Nemertelline H and C Correlations

C, 5 mm TXI, Bruker Avance-III-800 Console, Bruker 18.8 T/54 mm Ascend Magnet
Magnetic Resonance Imaging and Spectroscopy, McKnight Brain Institute, University of Florida
AMRIS

Nemertelline Proton NMR Spectrum

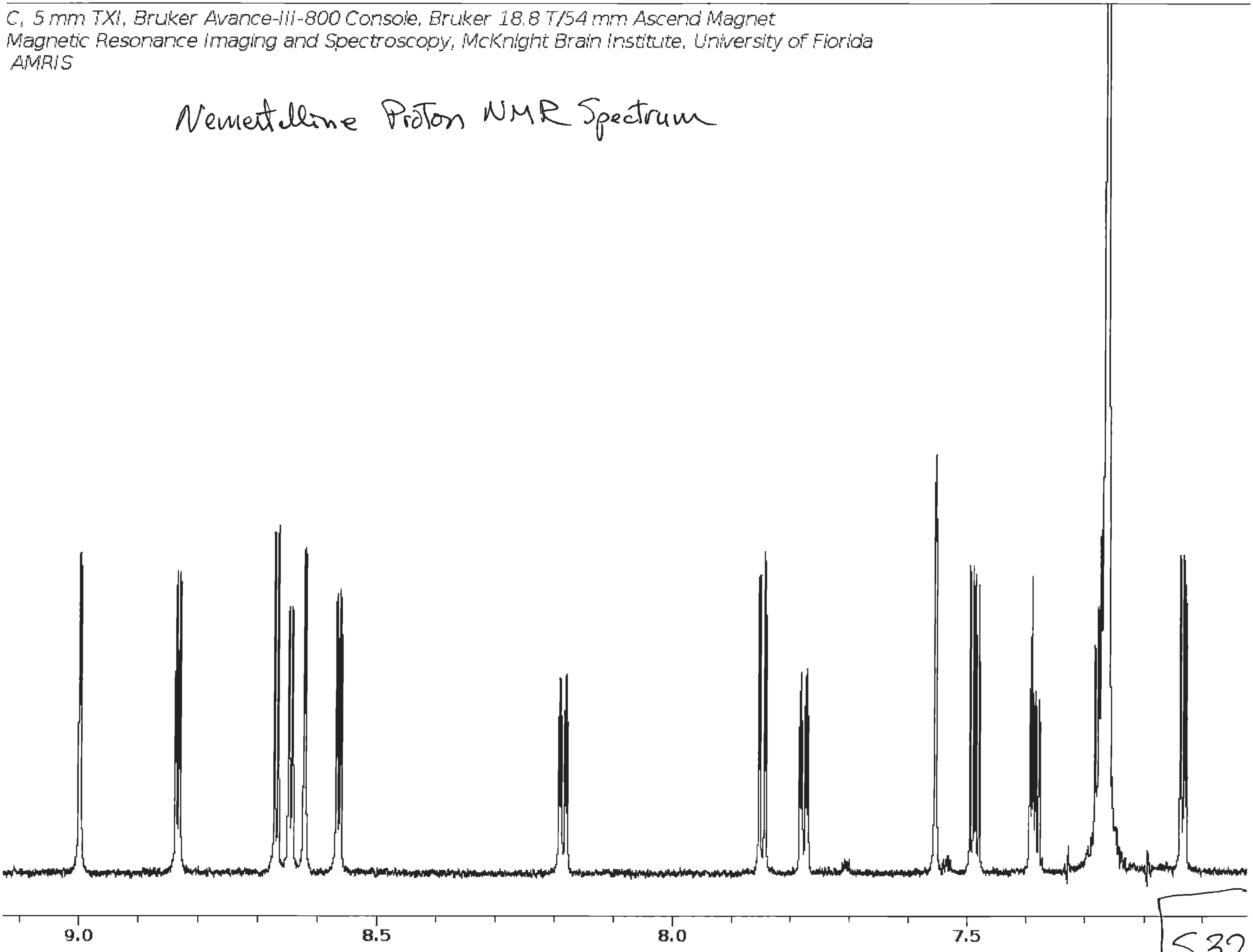

Nemertelline COSY

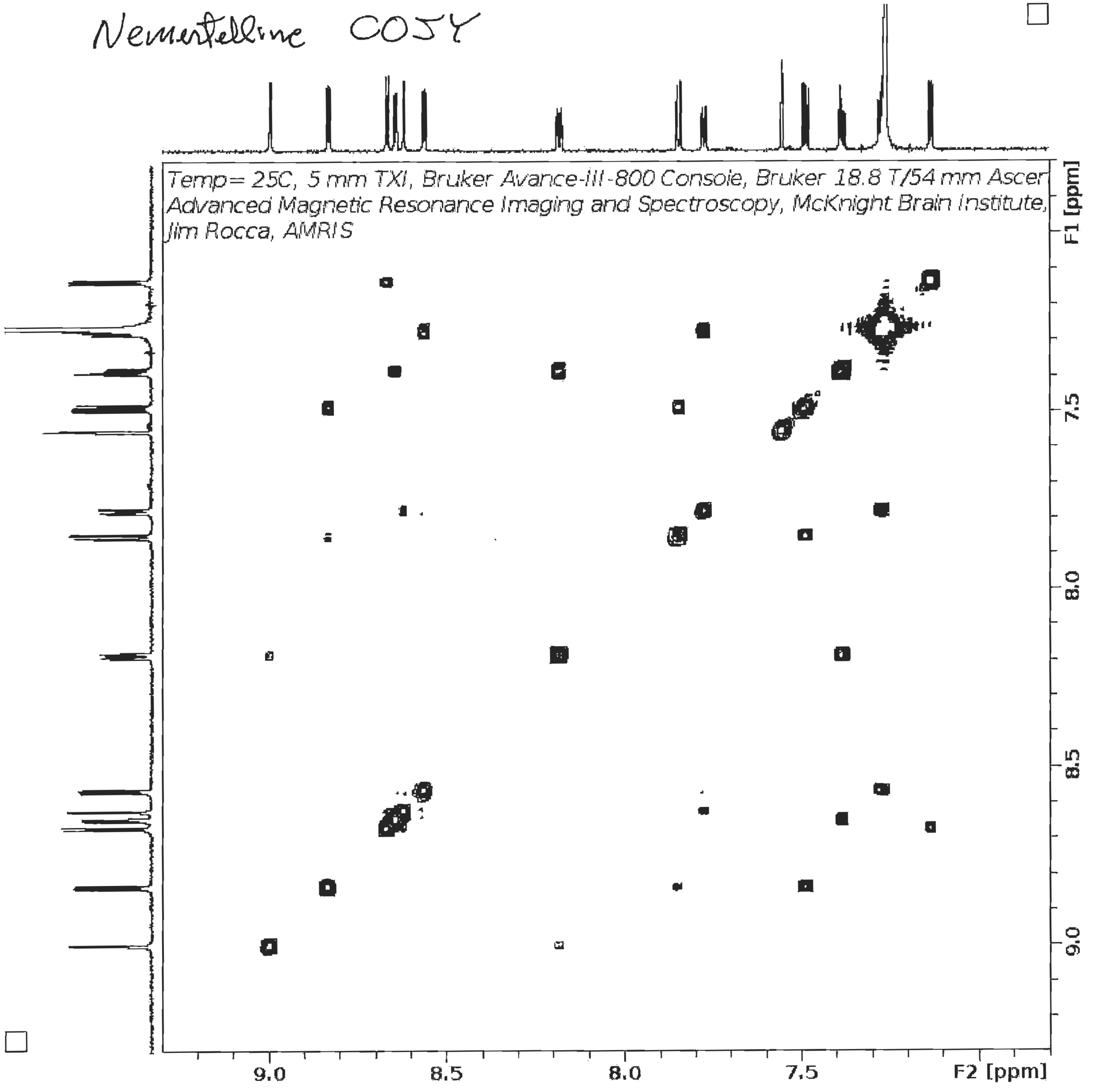

Nemertelline, HSQC Narrow Bandwidth

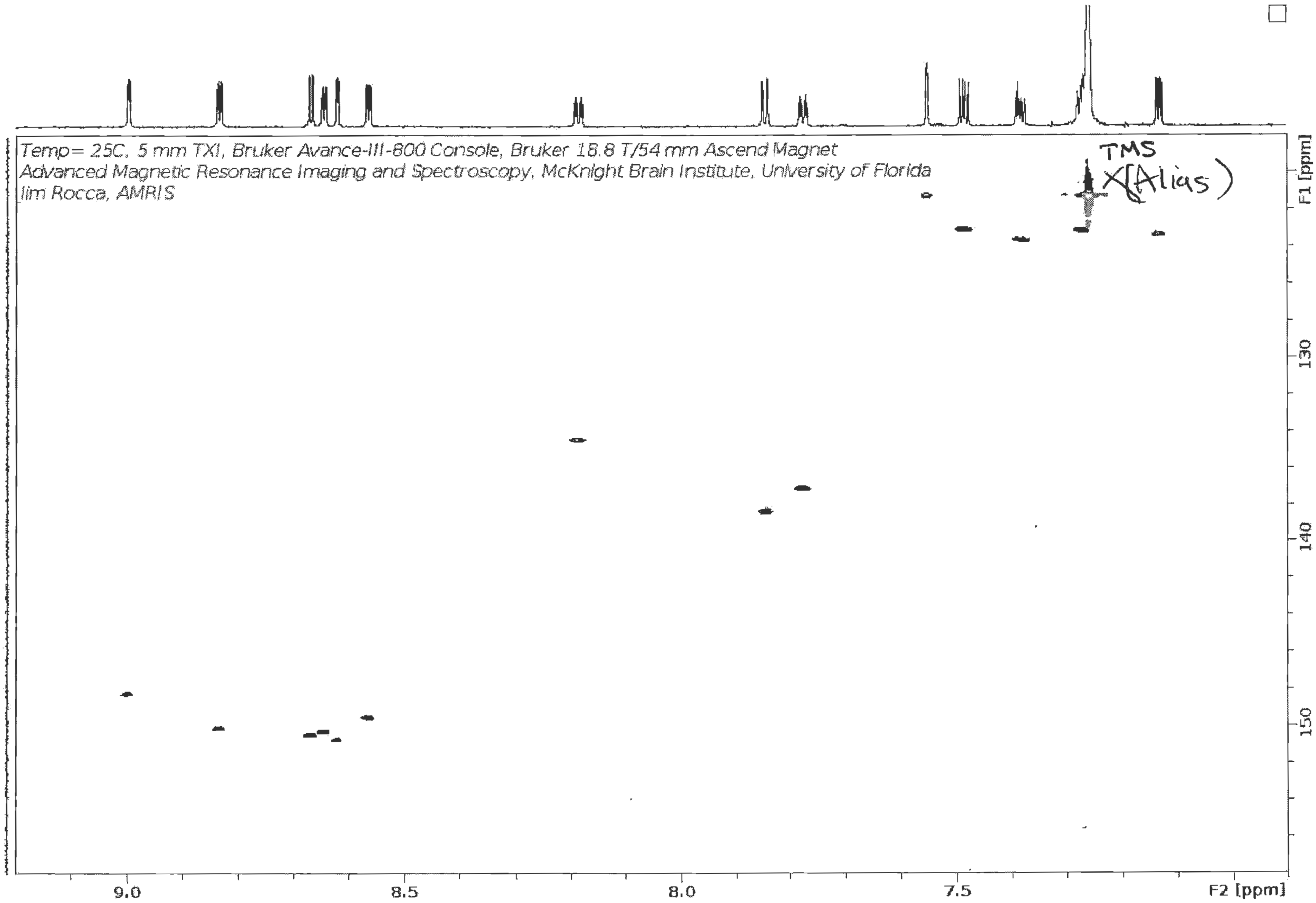

S34

Nemertelline HMBC (Full bandwith)

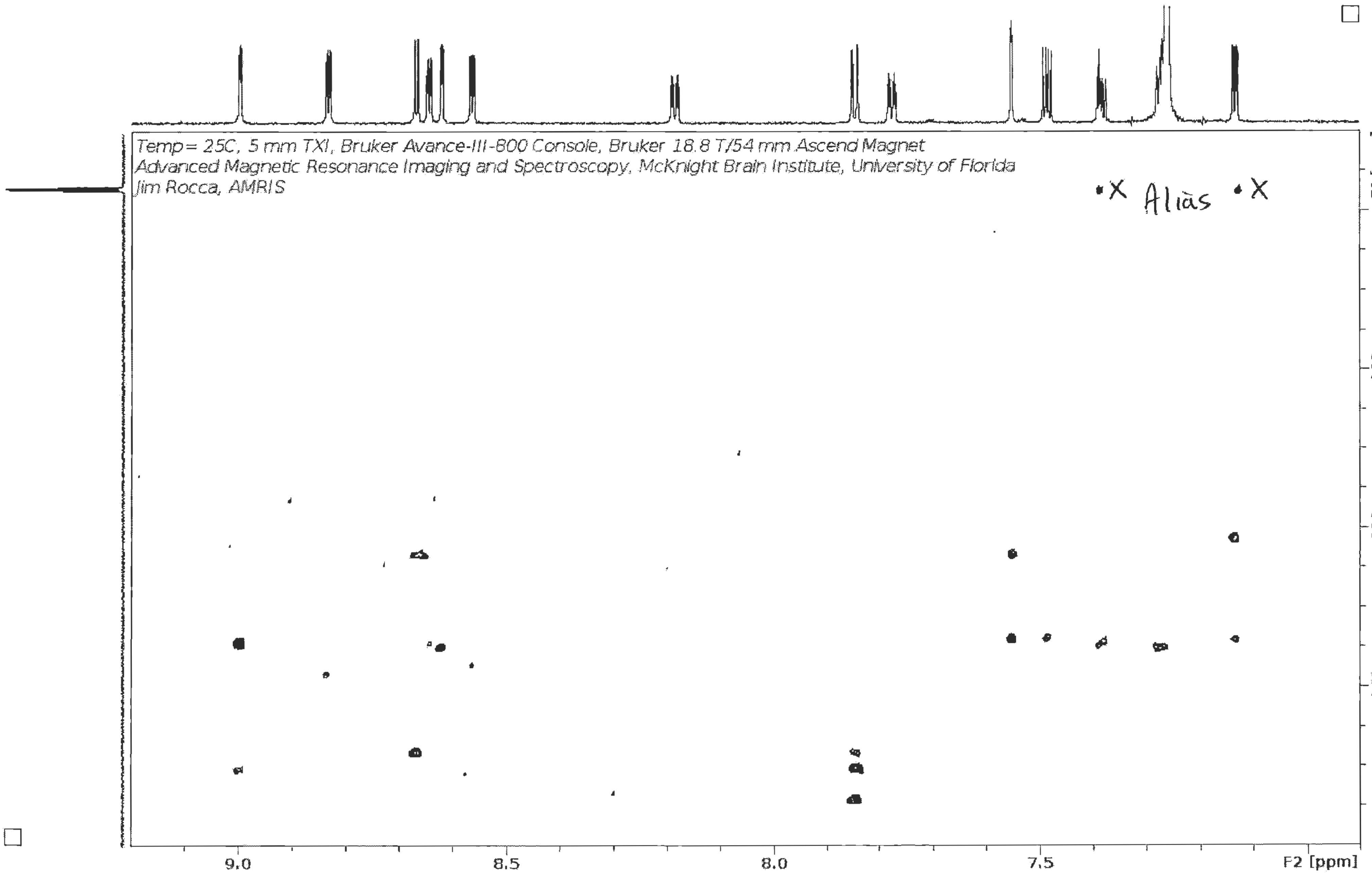

Nemertelline HMBC
(Narrow Bandwith)

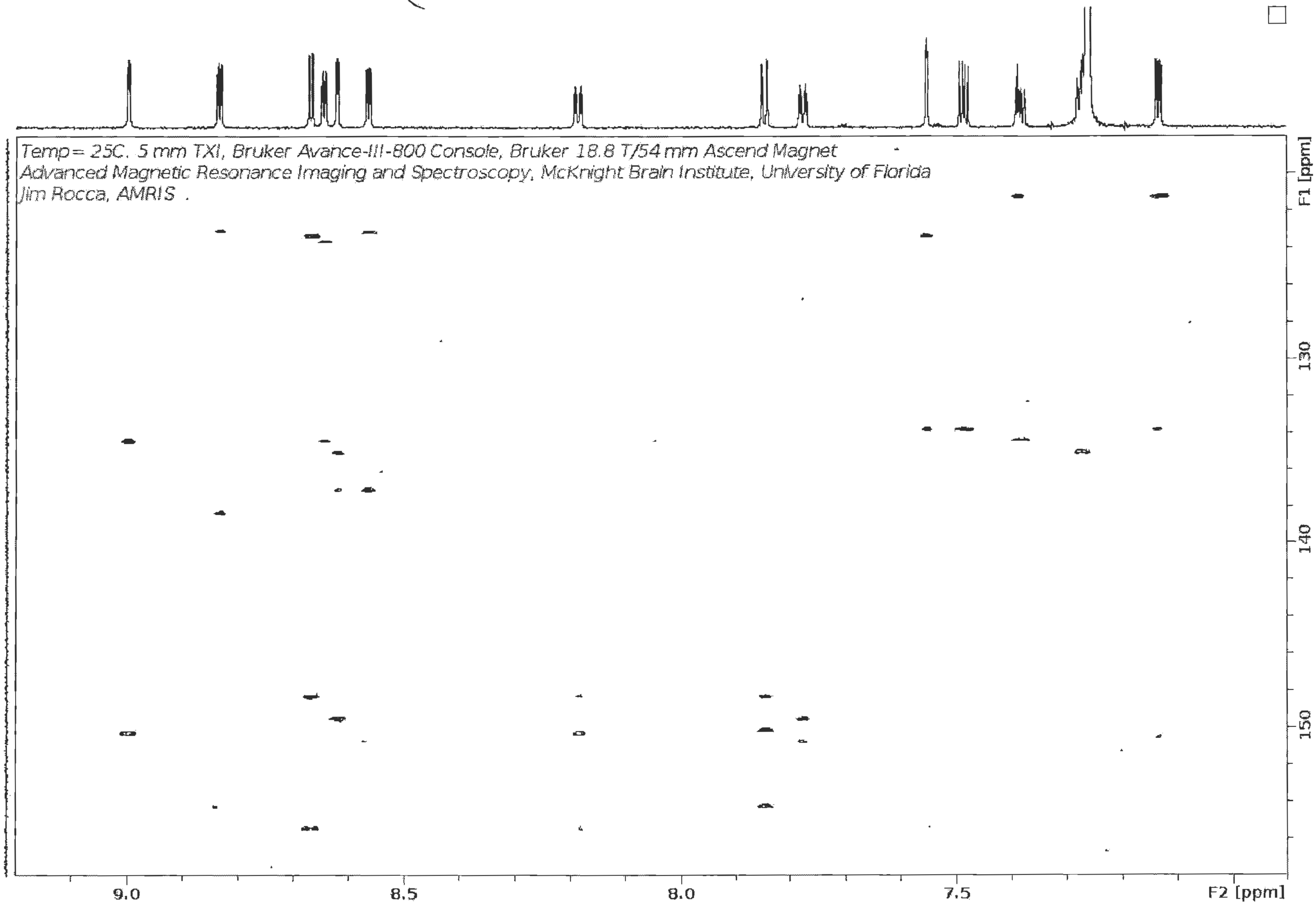

S36

Nemertelline (Natural Product) Proton and Carbon Correlations

Proton Numbering (See Reference 24)	Proton Chemical Shifts (ppm)	Carbon Chemical Shifts (ppm)	HMBC Correlations to Carbons (ppm)
H2‴	9.00	148.19	150.24 (C6‴), 134.43 (C4‴)
H6′	8.83	150.07	138.33 (C4′), 123.04 (C5′)
H6″	8.67	150.42	155.4 (C2″Q),148.25 (C4″Q),123.33(C5″)
H6‴	8.65	150.24	134.43 (C4‴), 123.62 (C5‴)
H2	8.62	150.67	149.45 (C6), 135.0 (C3Q), 137.09 (C4)
H6	8.56	149.45	137.09 (C4), 123.12 (C5)
H4‴	8.19	134.43	155.4 (C2″Q), 150.24(C6‴), 148.19(C2‴)
H4′	7.85	138.33	154.2 (C2′Q), 150.07, 148.25 (C4″Q)
H4	7.78	137.09	150.67 (C2), 149.45 (C6)
H3″	7.55	121.23	133.8 (C3′Q), 123.33 (C5″)
H5′	7.49	123.04	133.8 (C3′Q)
H5‴	7.38	123.62	134.43 (C4‴)
H5	7.27	123.12	135.0 (C3Q)
H5″	7.13	123.33	133.8 (C3′Q), 121.23 (C3″)

NMR data obtained on a sub-milligram quantity of natural Nemertelline acquired at 800 MHz in 180 µl of CDCl3. Proton and carbon chemical shifts are in ppm from internal TMS at 0.0 ppm. The letter Q indicates a quaternary carbon along with its particular position. All carbons except quaternary C3‴ could be tentatively assigned by our HSBC and HMBC correlation data. We speculate that the chemical shift of C3‴ is in the vicinity of 134.2 to 134.4 ppm, based upon Cruskie et al [24] reporting only one carbon with a chemical shift of 134.36 ppm, whereas Bouillon et al [25]reported two chemical shifts of 134.22 and 134.26 ppm. We only detected one carbon in that range, namely C4‴ at 134.43 by way of HSQC and HMBC correlation with $^{n}J_{CH}$ = 8 Hz.
